# Cancer morbidity trends and regional differences in England—A Bayesian analysis

**DOI:** 10.1371/journal.pone.0232844

**Published:** 2020-05-20

**Authors:** Ayşe Arık, Erengul Dodd, George Streftaris

**Affiliations:** 1 Maxwell Institute for Mathematical Sciences, Heriot-Watt University, Edinburgh, Scotland, United Kingdom; 2 Mathematical Sciences, University of Southampton, Southampton, England, United Kingdom; South China University of Technology, CHINA

## Abstract

Reliable modelling of the dynamics of cancer morbidity risk is important, not least due to its significant impact on healthcare and related policies. We identify morbidity trends and regional differences in England for all-cancer and type-specific incidence between 1981 and 2016. We use Bayesian modelling to estimate cancer morbidity incidence at various age, year, gender, and region levels. Our analysis shows increasing trends in most rates and marked regional variations that also appear to intensify through time in most cases. All-cancer rates have increased significantly, with the highest increase in East, North West and North East. The absolute difference between the rates in the highest- and lowest-incidence region, per 100,000 people, has widened from 39 (95% CI 33-45) to 86 (78-94) for females, and from 94 (85-104) to 116 (105-127) for males. Lung cancer incidence for females has shown the highest increase in Yorkshire and the Humber, while for males it has declined in all regions with the highest decrease in London. The gap between the highest- and lowest-incidence region for females has widened from 47 (42-51) to 94 (88-100). Temporal change in in bowel cancer risk is less manifested, with regional heterogeneity also declining. Prostate cancer incidence has increased with the highest increase in London, and the regional gap has expanded from 33 (30-36) to 76 (69-83). For breast cancer incidence the highest increase has occurred in North East, while the regional variation shows a less discernible increase. The analysis reveals that there are important regional differences in the incidence of all-type and type-specific cancers, and that most of these regional differences become more pronounced over time. A significant increase in regional variation has been demonstrated for most types of cancer examined here, except for bowel cancer where differences have narrowed.

## Introduction

In this study we investigate trends and differences in population cancer morbidity risk in England stratified by age, year, gender and region between 1981 and 2016 using the data available from the Office for National Statistics (ONS) [1]. We systematically employ several generalised linear model (GLM) structures including change point analysis to capture major health policy interventions for cancer incidence data in a Bayesian setting to estimate age-specific cancer morbidity rates by region and year for both genders. In addition to incidence of all-cancer types, we focus on four specific common types of cancer: malignant neoplasm of lung and neighbouring sites (trachea and bronchus), colorectal (or bowel) cancer, prostate cancer and breast cancer. These four types represent just over half of all cancer registrations in England (around 53%) [2–6]. Note that we just consider female breast cancer as there are very few registrations regarding to male breast cancer. Using Bayesian methodology we estimate age- and region-specific rates, together with associated uncertainty intervals. This allows us to identify increasing and diverging regional trends in cancer morbidity in England during the studied period.

Cancer disease remains one of the most common cause of death in the United Kingdom (UK) in recent years [7, 8]. According to the ONS, 28.1% of all deaths in England and Wales were due to cancer in 2017 [9]. Understanding trends in cancer incidence has a crucial importance given that one in two people born after 1960 in the UK will experience some sort of cancer at some point in their lifetime [10]. This has significant public health implications, including increasing treatment costs. Better understanding of cancer trends can allow necessary steps to be taken in line with public health interventions.

There is extensive literature concerning mortality and incidence of malignant neoplasms in various countries including England and the UK, with most of these studies focussing on observed trends in incidence rates and the assessment of the presence and magnitude of differences in the incidence of malignant neoplasms or associated mortality [11–20]. While the majority of the existing literature is focused on the impact of social deprivation on population- and/or cancer-specific incidences [21–29], regional variation in leading cancer morbidity risk remains less well characterised.

## Materials and methods

### Data

We use cancer registration data relating to the nine regions of England (according to Nomenclature of Territorial Units for Statistics) between 1981 and 2016 [30], obtained from the ONS [1]. The cancer registration numbers are stratified by individual site code based on the International Statistical Classification of Diseases and Related Health Problems (ICD), five-year age-bands, year, gender, and region. Two different policy changes occurred relating to ICD codes between 1981 and 2016: ICD 9 was used between 1981 and 1994, and ICD 10 was used after 1995 onwards. Annual population counts for the corresponding regions of England, as estimated by the ONS, were also obtained from the ONS.

The data cover malignant cancer registrations from age zero to 95+ (e.g. 0, 1-4, 5-9, …, 95+). As the last age group is 85+ for population estimates, we also grouped cancer registration numbers from 85+ onwards. For modelling purposes we use age as the midpoint of every five-year age-band and we take age 90 as a suitable point for the last age group. Also, age-specific incidence rates for lung, colorectal and prostate cancer are modelled from age 45 onwards (i.e. 45-49, 50-54, …, 85+), while the age range for all-cancer and breast cancer rates is taken from age 15 onwards. This is based on the observed rates for certain types being almost zero before the given ages, and is also supported by the ONS and Cancer Research UK publications which point out that some of the most common cancer types, e.g. lung and bowel, mostly occur at age 50 and later, while other types, e.g. breast cancer, affect younger age groups (e.g. age 15 onwards) [6, 31].

**Fig 1:**
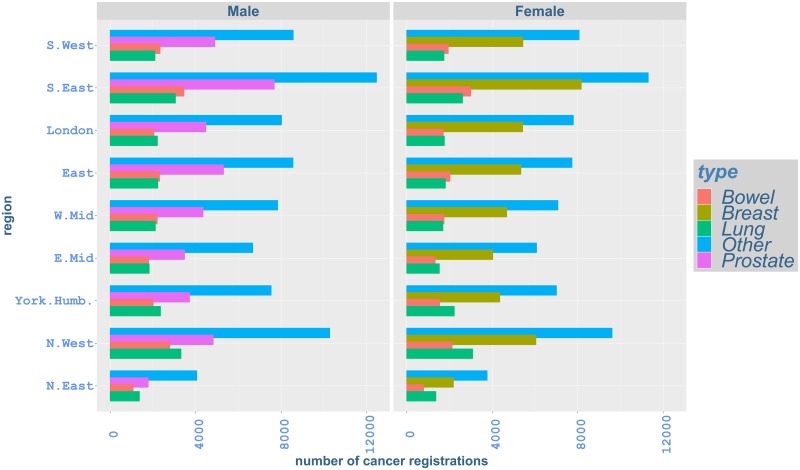
Cancer registrations by cancer type and region, England, 2016.

**Fig 2:**
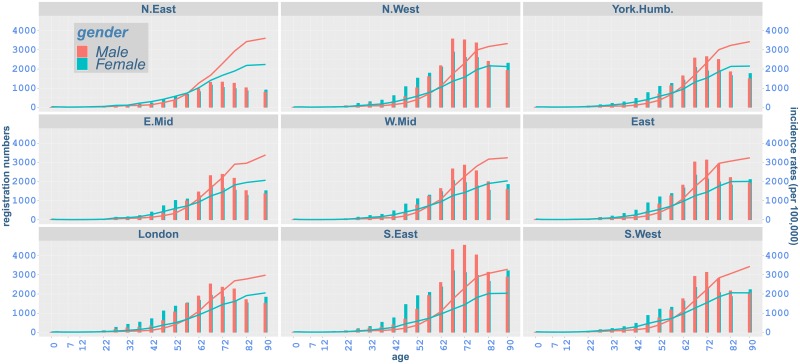
Age-specific cancer registrations (bars) and observed incidence rates (lines) per 100,000 people by regions of England, 2016.

The distribution of cancer registration numbers by major cancer type and region in the most recent year of available data (2016) is shown in Fig 1 and demonstrates that prostate and breast cancer, with 40,489 cases registered for prostate cancer and 45,656 for breast cancer, are the most common cancers diagnosed in that year [6]. The distribution of cancer registrations with respect to age and region in 2016 is also presented in Fig 2. In all regions, registration numbers increase until age around 62-72, and then generally decrease for older ages. Differences by gender can also be observed: whilst cancer registrations for females are higher before age 57 compared to males in the same age groups, males show higher cancer registration numbers for older ages in all regions. We note that in Fig 1 discussed here, only registration numbers are considered and the size of the corresponding exposed populations is not taken into account.

### Statistical modelling

We model population cancer morbidity risk using incidence rates (number of registrations in a given category, divided by the corresponding estimated population) based on Bayesian GLM structures [32, 33]. It is common to assume a Poisson distribution for cancer registration counts. However, the implicit assumption under the Poisson distribution, that the mean is equal to the variance, might be very restrictive for large heterogeneous populations such as these considered here. Therefore, in this study we allow for overdispersion through a hierarchical Poisson-lognormal Bayesian model [34], that also allows us to incorporate uncertainty in a natural manner and to provide appropriate uncertainty intervals for estimated quantities. Details of the model are presented as follows:

$$\begin{array}{cc} C_{a,t,g,r} & ∼\text{Poisson}(\theta_{a,t,g,r}\;\;E_{a,t,g,r})\\ \theta_{a,t,g,r} & ∼\text{Lognormal}(\mu_{a,t,g,r},\sigma^{2})\\ \mu_{a,t,g,r} & =\beta X, \end{array}$$

where *C*_*a*,*t*, *g*, *r*_ is the number of cancer registrations for a given malignant neoplasm at age *a* in year *t* for gender *g* in region *r*. *E*_*a*,*t*,*g*,*r*_ and *θ*_*a*,*t*,*g*,*r*_ denote the corresponding mid-year population count (as estimated by the Office for National Statistics, ONS) and the true underlying incidence rate respectively. Vector ***β*** denotes the model coefficients, and ***X*** is the vector of covariates, specifically age, year, gender, and region, in addition to potential interaction(s) linked with cancer incidence rates in the model.

Age is used as a numerical variable, except when all-cancer rates are considered where it enters the model as a categorical variable with 15 levels {17, 22, …, 90}. As Fig 2 demonstrates, very small numbers of cancer registrations occur in the youngest age groups, while cancer incidence is mostly observed in later ages. Hence, we start the modelling from age 17 onwards for all-cancer incidence. The same age starting point (17) is also considered for breast cancer, while for all other specific cancer types modelling is carried out from age 47 onwards, following the literature and cancer statistics in the UK [3, 5, 6, 31, 35–39].

Year of registration is also used as a numerical variable in the analysis, while gender and region are taken as categorical variables. Numerical variables are standardised to have zero mean and unit variance to facilitate the computations. For categorical variables we use a sum-to-zero constraint on their model coefficients, which implies an average-effect reference level for comparison purposes.

Non-informative prior distributions are assumed for all model parameters to reflect relative prior ignorance on their values. More specifically, we use

$$\begin{array}{cc} \beta_{\text{j}} & ∼\text{Normal}(0,10^{4}),\;\;\;\text{for}\;\;\;j=1,2,\ldots ,P\\ \sigma^{2} & ∼\text{Inverse-Gamma}(1,0.001), \end{array}$$

where *P* is the number of coefficients in each analysis. Markov chain Monte Carlo (MCMC) simulation techniques are implemented to derive posterior distributions and appropriate estimates of quantities of interest, using the Bayesian analysis software WinBUGS [40, 41].

Historically, major public health policy interventions, such as national screening programmes, have had the potential to affect the pattern and trend of incidence rates of malignant neoplasms [42–47]. We have therefore applied change detection analysis to identify distinct changes in the distributional properties of cancer incidence rates through time. We used a dynamic programming algorithm, namely the Pruned Exact Linear Time (PELT) method, due to its superior computational efficiency and accuracy compared to other search methods, e.g. Binary Segmentation or Segment Neighbourhood methods, using the changepoint package in R [48, 49]. We identified two break points in our analysis, in 1989 and 2006, for breast cancer and bowel cancer, respectively. Another break point is considered for breast cancer at age 50 due to epidemiological reasons in the literature [50, 51]. Details of the method and resulting models are presented in the S1 File.

For each type of cancer, several models were used for fitting incidence rates and compared in a systematic way. We only present results with the best fitted model here, which was determined for each cancer type using a Bayesian variable selection procedure in the R-INLA software [52]. The procedure is based on Bayes factors [53], and the Deviance Information Criterion (DIC) [54]. The Bayes factor for models *H*_*j*_ and *H*_*k*_ is defined as $B_{jk}=\frac{P(D|H_{j})}{P(D|H_{k})};\;\;j\neq k$, that is the ratio of posterior odds of models *H*_*j*_ and *H*_*k*_ based on data *D*, provided that same prior distributions are assumed for both models [52]. The DIC, which is calculated based on the effective number of parameters and marginal loglikelihood, can be expressed as $\text{DIC}=-4E_{\beta |D}\left(\log f\left(D|\beta \right)\right)+2\log f\left(D|\hat{\beta }\right)$, where $\hat{\beta }$ is the posterior mean, mode or median of parameter vector ***β***, and *f* is the likelihood function. Starting from the simplest model, which typically does not include any variables, a new variable is added into the model provided that it improves the fit of the model to the data significantly. More specifically, the variable appears in the best fitted model if it enables the model to imply a lower DIC [54] or a Bayes factor which is greater than 3 [53]. Table 1 shows the covariates and possible interactions included in the best fitted model for each type of cancer. All main covariates (age, year, gender and region) are added in each model, and the interaction terms in Table 1 may involve different powers of numerical variables. All included terms have a significant impact on incidence rates. Model specifications and further details, including parameter estimates, are provided in the S1 File.

**Table 1:** Covariates and interactions included in the best fitted model for each type of cancer.

Type of cancer	Highest age power	Highest year power	Age:Year	Age:Gender	Age:Region	Gender:Year	Gender:Region	Region:Year
All-cancer	1	1	yes	yes	yes	yes	yes	yes
Lung cancer	2	2	yes	yes	yes	yes	yes	yes
Bowel cancer	2	2	yes	yes	yes	yes	yes	yes
Prostate cancer	3	3	yes	no	yes	no	no	yes
Breast cancer	3	2	yes	no	yes	no	no	no

While age-specific cancer morbidity rates are estimated, age-standardised rates were also considered to facilitate summaries and comparisons. These were calculated using the European Standard Population (ESP) 2013 structure, developed by the statistical office of the European Union, Eurostat [55]. ESP 2013 was preferred to other standardisation options, such as the ESP 1976 or the WHO World Standard, due to two reasons: the capacity to reflect the current population structure more accurately by allocating a greater weight to the older population and the ability to make a fair comparison of the average risk of cancer diagnoses in different regions [4].

To identify variations in the level of morbidity across regions of England, we calculated absolute and relative differences, denoted by *AD*_*t*_ and *RD*_*t*_ respectively, between the age-standardised fitted rates in the region showing the highest incidence and the region showing the lowest incidence in each given year, for all considered types of cancer as follows:

$$\begin{array}{cc} {\text{AD}}_{t} & ={\text{HR}}_{t}-{\text{LR}}_{t}\\ {\text{RD}}_{t} & =\frac{{\text{HR}}_{t}-{\text{LR}}_{t}}{{\text{LR}}_{t}}, \end{array}$$

where HR_*t*_ is the highest rate and LR_*t*_ is the lowest rate in year *t*. Relative differences are calculated to provide reference to the overall incidence level at a particular time and therefore serve to also take into account the change in temporal trends of cancer incidence.

For quantifying the change in incidence rates between 1981 and 2016 for each cancer type, we also calculated the relative (%) change (RC) in each region as

$$\begin{array}{c} {\text{RC}}_{r}=100\times \;\frac{{\text{R}}_{2016,r}-{\text{R}}_{1981,r}}{{\text{R}}_{1981,r}}, \end{array}$$

with R_2016,*r*_ indicating the relevant age-standardised (fitted) incidence rate in region *r* in 2016 and R_1981,*r*_ showing the corresponding incidence rate in the same region in 1981.

## Results

### Incidence rate trends

**Fig 3:**
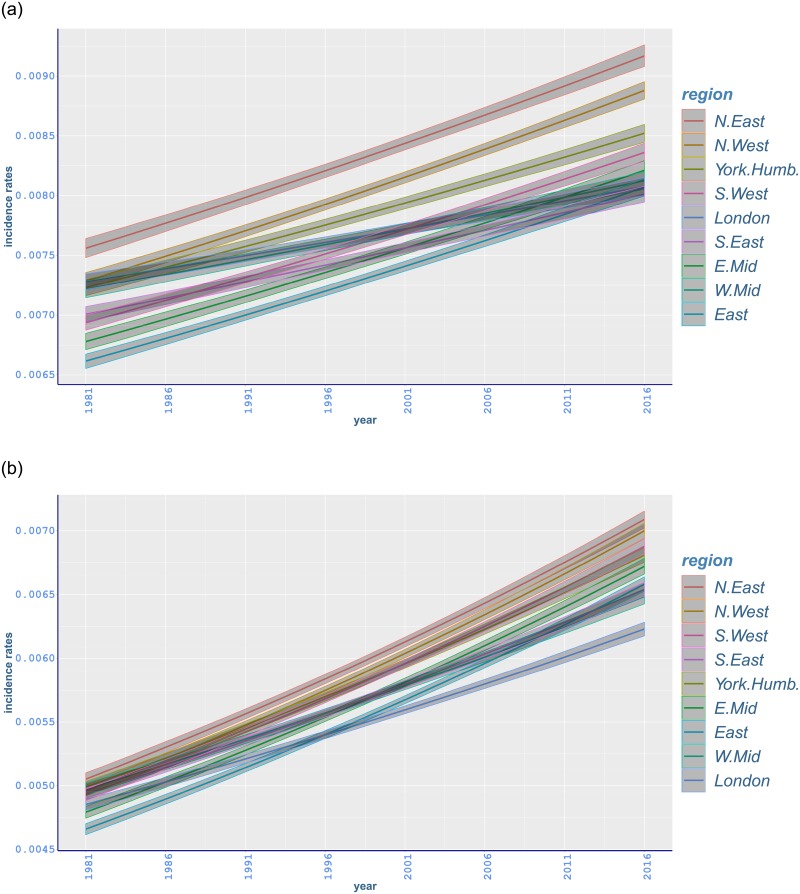
Age-standardised fitted incidence rates of malignant neoplasm of all-cancer between 1981 and 2016 in different regions of England with 95% credible intervals: (a) males; (b) females.

Age-standardised fitted rates for all-cancer incidence have an increasing trend for both genders in all regions (Fig 3). During recent years the highest incidence rates occur in North East for both genders whilst the lowest rates are mostly shown in London for females and East for males.

**Fig 4:**
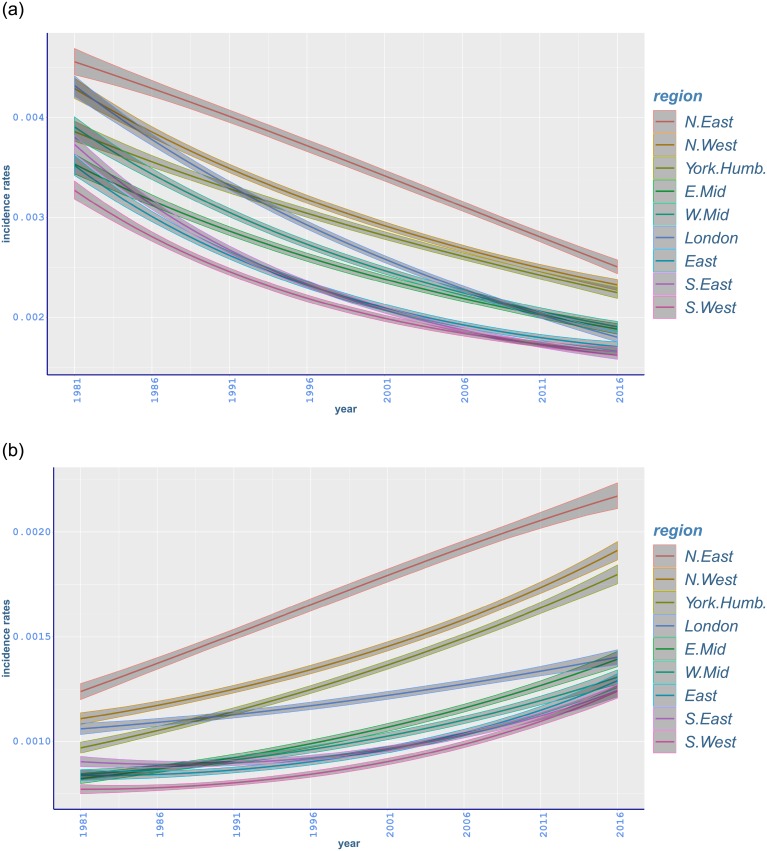
Age-standardised fitted incidence rates of malignant neoplasm of tranchea, bronchus and lung between 1981 and 2016 in different regions of England with 95% credible intervals: (a) males; (b) females.

There is an adverse trend in lung cancer for males and females (Fig 4) [56]. Whilst North East exhibits the highest incidence rates, the lowest rates mostly occur in South West for both genders over years, with a decreasing trend in male rates and an increasing trend in female counterparts. The different trends in malignant neoplasm of lung in England may be explained by the change in smoking patterns as women started smoking more and men less after the Second World War [3]. Although average daily smoking is still higher among men, the gap between smoking habits of men and women has been narrowing during the most recent years [5].

**Fig 5:**
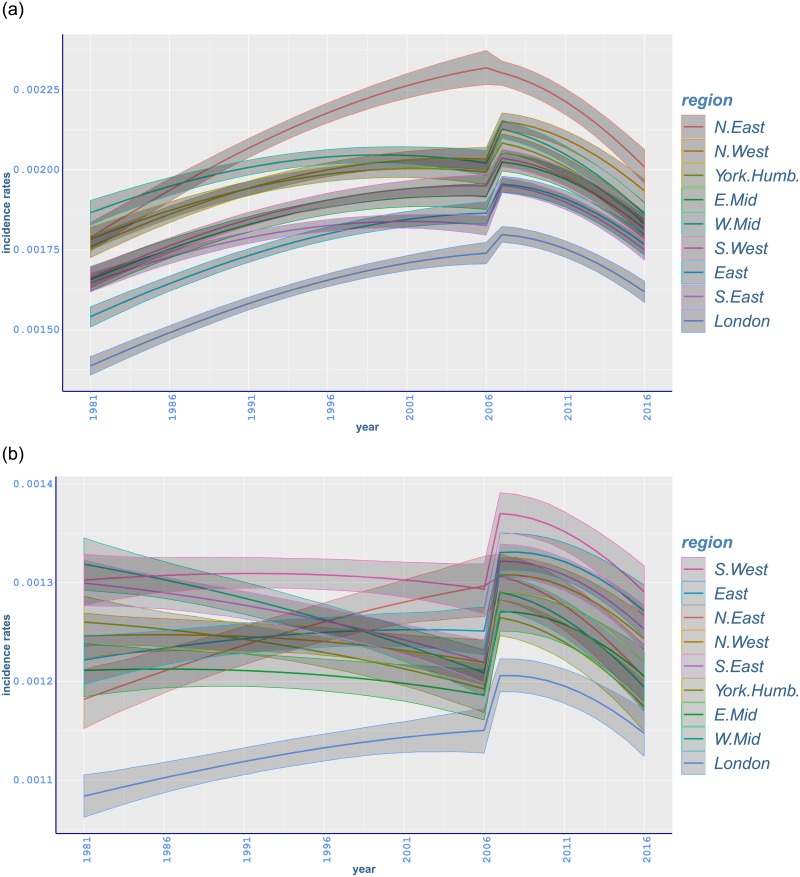
Age-standardised fitted incidence rates of malignant neoplasm of bowel between 1981 and 2016 in different regions of England with 95% credible intervals: (a) males; (b) females.

Incidence rates of bowel cancer for males have an increasing trend with the highest rates mostly in North East until 2006, although they decline in most recent years with a distinct jump around 2006 (Fig 5). This may reflect the impact of the National Bowel Cancer Screening Programme which was introduced in 2006, and also detected by our changepoint analysis. Although the initial screening age group was 60-69, the screening was extended to age 74 in 2010 [57]. Bowel cancer trends for females are more variable (Fig 5). While most regions show decreasing trends, with a distinct change around 2006, rates in North East, London and East are mostly increasing, before showing decreasing trends following the 2006 jump.

**Fig 6:**
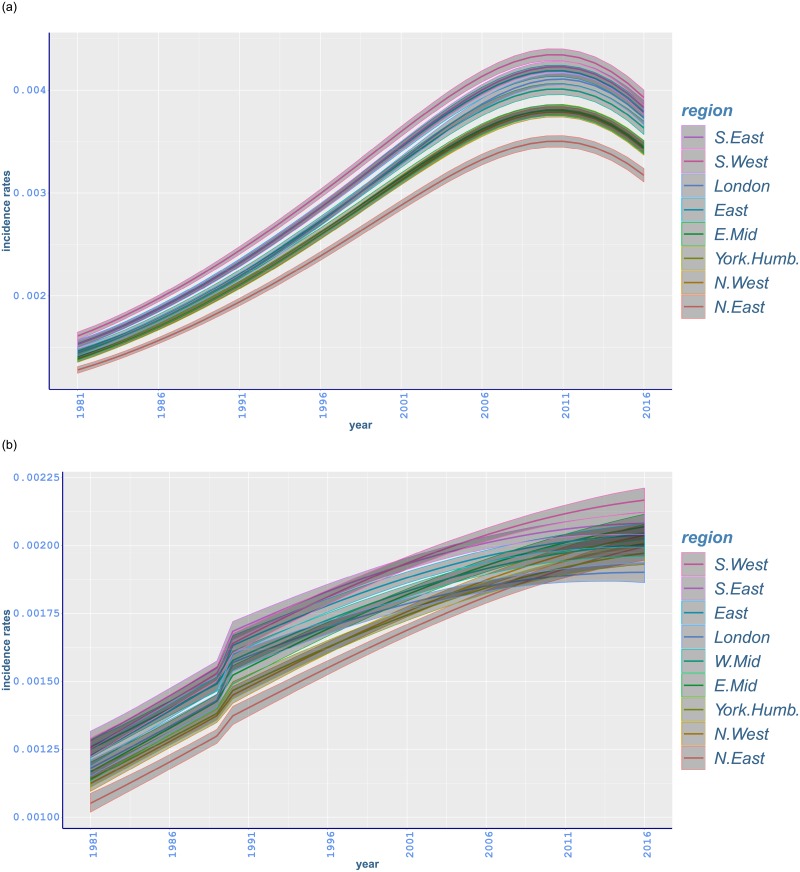
Age-standardised fitted incidence rates of malignant neoplasm of (a) prostate and (b) breast, between 1981 and 2016 in different regions of England with 95% credible intervals.

There is a steep increase in prostate cancer rates in all regions in the late 1980s and throughout the 1990s (Fig 6). A rise in the uptake of Prostate-Specific Antigen (PSA) testing, which was introduced in the UK in the late 1980s and early 1990s, is pointed out as an explanation of the increase [6, 58]. However, rates seem to decline at different rates among regions in most recent years. This may be explained by regional variation in the uptake of screening tests [39]. For most of the study period, the highest prostate cancer rates are shown in South West, while the lowest rates appear in North East.

**Fig 7:**
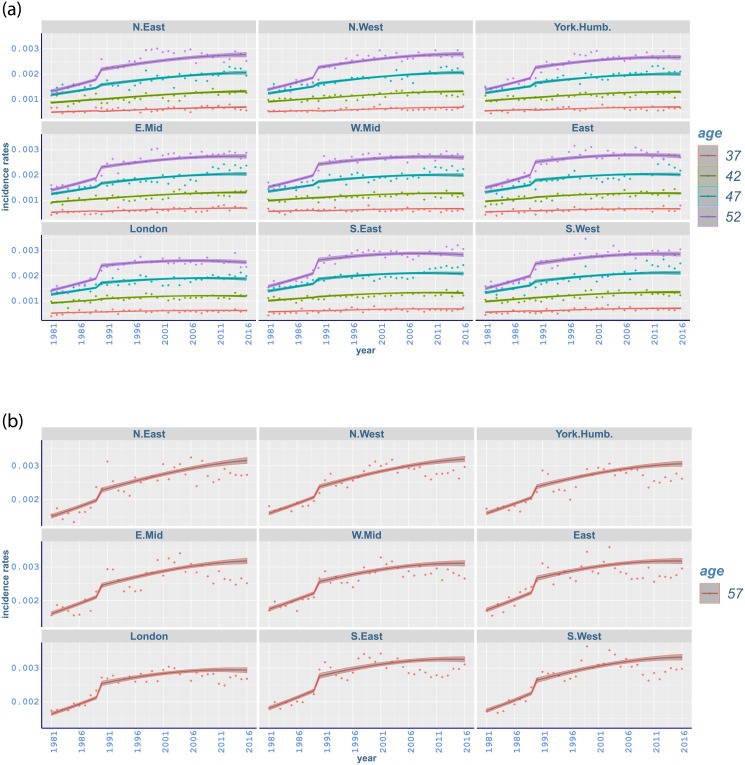
Observed and fitted incidence rates of malignant neoplasm of breast in different regions of England (a) from age 37 to 52 and (b) age 57 between 1981 and 2016 with 95% credible intervals.

**Fig 8:**
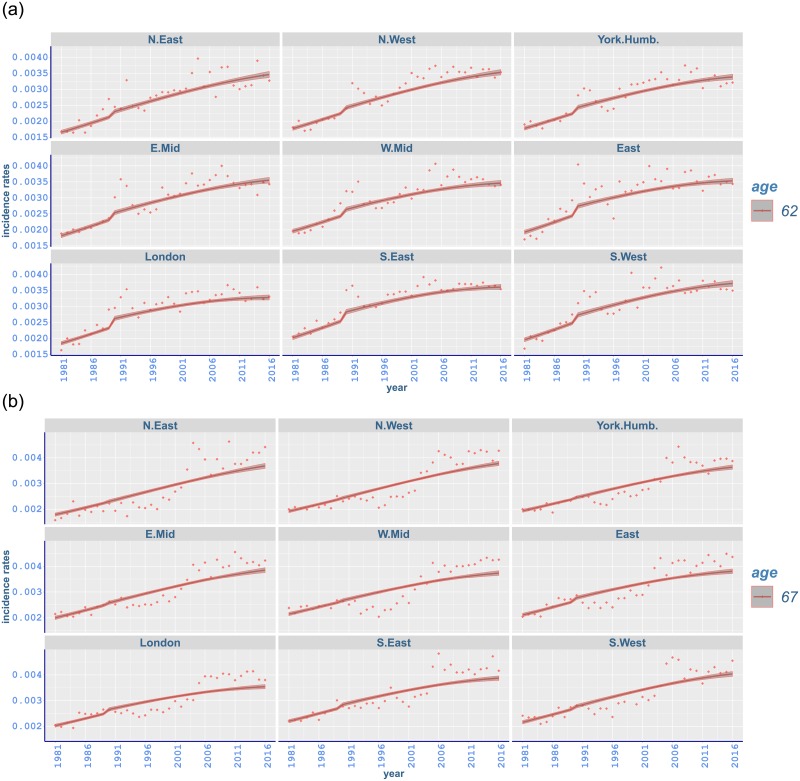
Observed and fitted incidence rates of malignant neoplasm of breast in different regions of England (a) from age 62 to (b) 67 between 1981 and 2016 with 95% credible intervals.

Breast cancer rates have a rapidly increasing trend with a jump around 1988. The highest incidence rates occur in South West and the lowest rates mostly in North East (Fig 6). While the incidence of breast cancer has a gradual increase at younger ages, there is a sudden change at age 52 starting from 1988 and going through the early 1990s in all regions (Fig 7). Similar sharp increases occur in the same period from age 57 to 62 mostly with a peak around 1991-1992 (Figs 7 and 8). Moreover, a steep increase starting in mid 1990s at age 67 has a peak around 2005 (Fig 8). This is followed by a gradual increase in subsequent years (appendix pp 47-48). The steep increase between ages 52 and 62 can possibly be attributed to the implementation of the NHS Breast Screening Programme (NHSBSP) which was initiated in 1988 and captured by our changepoint analysis in 1989. This first screening programme targeted all women between age 50 and 64, and invited them for screening once every three years. However, merely 2.5% of the target population was screened in 1988, and most of the screening units completed the prevalence round between 1989 and 1993 [59]. It is also noted that cancer incidence registrations between age 50 and 64 escalated by almost 50% from 1988 until 2006 where half of this increase is believed to happen because of the NHSBSP [60]. Furthermore, screening was extended to age 70 between 2002 and 2004, while another age expansion trial for women aged 47-73 is in operation [46].

### Regional differences

#### Regional variation for specific cancer types

Cancer morbidity varies considerably across different regions. We highlight this variation here by presenting incidence rates in the most recent year of data (2016) (Table 2), in addition to the relative change in each region from 1981 to 2016 together with appropriate 95% intervals (Table 3). The highest all-cancer incidence rates for men in 2016 happened in North East, 916 · 8(908, 926 · 1) per 100,000, and followed by considerably lower incidence rates in North West, and Yorkshire and the Humber (Table 2). Differences among the remaining regions appear to be smaller and not significant. Moreover, although all-cancer incidence of males has risen since 1981 in each region, the highest relative increases occurred in East (21·97%, 20·26 to 23·78), and followed by North West and North East with similar changes (Table 3). In Table 2, for the female population, while all-cancer incidence rates in 2016 are again highest in North East, 708 · 9(702 · 3, 715 · 5), differences among the highest-incidence regions are less pronounced than for men (Table 3 and Fig 3). On the other hand, rates in London, 622 · 9(617 · 5, 628 · 3), are significantly lower than those in all other regions (Table 2). It is also noted that all-cancer rates in London exhibit the slowest increasing trend between 1981 and 2016 (Fig 3). While all-cancer incidence of females has increased since 1981, in a similar way with males, the highest relative increases happened in East (41·18%, 39·25 to 43·18), and followed by North West and North East, respectively (Table 3).

**Table 2:** Age-standardised fitted incidence rates, per 100,000 people, in regions of England for different types of cancer in 2016; 95% credible intervals in brackets. Source of original data: ONS [1].

Region	Type of cancer							
	All		Lung		Bowel		Prostate	Breast
	Male	Female	Male	Female	Male	Female	Male	Female
**N.East**	916.8 (908,926.1)	708.9 (702.3,715.5)	250.5 (243.7,257.5)	217.1 (211.1,223.4)	200.6 (195.4,204.9)	120.8 (117.7,124)	317.2 (310.7,323.8)	199.7 (194.6,204.5)
**N.West**	888 (880.8,895.3)	700 (694.1,705.9)	232.3 (226.8,237.8)	191.2 (186.7,195.4)	193 (189.5,196.1)	124.3 (121.8,126.7)	342.9 (336.7,348.8)	203.8 (199.9,207.9)
**York.Humb**.	852.1 (844.6,859.6)	680.9 (675.4,686.9)	224.6 (218.9,230)	179.7 (175.3,184.3)	182.9 (179.3,186.7)	117.5 (115.1,119.8)	345.2 (338.8,351.2)	197.2 (193,201.3)
**E.Mid**.	821.1 (813.1,829.2)	672 (666.1,678.6)	188.2 (183.1,193.7)	139.3 (135.7,143)	181.6 (177.9,185.4)	120.7 (118.2,123.5)	344.1 (337.9,350.7)	207 (202.3,211.6)
**W.Mid**.	812.5 (805.3,820)	648.4 (642.6,654.1)	190.5 (185.5,195.8)	130.7 (127.3,134.2)	186 (182.4,189.2)	119.6 (117.2,121.9)	363.2 (356.9,369.8)	200.5 (196,205)
**East**	807 (799.8,814.2)	658 (652.6,663.9)	170.6 (166.3,175.3)	129 (125.7,132.5)	176.4 (172.8,180)	127.2 (124.5,129.8)	379.2 (372.5,386.1)	203.9 (199.4,208.1)
**London**	807.5 (800.4,815.1)	622.9 (617.5,628.3)	180 (175.3,184.6)	140.3 (136.6,143.8)	161.7 (158.6,165.8)	115 (112.8,117.6)	372.9 (366.4,379.5)	190.2 (186.3,193.8)
**S.East**	801.3 (794.6,807.9)	654 (648.9,659.6)	161.7 (158,165.4)	124.3 (121.4,127.3)	174.9 (171.6,178)	125.6 (123.1,128.3)	383 (376.5,389.9)	208.2 (204.1,212.3)
**S.West**	836.1 (828.8,843.8)	687.9 (681.9,693.8)	165.7 (161.3,170.2)	124.1 (120.7,127.3)	181.1 (177.7,184.8)	129.2 (126.6,131.9)	392.8 (385.7,400.1)	216.7 (212.2,221.2)

**Table 3:** Change in age-standardised fitted incidence rates from 1981 to 2016 (relative to 1981 rates, %) in regions of England for different types of cancer; 95% credible intervals in brackets. Source of original data: ONS [1].

Region	Type of cancer							
	All		Lung		Bowel		Prostate	Breast
	Male	Female	Male	Female	Male	Female	Male	Female
**N.East**	21.29 (19.35,23.14)	40.33 (38.14,42.4)	−45.02 (−46.73,−43.1)	75.3 (69.79,81.35)	12.09 (8.73,15.69)	2.06 (−1.03,5.38)	147.36 (140.88,154.07)	90.07 (82.16,97.74)
**N.West**	21.89 (20.18,23.48)	41.05 (39.09,42.93)	−45.84 (−47.35,−44.38)	72.45 (67.52,77.38)	9.69 (7.12,12.1)	−0.12 (−2.58,2.41)	146.48 (140.1,153.1)	81.36 (75.62,87.38)
**York.Humb**.	17.88 (16.33,19.43)	36.43 (34.64,38.16)	−41.79 (−43.46,−40.07)	85.42 (79.74,91.12)	2.48 (−0.22,5.46)	−6.69 (−9,−4.2)	148.58 (142.11,155.3)	73.03 (67.51,78.73)
**E.Mid**.	21.12 (19.28,22.97)	40.2 (38.09,42.34)	−46.74 (−48.46,−45.02)	69.47 (63.99,75.03)	9.59 (6.56,12.75)	−0.22 (−2.92,2.49)	143.85 (137.4,150.66)	77.31 (70.73,83.74)
**W.Mid**.	12.58 (11.05,14.18)	30.28 (28.43,32.11)	−51.22 (−52.67,−49.6)	55.43 (50.68,60.23)	−0.3 (−2.93,2.34)	−9.22 (−11.54,−6.94)	147.87 (141.38,154.56)	60.36 (54.76,65.75)
**East**	21.97 (20.26,23.78)	41.18 (39.25,43.18)	−51.53 (−52.97,−50.08)	54.13 (49.47,58.82)	14.43 (11.01,17.82)	4.19 (1.55,7.01)	147.01 (140.52,153.8)	66.35 (60.77,72.24)
**London**	10.91 (9.38,12.48)	28.34 (26.59,30.07)	−58.32 (−59.54,−57.01)	32.33 (28.4,36.5)	16.67 (13.69,19.89)	6.22 (3.6,8.99)	157.04 (150.6,163.78)	61.21 (55.93,66.23)
**S.East**	14.35 (12.91,15.88)	32.39 (30.71,34.03)	−56.65 (−57.87,−55.37)	37.73 (33.73,41.86)	6.1 (3.49,8.84)	−3.39 (−5.9,−0.65)	150.11 (143.67,156.81)	62.39 (57.02,67.49)
**S.West**	20.53 (18.79,22.21)	39.57 (37.61,41.52)	−49.32 (−50.84,−47.8)	60.86 (55.84,65.77)	8.97 (6.14,12.06)	−0.79 (−3.37,2.15)	143.77 (137.39,150.46)	72.58 (66.56,78.46)

Lung cancer incidence is higher in northern regions of England compared to southern regions. The results in Table 2 show that the highest lung cancer incidence occurred in North East, with rates (per 100,000) being 250·5(243·7, 257·5) for men and 217·1(211·1, 223·4) for women. This is followed by mostly significantly lower incidence in North East, and Yorkshire and the Humber, respectively, for both genders. The lowest incidence occurred in South East, 161·7(158, 165·4), for men and in South West, 124·1(120·7, 127·3), for women, with South West and East having comparable rates (Fig 4). Differences between the highest- and lowest-incidence regions seem wider compared to all-cancer incidence (Fig 4). Compared to 1981, lung cancer incidence rates for men decreased in each region with the highest relative decrease occurring in London (−58·32%, −59·54 to −57·01), while the incidence rates for women increased with the highest relative increment in Yorkshire and the Humber (85·42%, 79·74 to 91·12) (Table 3).

Bowel cancer incidence rates for males in 2016 are highest in North East, 200·6(195·4, 204·9) with comparable rates in North West (Table 2). In contrast, the highest incidence rates for females are shown in south of England, specifically in South West, 129·2(126·6, 131·9), followed by South East and East having similar rates (Table 2). Although the lowest bowel cancer incidence for both males and females occur in London, less regional inequality can be noted compared to all-cancer and lung cancer (Fig 5). The relative change in bowel cancer from 1981 to 2016 is not statistically significant in many cases (Table 3).

The highest incidence of prostate cancer in 2016 is manifested in South West, 392·8(385·7, 400·1), followed by comparable incidence rates in South East and East (Table 2). Less regional variation is noted for prostate cancer rates compared to all-cancer, lung or bowel rates, while the increase in the incidence of prostate cancer seems to be slowing down in recent years (Fig 6). Also, prostate cancer incidence has increased in each region since 1981 with the highest relative increase in London (157·04%, 150·06 to 163·78) (Table 3).

In a similar manner, south of England has the highest incidence of breast cancer. Particularly, the highest incidence in 2016 is observed in South West, 216·7(212·2, 221·2), followed by lower incidence in South East and East Midlands (Table 2). The lowest-incidence region is London, 190·2(186·3, 193·8), followed by Yorkshire and the Humber, and North East with comparable rates. Regional variations for breast cancer is less pronounced compared to other types of cancer (Fig 6). Also, breast cancer incidence has increased compared to 1981 with the highest relative increase in North East (90·07%, 82·16 to 97·74) (Table 3).

#### Regional variation trends

Importantly, regional variation appears to be increasing through time. Absolute differences between rates in regions with highest and lowest incidence, increased from 1981 to 2016 for most cancer types for both males and females (Table 4). A notable exception is malignant neoplasm of bowel for both genders, where regional variation has dropped significantly. The highest change occurs in lung cancer incidence for females, with the regional variation increasing in absolute terms by 47 (from 47(42, 51) to 94(88, 100)) per 100,000 people, and in all-cancer incidence for females by 47 (from 39(33, 45) to 86(78, 94)). This is followed by the increase of regional variation in prostate cancer (43), ranging from 33(30, 36) to 76(69, 83). Regional variation seems to drop considerably in absolute terms for lung cancer in males, declining by 40 (from 129(112, 145) to 89(81, 97)). The variation decrease in malignant neoplasm of bowel for males, i.e. 9 (from 48(44, 52) to 39(33, 44)), is nearly at the same level as that for female counterparts, i.e. 10 (from 24(21, 26) to 14(11, 17)) (Table 4).

**Table 4:** Absolute and relative differences between age-standardised fitted rates in the highest- and the lowest-incidence region for different types of cancer in 1981 and 2016; 95% credible intervals in brackets. Source of original data: ONS [1].

Type of cancer	Absolute difference (per 100,000 people)			
	Male		Female	
	1981	2016	1981	2016
**All-cancer**	94 (85,104)	116 (105,127)	39 (33,45)	86 (78,94)
**Lung cancer**	129 (112,145)	89 (81,97)	47 (42,51)	94 (88,100)
**Bowel cancer**	48 (44,52)	39 (33,44)	24 (21,26)	14 (11,17)
**Prostate cancer**	33 (30,36)	76 (69,83)		
**Breast cancer**			23 (19,28)	27 (21,32)
**Relative differences**				
**All-cancer**	0.14 (0.13,0.16)	0.14 (0.13,0.16)	0.08 (0.07,0.1)	0.14 (0.12,0.15)
**Lung cancer**	0.39 (0.34,0.45)	0.55 (0.5,0.61)	0.61 (0.54,0.67)	0.76 (0.7,0.82)
**Bowel cancer**	0.35 (0.31,0.38)	0.24 (0.2,0.28)	0.22 (0.19,0.25)	0.12 (0.1,0.15)
**Prostate cancer**	0.26 (0.23,0.28)	0.24 (0.22,0.26)		
**Breast cancer**			0.22 (0.18,0.27)	0.14 (0.11,0.17)

Relative measures of regional variation are additionally considered in our analysis, to also account for changing levels of cancer incidence through time. For these, the absolute difference between age-standardised rates in regions with highest and lowest incidence is divided by the lowest rate in each year (Table 4). Relative variation increased significantly between 1981 and 2016 for all-cancer for females and lung cancer incidence for both genders. However, relative regional differences declined for malignant neoplasm of bowel, while the regional variation decline for breast cancer rates was only marginally significant (Table 4). In the case of bowel cancer incidence, the drop in variation appears to be due to an overall convergence between highest and lowest rates (Fig 5). The decrease in relative variation breast cancer rates seems to also reflect the steep increasing trend of these rates between 1981 and 2016 (Fig 6).

#### Regional variation by age and region

**Fig 9:**
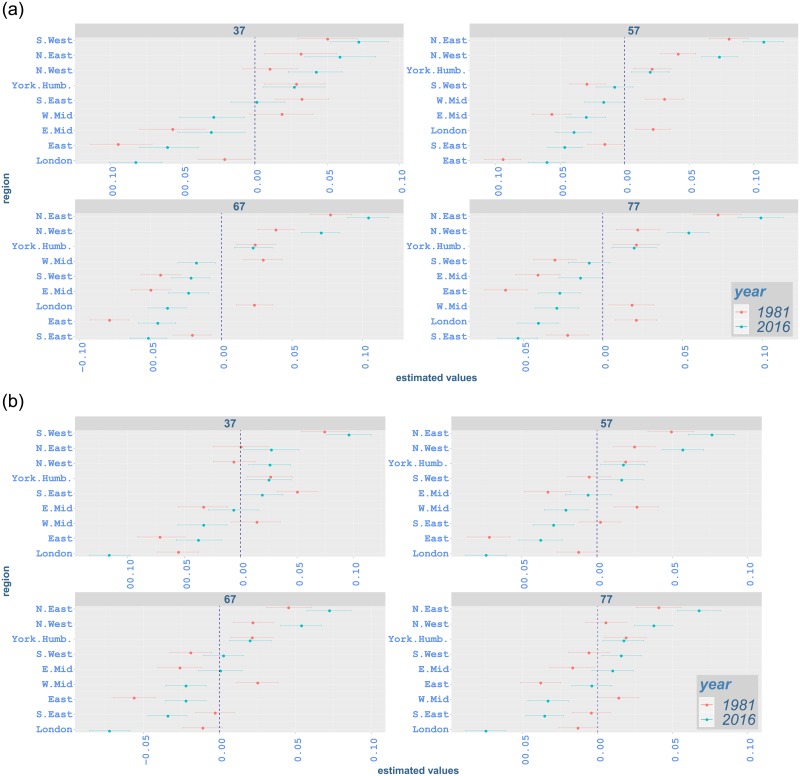
Contribution of each region to all-cancer incidence rates from age 37 to 77 in 1981 and 2016 with 95% credible intervals: (a) males; (b) females. The vertical line indicates the average regional effect.

**Fig 10:**
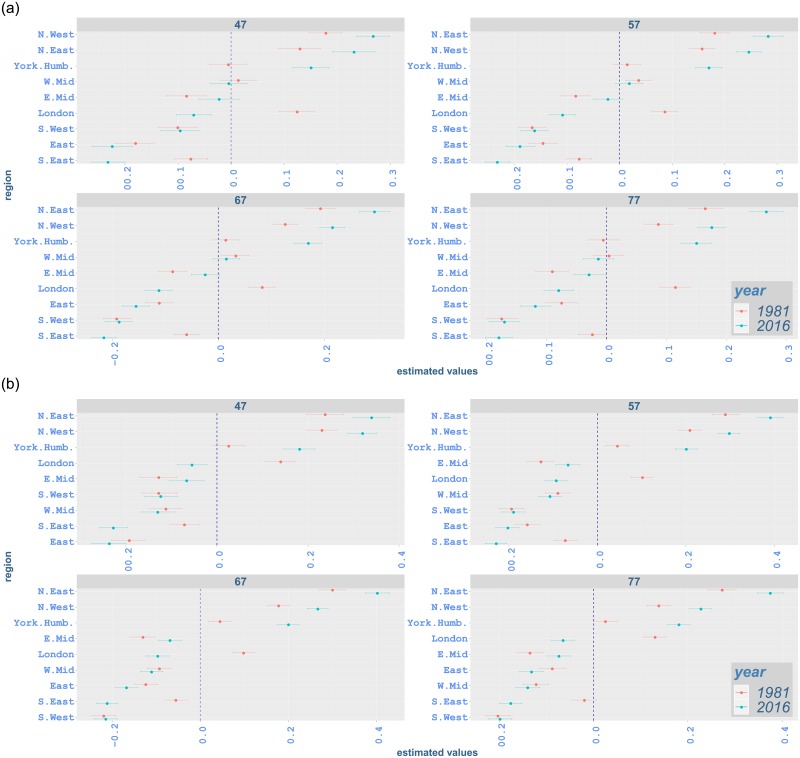
Contribution of each region to incidence rates of malignant neoplasm of tranchea, bronchus and lung from age 47 to 77 in 1981 and 2016 with 95% credible intervals: (a) males; (b) females. The vertical line indicates the average regional effect.

**Fig 11:**
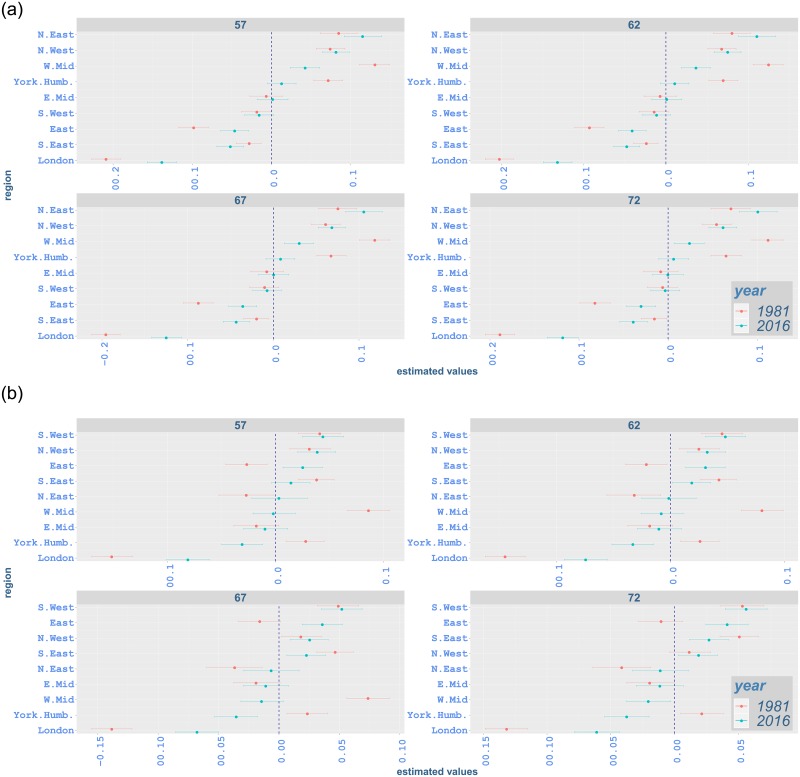
Contribution of each region to incidence rates of malignant neoplasm of bowel from age 57 to 72 in 1981 and 2016 with 95% credible intervals: (a) males; (b) females. The vertical line indicates the average regional effect.

**Fig 12:**
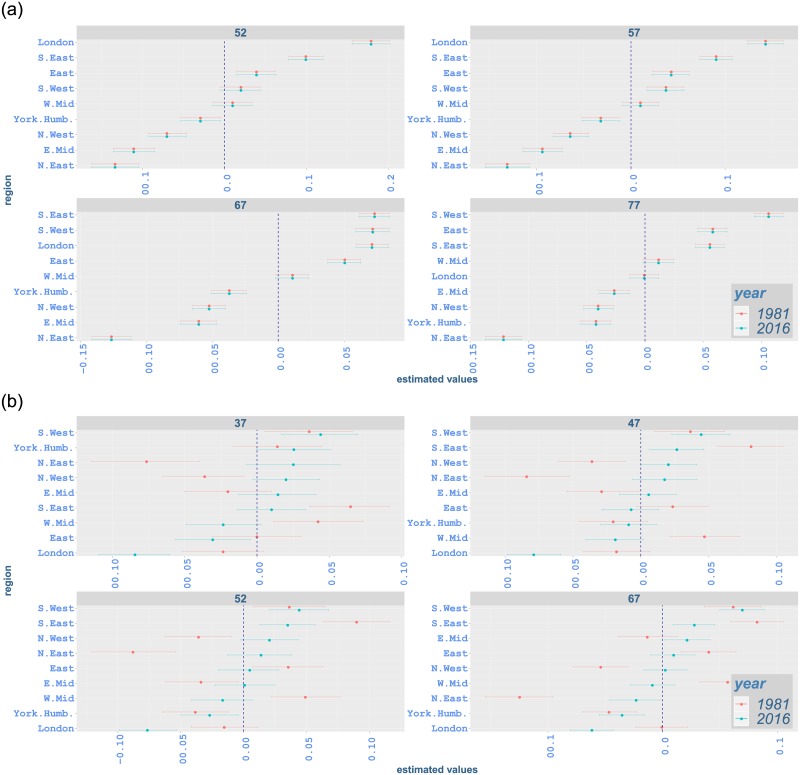
Contribution of each region to incidence rates of malignant neoplasm of prostate and breast from age 52 or 37 to 77 in 1981 and 2016 with 95% credible intervals: (a) prostate; (b) breast. The vertical line indicates the average regional effect.

Our analysis also highlights regional variation by age for different types of cancer. Figs 9–12 illustrate proportional differentials between the incidence rate for each region and the average regional effect (which is set to zero), together with corresponding 95% uncertainty intervals, for years 1981 and 2016. For example, a 0·05 value on these graphs indicates a region with a rate increased by 5% as compared to the average for all regions.

Across all ages, northern regions (North East and North West) have an excessive effect on all-cancer, lung and bowel cancer incidence for both genders in 2016 (Figs 9, 10 and 11). One exception occurs in bowel cancer rates for females, where North East shows an effect near the regional average for most ages (Fig 11). North East and North West show lower-than-average effect for prostate cancer incidence while southern regions and London mostly exceed the average effect significantly (Fig 12). The regional variation for breast cancer incidence appears considerably lower compared to other cancer types, with the effect of a number of regions (including North East and North West) not being significantly different from the average effect (Fig 12). This is in contrast with lung cancer rates, where regional effects vary considerably from the average and among them for all ages (Fig 10).

Considering age-specific rates for all-cancer incidence, South West shows an increased effect in comparison to the average at age 37, 7·1%(5·2, 9·3) for males and 9·6%(7·6, 11·6) for females, in 2016, while it has a significantly lower impact for males at age 67, −2·1%(−3·5, −0·8). On the other hand, although South West continues to have lower effect for males at age 57, i.e. −0·8%(−2·2, 0·6), and age 77, i.e. −0·9%(−2·2, 0·4), the effect for these age groups is not significantly different from the average regional impact. For females aged 57 and 77, South West shows a significantly increased effect, i.e. 1·6%(0·2, 3·1) and 1·6%(0·2, 2·9), respectively, on all-cancer incidence. South East has a significant lower impact on all-cancer incidence from age 57 onwards for both genders. While East has a lower-than-average impact on all-cancer incidence from age 37 onwards, its impact at age 77 is not statistically significant (Fig 9). South West, South East and East show lower effect on lung cancer incidence for both genders in comparison to the regional average (Fig 10). Although these regions mostly have a lower effect on bowel cancer incidence for males, their influence for females is generally above the overall mean effect, indicating excess incidence rates (Fig 11). For prostate cancer incidence, South East and East display a significantly increased effect, while the impact of South West changes from average to higher-than-average from age 52 to age 77 (Fig 12). South East alsomostly has excessive impact on breast cancer incidence. Whilst South West has an increased impact for breast cancer in all age groups, the impact of East is less clear and ranges from below-average to average levels.

London exhibits a lower effect on all-cancer incidence for both genders in 2016 (Fig 9). It shows lower impact on lung cancer incidence (Fig 10). It also has lower-than-average impact on bowel cancer incidence (Fig 11), while it shows an increased effect on prostate cancer incidence and a lower effect on breast cancer incidence (Fig 12).

East Midlands shows below-average effect on all-cancer incidence for males in 2016. However, its effect on female incidence is less clearly manifested (i.e. non-significant). West Midlands also shows a lower effect for both genders (Fig 9). While East Midlands usually have a lower effect on lung cancer incidence for both genders, the impact of West Midlands is below-average for females, but not significant for males. We also note that Yorkshire and the Humber have an excess effect on all-cancer and lung cancer incidence for both genders (Figs 9 and 10). West Midlands and Yorkshire and the Humber display increased effect on bowel cancer incidence for males, with the impact of the latter region becoming smaller as age increases. These regions mostly show a lower-than-average effect on bowel cancer rates for females. For bowel cancer incidence, the effect of East Midlands seems not to be marginally different from the average for females (Fig 11). The West Midlands region mostly has an average effect on prostate cancer incidence, while East Midlands and Yorkshire and the Humber have a lower effect (Fig 12). Regarding breast cancer, the impact of East Midlands is generally close to the average regional effect, while West Midlands and Yorkshire and the Humber show generally low effects with varying statistical significance (Fig 12).

The changes in regional variation between 1981 and 2016 (Figs 9–12) are consistent with the differences in age-standardised rates in Table 4. For all-cancer rates (Fig 9), variation around the average regional effect increases considerably in 2016 for all ages, mainly due to a significant relative decrease of the effect of London. Lung cancer also shows increased variation (Fig 10), which appears to be attributed mostly to northern areas contributing more in 2016. For bowel cancer rates, there is a drop in regional variation in 2016, which seems to be mainly due to London’s effect being closer to the average (Fig 11). Prostate cancer rates exhibit comparable overall variation in 1981 and 2016 (Fig 12). Finally, the decrease in regional variation for breast cancer rates in 2016 (Fig 12), seems to be largely explained by the effect of North East and North West moving closer to the average effect, despite the London contribution decreasing away from the mean.

## Discussion

We have analysed cancer registration rates in England by region, age, gender, and year of registration, considering four common types of cancer in addition to all-cancer type. The analysis has found that age-specific cancer incidence has generally increased in all regions in the period from 1981 to 2016 for all cancer types, with the exception of lung cancer in males. Also, the findings point towards significant regional differences in cancer incidence, with the inequalities increasing over the study period in absolute terms for all-cancer rates (in both males and females), lung cancer in females and prostate cancer. When regional variation is considered in relative terms, by also taking into account the general increasing trend in cancer morbidity rates, all-cancer rates in females and lung cancer rates in both males and females still exhibit increasing regional inequalities. Only bowel cancer incidence regional variation shows a significant decrease over time (both in absolute and relative terms), while regional differences for breast cancer rates do not appear significantly changed (Table 4).

Our findings show that malignant neoplasms of lung and bowel are more common in the northern regions of England, whereas incidence rates of malignant neoplasm of prostate and breast are lowest among all cancer types in these regions. Also, there are more pronounced regional differences in malignant neoplasm of lung, bowel, and all-cancer in comparison to malignant neoplasm of prostate and breast. These findings may possibly point towards underlying socioeconomic regional differences, while also reflect changing demographic dynamics.

A number of published studies suggest that incidence rates of malignant neoplasm of lung for both genders in England are higher in more deprived areas [24, 26, 28, 61, 62]. At the same time, the distribution of deprivation in regions of England in 2013 reveals that merely 7·3% of the population in South East lived in the most deprived quintile, whereas 32·8% of the population in North West lived in the most deprived areas [18]. Our analysis shows that the highest incidence rates of lung cancer occur in northern regions of England, which coincide considerably with more deprived areas. Similar association exists in malignant neoplasm of bowel, where the impact of socioeconomic deprivation on screening and survival of colorectal cancer patients has also been noted [21, 25, 63–65]. The relationship between social deprivation and breast cancer has been examined by assessing trends in incidence rates according to various socioeconomic categories in several studies [22, 23, 27, 66]. Higher incidence rates, linked with decreasing deprivation, are noted in these studies. Quantification of the contribution of different regions to cancer-specific incidence can help to identify and bring forward targeted public health interventions. Our study shows higher incidence rates of breast cancer in southern regions of England, where most of the population lives in least deprived areas. At the same time, we find an increasing relative contribution of northern areas to breast cancer incidence, which perhaps can be attributed to higher awareness and screening participation. Incidence rates of prostate cancer are also higher in the south of England compared to other regions. Socio-economic differences may affect the uptake of screening tests and access to high-quality health care. These factors are important for cancer diagnosis and can have a crucial role in cancer disease being diagnosed at earlier stages.

A strength of our study is that we have analysed population cancer incidence rates using more than three decades of data, which allows a comprehensive assessment. A Bayesian setting was used to estimate age-specific incidence rates by gender and region for different neoplasms to incorporate the uncertainty in various years. Our modelling also took into account the impact of national screening programs on incidence rates by carrying out relevant changepoint analyses. As a result, two temporal break points were identified, in 2006 and 1989 for malignant neoplasm of bowel and breast, respectively. A further break point was considered for breast cancer with respect to age at registration, at age 50, following similar analysis driven by epidemiological reasons in the literature. Specifically, menstrual and reproductive events are counted among the most significant risk factors for breast cancer as these factors determine the level of estrogen secretion and availability [50]. The steep increase in the incidence of breast cancer seems to slow down after the menopause (ages 40-50) [51]. A limitation of changepoint analyses can be the inability to account for possible changes happening towards the end of the considered period, as more observations are needed to identify any relevant changes. We also note that the ONS reports state that the estimated completeness of the available data is 98% due to the continuing accrual of late registrations that can take up to 5 years for a given calendar year [6]. Therefore, it is possible that cancer registration numbers in years 2015 onwards may be affected slightly when late registrations are involved.

## Supporting information

S1 File (PDF)
