# Supplementary material for: Cancer morbidity trends and regional differences in England—A Bayesian analysis
Source: PLoS One. 2020 May 20;15(5):e0232844. doi: 10.1371/journal.pone.0232844 (PMC7239391; doi:10.1371/journal.pone.0232844)
Supplement: S1 File — (PDF) [file pone.0232844.s001.pdf]

# Supporting Information

**Cancer morbidity trends and regional differences in England – a Bayesian analysis**

**Ayşe Arık, Erengul Dodd, George Streftaris**

# Table of contents

**1 Change point analysis** **7**

**2 Model for all-cancer incidence rates** **7**

**3 Model for incidence rates of malignant neoplasm of trachea, bronchus and lung** **8**

**4 Model for incidence rates of malignant neoplasm of colorectal (bowel)** **8**

**5 Model for incidence rates of malignant neoplasm of prostate** **9**

**6 Model for incidence rates of malignant neoplasm of breast** **10**

## List of Tables

1 **S1 Table. Estimated coefficients for the best fitting model for all-cancer rates.** . . . . . . . 12

2 **S2 Table. Estimated coefficients for the best fitting model for tranchea, bronchus and lung cancer rates.** . . . . . . . 22

3 **S3 Table. Estimated coefficients for the best fitting model for bowel cancer rates.** . . . . . . . 31

4 **S4 Table. Estimated coefficients for the best fitting model for prostate cancer rates.** . . . . . . . 39

5 **S5 Table. Estimated coefficients for the best fitting model for breast cancer rates.** . . . . . . . 44

## List of Figures

1 **S1 Fig. All-cancer incidence (excluding carcinoma in situ and skin cancer) for males, ages 17-27: observed rates (dots), fitted rates (lines) on log-scale with 95% credible intervals for the fitted rates.** . . . . . . . 14

2 **S2 Fig. All-cancer incidence (excluding carcinoma in situ and skin cancer) for males, ages 32-37: observed rates (dots), fitted rates (lines) on log-scale with 95% credible intervals for the fitted rates.** . . . . . . . 14

3 **S3 Fig. All-cancer incidence (excluding carcinoma in situ and skin cancer) for males, ages 42-57: observed rates (dots), fitted rates (lines) on log-scale with 95% credible intervals for the fitted rates.** . . . . . . . 15

4 **S4 Fig. All-cancer incidence (excluding carcinoma in situ and skin cancer) for males, ages 62-77: observed rates (dots), fitted rates (lines) on log-scale with 95% credible intervals for the fitted rates.** . . . . . . . . . . 15

5 **S5 Fig. All-cancer incidence (excluding carcinoma in situ and skin cancer) for males, ages 82-90: observed rates (dots), fitted rates (lines) on log-scale with 95% credible intervals for the fitted rates.** . . . . . . . . . . 16

6 **S6 Fig. All-cancer incidence (excluding carcinoma in situ and skin cancer) for females, ages 17-37: observed rates (dots), fitted rates (lines) on log-scale with 95% credible intervals for the fitted rates.** . . . . . . . . . . 16

7 **S7 Fig. All-cancer incidence (excluding carcinoma in situ and skin cancer) for females, ages 42-57: observed rates (dots), fitted rates (lines) on log-scale with 95% credible intervals for the fitted rates.** . . . . . . . . . . 17

8 **S8 Fig. All-cancer incidence (excluding carcinoma in situ and skin cancer) for females, ages 62-77: observed rates (dots), fitted rates (lines) on log-scale with 95% credible intervals for the fitted rates.** . . . . . . . . . . 17

9 **S9 Fig. All-cancer incidence (excluding carcinoma in situ and skin cancer) for females, ages 82-90: observed rates (dots), fitted rates (lines) on log-scale with 95% credible intervals for the fitted rates.** . . . . . . . . . . 18

10 **S10 Fig. All-cancer incidence (excluding carcinoma in situ and skin cancer) for males, age 52: fitted rates.** . . . . . . . . . . . . . . . . . . . . . . 18

11 **S11 Fig. All-cancer incidence (excluding carcinoma in situ and skin cancer) for females, age 52: fitted rates.** . . . . . . . . . . . . . . . . . . . . . 19

12 **S12 Fig. All-cancer incidence (excluding carcinoma in situ and skin cancer) for males, age 67: fitted rates.** . . . . . . . . . . . . . . . . . . . . . . 19

13 **S13 Fig. All-cancer incidence (excluding carcinoma in situ and skin cancer) for females, age 67: fitted rates.** . . . . . . . . . . . . . . . . . . . . . 20

14 **S14 Fig. All-cancer incidence (excluding carcinoma in situ and skin cancer) for males, year 1981 and 2016: observed rates (dots), fitted rates (lines) on log-scale, with 95% credible intervals for the fitted rates.** . . . 20

15 **S15 Fig. All-cancer incidence (excluding carcinoma in situ and skin cancer) for females, year 1981 and 2016: observed rates (dots), fitted rates (lines) on log-scale, with 95% credible intervals for the fitted rates.** 21

16 **S16 Fig. Tranchea, bronchus and lung cancer incidence for males, ages 47-57: observed rates (dots), fitted rates (lines) on log-scale, with 95% credible intervals for the fitted rates.** . . . . . . . . . . . . . . . . . . . . . . 23

17 **S17 Fig. Tranchea, bronchus and lung cancer incidence for males, ages 62-67: observed rates (dots), fitted rates (lines) on log-scale, with 95% credible intervals for the fitted rates.** . . . . . . . . . . . . . . . . . . . . . . 23

18 **S18 Fig. Tranchea, bronchus and lung cancer incidence for males, ages 72-77: observed rates (dots), fitted rates (lines) on log-scale, with 95% credible intervals for the fitted rates.** . . . . . . . . . . . . . . . . . . . . . . 24

19 **S19 Fig. Tranchea, bronchus and lung cancer incidence for males, age 82: observed rates (dots), fitted rates (lines) on log-scale, with 95% credible intervals for the fitted rates.** . . . . . . . . . . . . . . . . . . . . . . . . . 24

20 **S20 Fig. Tranchea, bronchus and lung cancer incidence for males, age 90: observed rates (dots), fitted rates (lines) on log-scale, with 95% credible intervals for the fitted rates.** . . . 25

21 **S21 Fig. Tranchea, bronchus and lung cancer incidence for females, ages 47-57: observed rates (dots), fitted rates (lines) on log-scale, with 95% credible intervals for the fitted rates.** . . . 25

22 **S22 Fig. Tranchea, bronchus and lung cancer incidence for females, ages 62-67: observed rates (dots), fitted rates (lines) on log-scale, with 95% credible intervals for the fitted rates.** . . . 26

23 **S23 Fig. Tranchea, bronchus and lung cancer incidence for females, ages 72-77: observed rates (dots), fitted rates (lines) on log-scale, with 95% credible intervals for the fitted rates.** . . . 26

24 **S24 Fig. Tranchea, bronchus and lung cancer incidence for females, ages 82-90: observed rates (dots), fitted rates (lines) on log-scale, with 95% credible intervals for the fitted rates.** . . . 27

25 **S25 Fig. Tranchea, bronchus and lung cancer incidence for males, age 72: fitted rates.** . . . 27

26 **S26 Fig. Tranchea, bronchus and lung cancer incidence for females, age 72: fitted rates.** . . . 28

27 **S27 Fig. Tranchea, bronchus and lung cancer incidence for males, age 77: fitted rates.** . . . 28

28 **S28 Fig. Tranchea, bronchus and lung cancer incidence for females, age 77: fitted rates.** . . . 29

29 **S29 Fig. Tranchea, bronchus and lung cancer incidence for males, year 1981 and 2016: observed rates (dots), fitted rates (lines) on log-scale, with 95% credible intervals for the fitted rates.** . . . 29

30 **S30 Fig. Tranchea, bronchus and lung cancer incidence for females, year 1981 and 2016: observed rates (dots), fitted rates (lines) on log-scale, with 95% credible intervals for the fitted rates.** . . . 30

31 **S31 Fig. Bowel cancer incidence for males, ages 47-57: observed rates (dots), fitted rates (lines) on log-scale, with 95% credible intervals for the fitted rates.** . . . 32

32 **S32 Fig. Bowel cancer incidence for males, ages 62-67: observed rates (dots), fitted rates (lines) on log-scale, with 95% credible intervals for the fitted rates.** . . . 32

33 **S33 Fig. Bowel cancer incidence for males, ages 72-82: observed rates (dots), fitted rates (lines) on log-scale, with 95% credible intervals for the fitted rates.** . . . 33

34 **S34 Fig. Bowel cancer incidence for males, age 90: observed rates (dots), fitted rates (lines) on log-scale, with 95% credible intervals for the fitted rates.** . . . 33

35 **S35 Fig. Bowel cancer incidence for females, ages 47-57: observed rates (dots), fitted rates (lines) on log-scale, with 95% credible intervals for the fitted rates.** . . . 34

36 **S36 Fig. Bowel cancer incidence for females, ages 62-67: observed rates (dots), fitted rates (lines) on log-scale, with 95% credible intervals for the fitted rates.** . . . . . 34

37 **S37 Fig. Bowel cancer incidence for females, ages 72-82: observed rates (dots), fitted rates (lines) on log-scale, with 95% credible intervals for the fitted rates.** . . . . . 35

38 **S38 Fig. Bowel cancer incidence for females, age 90: observed rates (dots), fitted rates (lines) on log-scale, with 95% credible intervals for the fitted rates.** . . . . . 35

39 **S39 Fig. Bowel cancer incidence for males, age 62: fitted rates.** . . . . . 36

40 **S40 Fig. Bowel cancer incidence for females, age 62: fitted rates.** . . . . . 36

41 **S41 Fig. Bowel cancer incidence for males, age 67: fitted rates.** . . . . . 37

42 **S42 Fig. Bowel cancer incidence for females, age 67: fitted rates.** . . . . . 37

43 **S43 Fig. Bowel cancer incidence for males, year 1981 and 2016: observed rates (dots), fitted rates (lines) on log-scale, with 95% credible intervals for the fitted rates.** . . . . . 38

44 **S44 Fig. Bowel cancer incidence for females, year 1981 and 2016: observed rates (dots), fitted rates (lines) on log-scale, with 95% credible intervals for the fitted rates.** . . . . . 38

45 **S45 Fig. Prostate cancer incidence, ages 47-57: observed rates (dots), fitted rates (lines) on log-scale, with 95% credible intervals for the fitted rates.** . . . . . 40

46 **S46 Fig. Prostate cancer incidence, ages 62-77: observed rates (dots), fitted rates (lines) on log-scale, with 95% credible intervals for the fitted rates.** . . . . . 40

47 **S47 Fig. Prostate cancer incidence, age 82: observed rates (dots), fitted rates (lines) on log-scale, with 95% credible intervals for the fitted rates.** 41

48 **S48 Fig. Prostate cancer incidence, age 90: observed rates (dots), fitted rates (lines) on log-scale, with 95% credible intervals for the fitted rates.** 41

49 **S49 Fig. Prostate cancer incidence, age 62: fitted rates.** . . . . . 42

50 **S50 Fig. Prostate cancer incidence, age 72: fitted rates.** . . . . . 42

51 **S51 Fig. Prostate cancer incidence, year 1981 and 2016: observed rates (dots), fitted rates (lines) on log-scale, with 95% credible intervals for the fitted rates.** . . . . . 43

52 **S52 Fig. Breast cancer incidence, age 17: observed rates (dots), fitted rates (lines) on log-scale, with 95% credible intervals for the fitted rates.** 45

53 **S53 Fig. Breast cancer incidence, ages 22-32: observed rates (dots), fitted rates (lines) on log-scale, with 95% credible intervals for the fitted rates.** . . . . . 45

54 **S54 Fig. Breast cancer incidence, ages 37-52: observed rates (dots), fitted rates (lines) on log-scale, with 95% credible intervals for the fitted rates.** . . . . . 46

55 **S55 Fig. Breast cancer incidence, ages 57-62: observed rates (dots), fitted rates (lines) on log-scale, with 95% credible intervals for the fitted rates.** . . . . . 46

56 **S56 Fig. Breast cancer incidence, age 67: observed rates (dots), fitted rates (lines) on log-scale, with 95% credible intervals for the fitted rates.** 47

57 **S57 Fig. Breast cancer incidence, age 72: observed rates (dots), fitted rates (lines) on log-scale, with 95% credible intervals for the fitted rates.** 47

58 **S58 Fig. Breast cancer incidence, age 77: observed rates (dots), fitted rates (lines) on log-scale, with 95% credible intervals for the fitted rates.** 48

59 **S59 Fig. Breast cancer incidence, ages 82-90: observed rates (dots), fitted rates (lines) on log-scale, with 95% credible intervals for the fitted rates.** . . . . . 48

60 **S60 Fig. Breast cancer incidence, age 57: fitted rates.** . . . . . 49

61 **S61 Fig. Breast cancer incidence, age 67: fitted rates.** . . . . . 49

62 **S62 Fig. Breast cancer incidence, year 1981 and 2016: observed rates (dots), fitted rates (lines) on log-scale, with 95% credible intervals for the fitted rates.** . . . . . 50

# 1 Change point analysis

As health screening programmes have the potential to affect the pattern and trend of incidence rates, we apply changepoint analysis, so that we can take into account important changes in the distributional properties of the dataset. Changepoint analysis was implemented using the *changepoint* package in R software.[1] The package provides three different changepoint algorithms: binary segmentation, segment neighbourhoods and the pruned exact linear time (PELT). We used the PELT method due to its computational efficiency and accuracy.[2] Before carrying out changepoint analysis, a suitable distributional assumption, which is required for the main function in changepoint package, is made based on chi-square goodness of fit test. Furthermore, a modified version of the Bayes information criterion, compatible with changepoint analysis, is used as penalised likelihood method.[3]

# 2 Model for all-cancer incidence rates

The all-cancer incidence rates are modelled from age 17 onwards. We regress the covariates on the location parameter of the lognormal distribution as follows:

$$\mu_{a,t,g,r} = \beta_0 + \beta_{1,a} + \beta_2 t + \beta_{3,g} + \beta_{4,r} + \beta_{5,a} t + \beta_{6,a,g} + \beta_{7,g} t + \beta_{8,a,r} + \beta_{9,r} t + \beta_{10,g,r}.$$

Here, age is a categorical variable with levels $a = 1, 2, \ldots, 15$, where $a$ maps to $\{17, 22, \ldots, 90\}$ and $\beta_{1,a}$ denoting the corresponding coefficients, respectively; year, denoted by $t$, is a numerical variable with $t \in \{1981, \ldots, 2016\}$; gender is a categorical variable with levels $g = 1, 2$, where $g$ maps to $\{\text{Male}, \text{Female}\}$ and $\beta_{3,g}$ denoting the relevant coefficients, respectively; and region is a categorical variable with levels $r = 1, \ldots, 9$, where $r$ maps to {North East, North West, Yorkshire and the Humber, East Midlands, West Midlands, East, London, South East and South West} and $\beta_{4,r}$ showing the corresponding coefficients, respectively. Furthermore, interactions between age and other covariates, more specifically year, $\beta_{5,a}t$, gender, $\beta_{6,a,g}$, and region, $\beta_{8,a,r}$, are considered in addition to interactions between gender and two other covariates, i.e. year, $\beta_{7,g}t$, and region, $\beta_{10,g,r}$.

Fig 1-15 show a generally increasing trend in all-cancer incidence for both genders and for all ages. Some changes that may possibly be linked to screening programs can be observed. For instance, there is a steep increase after age 50 in all-cancer incidence for males after mid 1990s (Fig 3). This may also be related to the malignant neoplasm of prostate which is the most common type of cancer for males.[4] Moreover, the decrease in the most recent years after age 50 might also be linked to the decrease of the incidence of prostate cancer starting from 2013 (Fig 3-5). In a similar way, the steep increase in female incidence rates (e.g. age 67 in Fig 8) could be related to a change in breast screening policy in the early 2000s which directly affects women over age 64. The incidence rates for males increase substantially after age 60 compared to the incidence rates for females in the same age group (Fig 4-5 for males and Fig 8-9 for females).

# 3 Model for incidence rates of malignant neoplasm of trachea, bronchus and lung

Malignant neoplasm of lung and neighbouring sites are modelled from age 47 onwards. We regress the covariates on the location parameter of lognormal distribution as given below:

$$\mu_{a,t,g,r} = \beta_0 + \beta_1 a + \beta_2 a^2 + \beta_3 t + \beta_4 t^2 + \beta_{5,g} + \beta_{6,r} + \beta_7 at + \beta_8 a^2 t + \beta_9 at^2 + \beta_{10,g} a + \beta_{11,g} a^2 + \beta_{12,g} t + \beta_{13,r} a + \beta_{14,r} a^2 + \beta_{15,r} t + \beta_{16,r} t^2 + \beta_{17,g,r},$$

where age, $a$, is numerical with $a \in \{47, 52, \dots, 90\}$ and year, $t$, is also numerical. Note that different powers of age (up to 2) and different powers of year (up to 2) appear in the best fitting model, in addition to various interaction terms. More specifically, two-way interaction terms appear between age and year, e.g. $\beta_7 at$; age and gender, e.g. $\beta_{10,g} a$; gender and year, e.g. $\beta_{12,g} t$; age and region, e.g. $\beta_{13,r} a$; region and year, e.g. $\beta_{15,r} t$; and gender and region, e.g. $\beta_{17,g,r}$.

Observed and fitted incidence rates of malignant neoplasm of trachea, bronchus and lung in England are presented in Fig 16-30 from age 47 to 90 for both genders between 1981 and 2016. There is an adverse trend in lung cancer for males and females. The different trends are more obvious after age 67 (Fig 18-20 for males and Fig 23-24 for females). Despite the fact that there is a decreasing trend for all ages of males in all regions, the age-specific rates for females are mostly increasing with a steeper trend after age 60 (Fig 22-24).

# 4 Model for incidence rates of malignant neoplasm of colorectal (bowel)

Malignant neoplasm of bowel is modelled from age 47 onwards. We regress the covariates on the location parameter of lognormal distribution as given below:

$$\mu_{a,t,g,r} = \beta_0 + \beta_1 a + \beta_2 a^2 + \beta_{3,1} t_1 + \beta_{3,2} t_2 + \beta_{4,1} t_1^2 + \beta_{4,2} t_2^2 + \beta_{5,g} + \beta_{6,r} + \beta_{7,1} at_1 + \beta_{7,2} at_2 + \beta_{8,1} at_1^2 + \beta_{8,2} at_2^2 + \beta_{9,1} a^2 t_1 + \beta_{9,2} a^2 t_2 + \beta_{10,1} a^2 t_1^2 + \beta_{10,2} a^2 t_2^2 + \beta_{11,g} a + \beta_{12,g} a^2 + \beta_{13,1,g} t_1 + \beta_{13,2,g} t_2 + \beta_{14,1,g} t_1^2 + \beta_{14,2,g} t_2^2 + \beta_{15,r} a + \beta_{16,g,r} + \beta_{17,1,r} t_1 + \beta_{17,2,r} t_2,$$

where age, denoted by $a$, is numerical with $a \in \{47, 52, \dots, 90\}$. Based on changepoint analysis, a break point is considered in year 2006. Therefore, year, denoted by $t_1$ and $t_2$, show numerical variables where $t_1 \le 2006$ and $t_2 > 2006$. In a similar way with the best fitting model for lung cancer, different powers of age (up to 2) and different powers of year (up to 2) appear in the model. Several two-way interaction terms are also considered, such as between age and year, e.g. $\beta_{7,1} at_1$ and $\beta_{7,2} at_2$ referring two different estimates before and after 2006, respectively; between gender and age, e.g. $\beta_{11,g} a$; between gender and year, e.g. $\beta_{13,1,g} t_1$ and $\beta_{13,2,g} t_2$.

Observed and fitted incidence rates of malignant neoplasm of colorectal in England are illustrated in Fig 31-44 from age 47 onwards between 1981 and 2016. The incidence rates for males before age 60 are either levelled or slightly increasing (Fig 31). On the other hand, the relevant rates for females in the same age groups have increased in the most recent years although they had a slightly decreasing trend until 2006 (Fig 35). Furthermore, the incidence rates for males are gradually increasing after age 60 with some jumps right after 2006, and then drops in the most recent years in each region (Fig 32-33). The same age-specific incidence rates for females are either

flattened, e.g. South East, or increasing, e.g. London, before 2006, and then we observe similar jumps and drops right after 2006 (Fig 36-37). These jumps and drops after 2006 reflect the impact of screening programme on age-specific incidence rates between age 60 and 69 (Fig 32 for males and Fig 36 for females).

# 5 Model for incidence rates of malignant neoplasm of prostate

Malignant neoplasm of prostate is modelled from age 47 onwards. We regress the covariates on the location parameter of lognormal distribution as given below:

$$\mu_{a,t,r} = \beta_0 + \beta_1 a + \beta_2 a^2 + \beta_3 a^3 + \beta_4 t + \beta_5 t^2 + \beta_6 t^3 + \beta_{7,r} + \beta_8 at + \beta_9 at^2 + \beta_{10} at^3 \\ + \beta_{11} a^2 t + \beta_{12} a^3 t + \beta_{13,r} a,$$

where age, $a$, and year, $t$, are numerical variables with $a \in \{47, \ldots, 90\}$. In addition to different powers of age (up to 3) and different powers of year (up to 3), several two-way interaction terms between covariates, such as age and year, e.g. $\beta_8 at$; age and region, e.g. $\beta_{13,r} a$, appear in the best fitting model for prostate cancer.

Observed and fitted incidence rates of malignant neoplasm of prostate in England are shown in Fig 45-51 between 1981 and 2016. We observe a steep increase following a gradual increase after age 47 starting from 1980s in all regions (Fig 45-48). The changes in incidence rates between age 57 and 67 occur in sudden jumps starting in mid 1990s (Fig 45-46). After a peak in 2013, the sharp increase seems to decrease in the most recent years. A rise in the uptake of PSA testing is pointed out as an explanation of the increase.[4,5]

The figures show that different powers of age and year in the model allow us to capture the trend of observed rates accurately over years. Although an increasing trend is mostly observed, it is also possible to notice levelled age-specific rates in some regions, e.g. males aged 62 in South West, during most recent years (Fig 46). It is also noted that the rise in the prostate cancer incidence after age 60 has started to slow down in the recent years (Fig 46-48).

# 6 Model for incidence rates of malignant neoplasm of breast

Malignant neoplasm of breast is modelled from age 17 onwards as ONS and Cancer Research UK publications point out breast cancer as an important issue after age 15.[5–8] We regress the covariates on the location parameter of lognormal distribution as follows:

$$
\begin{aligned}
\mu_{a,t,r} = {} & \beta_0 + \beta_{1,1}a_1 + \beta_{1,2}a_2 + \beta_{2,1}t_1 + \beta_{2,2}t_2 + \beta_{3,r} + \beta_{4,1}a_1^2 + \beta_{4,2}a_2^2 + \beta_{5,1}a_1^3 + \beta_{5,2}a_2^3 \\
& + \beta_{6,1}t_1^2 + \beta_{6,2}t_2^2 + \beta_{7,1}a_1t_1 + \beta_{7,2}a_2t_1 + \beta_{7,3}a_1t_2 + \beta_{7,4}a_2t_2 + \beta_{8,1}a_1^2t_1 + \beta_{8,2}a_2^2t_1 \\
& + \beta_{8,3}a_1^2t_2 + \beta_{8,4}a_2^2t_2 + \beta_{9,1}a_1^3t_1 + \beta_{9,2}a_2^3t_1 + \beta_{9,3}a_1^3t_2 + \beta_{9,4}a_2^3t_2 \\
& + \beta_{10,1,r}t_1 + \beta_{10,2,r}t_2 + \beta_{11,1,r}a_1 + \beta_{11,2,r}a_2.
\end{aligned}
$$

Here, a break point is considered in year based on changepoint analysis, while another break point is taken into account in age due to epidemiological reasons in the literature.[9,10] Therefore, in the best fitting model, age, denoted by $a_1$ and $a_2$, is modelled as a numerical variable, with $a_1 < 50$ and $a_2 \geq 50$, and year, denoted by $t_1$ and $t_2$, is also numerical, where $t_1 \leq 1989$ and $t_2 > 1989$. Different powers of age (up to 3) and year (up to 2) are considered in addition to several interaction terms between covariates, such as region and year, e.g. $\beta_{10,1,r}t_1$ and $\beta_{10,2,r}t_2$ referring two different estimates before and after 1989. Regarding to interaction between age and year, note that there are four different estimates, e.g. $\beta_{7,1}a_1t_1$, $\beta_{7,2}a_2t_1$, $\beta_{7,3}a_1t_2$ and $\beta_{7,4}a_2t_2$, indicating the combination of age (before/after 50) and year (before/after 1989).

Observed and fitted incidence rates of malignant neoplasm of breast are shown in Fig 52-62 for all age groups in regions of England. Whilst there is a gradual increase after age 32 (Fig 54-59), there is a steep increase in incidence rates from age 52 to 62 right after 1985 (Fig 54-55). Whilst incidence of breast has a gradual increase at younger ages, there is a sudden change at age 52 starting from 1988 and going through early 1990s in each region (Fig 54). A similar sharp increase occurred in similar period from age 57 to 62, mostly with a peak in 1991 or 1992 (Fig 55). Although there is an increasing trend between ages 57 and 62 over years, levelled rates, e.g. in North East, or dropping rates, e.g. in West Midlands, at age 57 are notable in the recent years (Fig 55). Moreover, a steep increase starting in mid 1990s at age 67 has a peak around 2005 (Fig 56). This is followed by a gradual increase in successive years.

## References


[1] Killick R, Eckley IA. changepoint: An R Package for Changepoint Analysis. Journal of Statistical Software. 2014;58(3).

[2] Killick R, Fearnhead P, Eckley IA. Optimal Detection of Changepoints with a Linear Computational Cost. Journal of the American Statistical Association. 2012;107(500):1590–1598.

[3] Zhang NR, Siegmund DO. A Modified Bayes Information Criterion with Applications to the Analysis of Comparative Genomic Hybridization Data. Biometrics. 2007;63:22–32.

[4] ONS. Cancer Registration Statistics, England, 2008. Office for National Statistics; 2010.

[5] ONS. Cancer Registration Statistics, England, 2016. Office for National Statistics; 2018.

[6] ONS. Cancer Registration Statistics, England, 2013. Office for National Statistics; 2015.

[7] ONS. Cancer Registration Statistics, England, 2015. Office for National Statistics; 2017.

[8] CRUK. Cancer Incidence by age; 2018. Available from: https://www.cancerresearchuk.org/health-professional/cancer-statistics/incidence/age#heading-Two.

[9] Henderson BE, Ross R, Bernstein L. Estrogens as a cause of human cancer: the Richard and Hinda Rosenthal Foundation Award Lecture. Cancer Research. 1988;48):246–253.

[10] Bray F, McCarron P, Parkin DM. The Changing Global Patterns of Female Breast Cancer Incidence and Mortality. Breast Cancer Research. 2004;6(06).

**S1 Table. Estimated coefficients for the best fitting model for all-cancer rates.**

| Covariate | Parameter | Mean | SD | %2.5 | %97.5 |
|---|---|---|---|---|---|
| Intercept | $\beta_0$ | -5.7400 | 0.0013 | -5.7430 | -5.7370 |
| Age | $\beta_{1,\text{age}_1}$ | -2.8710 | 0.0093 | -2.8890 | -2.8530 |
| | $\beta_{1,\text{age}_2}$ | -2.4490 | 0.0075 | -2.4630 | -2.4340 |
| | $\beta_{1,\text{age}_3}$ | -2.0800 | 0.0062 | -2.0910 | -2.0670 |
| | $\beta_{1,\text{age}_4}$ | -1.8100 | 0.0058 | -1.8220 | -1.7990 |
| | $\beta_{1,\text{age}_5}$ | -1.5170 | 0.0053 | -1.5280 | -1.5070 |
| | $\beta_{1,\text{age}_6}$ | -1.0880 | 0.0046 | -1.0980 | -1.0800 |
| | $\beta_{1,\text{age}_7}$ | -0.5466 | 0.0040 | -0.5546 | -0.5389 |
| | $\beta_{1,\text{age}_8}$ | 0.0600 | 0.0038 | 0.0528 | 0.0674 |
| | $\beta_{1,\text{age}_9}$ | 0.6575 | 0.0033 | 0.6511 | 0.6644 |
| | $\beta_{1,\text{age}_{10}}$ | 1.1760 | 0.0029 | 1.1700 | 1.1820 |
| | $\beta_{1,\text{age}_{11}}$ | 1.6070 | 0.0029 | 1.6010 | 1.6120 |
| | $\beta_{1,\text{age}_{12}}$ | 1.9290 | 0.0029 | 1.9230 | 1.9340 |
| | $\beta_{1,\text{age}_{13}}$ | 2.1750 | 0.0029 | 2.1700 | 2.1810 |
| | $\beta_{1,\text{age}_{14}}$ | 2.3290 | 0.0031 | 2.3230 | 2.3350 |
| | $\beta_{1,\text{age}_{15}}$ | 2.4280 | 0.0032 | 2.4210 | 2.4340 |
| Year | $\beta_2$ | 0.0738 | 0.0012 | 0.0715 | 0.0762 |
| Gender | $\beta_3$ | 0.0208 | 0.0019 | 0.0171 | 0.0244 |
| Region | $\beta_{4,\text{region}_1}$ | 0.0498 | 0.0038 | 0.0426 | 0.0578 |
| | $\beta_{4,\text{region}_2}$ | 0.0384 | 0.0031 | 0.0319 | 0.0443 |
| | $\beta_{4,\text{region}_3}$ | 0.0173 | 0.0032 | 0.0112 | 0.0240 |
| | $\beta_{4,\text{region}_4}$ | -0.0377 | 0.0037 | -0.0449 | -0.0306 |
| | $\beta_{4,\text{region}_5}$ | -0.0072 | 0.0034 | -0.0141 | -0.0007 |
| | $\beta_{4,\text{region}_6}$ | -0.0575 | 0.0033 | -0.0641 | -0.0510 |
| | $\beta_{4,\text{region}_7}$ | -0.0212 | 0.0030 | -0.0269 | -0.0154 |
| | $\beta_{4,\text{region}_8}$ | -0.0054 | 0.0030 | -0.0112 | 0.0007 |
| | $\beta_{4,\text{region}_9}$ | 0.0235 | 0.0032 | 0.0171 | 0.0301 |
| Age:Year | $\beta_{5,\text{age}_1}$ | 0.0412 | 0.0066 | 0.0282 | 0.0539 |
| | $\beta_{5,\text{age}_2}$ | 0.0709 | 0.0053 | 0.0605 | 0.0809 |
| | $\beta_{5,\text{age}_3}$ | 0.1033 | 0.0045 | 0.0943 | 0.1117 |
| | $\beta_{5,\text{age}_4}$ | 0.0350 | 0.0039 | 0.0275 | 0.0426 |
| | $\beta_{5,\text{age}_5}$ | -0.0036 | 0.0034 | -0.0103 | 0.0029 |
| | $\beta_{5,\text{age}_6}$ | -0.0245 | 0.0029 | -0.0303 | -0.0190 |
| | $\beta_{5,\text{age}_7}$ | -0.0213 | 0.0026 | -0.0264 | -0.0161 |
| | $\beta_{5,\text{age}_8}$ | -0.0182 | 0.0024 | -0.0229 | -0.0135 |
| | $\beta_{5,\text{age}_9}$ | -0.0243 | 0.0023 | -0.0287 | -0.0199 |
| | $\beta_{5,\text{age}_{10}}$ | -0.0159 | 0.0022 | -0.0201 | -0.0115 |
| | $\beta_{5,\text{age}_{11}}$ | -0.0061 | 0.0021 | -0.0103 | -0.0019 |
| | $\beta_{5,\text{age}_{12}}$ | -0.0222 | 0.0021 | -0.0263 | -0.0181 |
| | $\beta_{5,\text{age}_{13}}$ | -0.0218 | 0.0021 | -0.0260 | -0.0176 |
| | $\beta_{5,\text{age}_{14}}$ | -0.0367 | 0.0023 | -0.0412 | -0.0322 |
| | $\beta_{5,\text{age}_{15}}$ | -0.0558 | 0.0023 | -0.0603 | -0.0514 |
| Age:Gender | $\beta_{6,\text{age}_1,\text{gender}_F}$ | -0.2054 | 0.0146 | -0.2317 | -0.1735 |
| | $\beta_{6,\text{age}_2,\text{gender}_F}$ | -0.0816 | 0.0105 | -0.1027 | -0.0624 |
| | $\beta_{6,\text{age}_3,\text{gender}_F}$ | 0.2462 | 0.0081 | 0.2304 | 0.2619 |
| | $\beta_{6,\text{age}_4,\text{gender}_F}$ | 0.5047 | 0.0074 | 0.4910 | 0.5201 |
| | $\beta_{6,\text{age}_5,\text{gender}_F}$ | 0.6833 | 0.0068 | 0.6714 | 0.6975 |
| | $\beta_{6,\text{age}_6,\text{gender}_F}$ | 0.7280 | 0.0061 | 0.7165 | 0.7407 |
| | $\beta_{6,\text{age}_7,\text{gender}_F}$ | 0.6270 | 0.0053 | 0.6170 | 0.6372 |
| | $\beta_{6,\text{age}_8,\text{gender}_F}$ | 0.3876 | 0.0049 | 0.3780 | 0.3974 |
| | $\beta_{6,\text{age}_9,\text{gender}_F}$ | 0.0594 | 0.0045 | 0.0504 | 0.0682 |
| | $\beta_{6,\text{age}_{10},\text{gender}_F}$ | -0.1796 | 0.0041 | -0.1873 | -0.1714 |
| | $\beta_{6,\text{age}_{11},\text{gender}_F}$ | -0.3995 | 0.0041 | -0.4076 | -0.3914 |
| | $\beta_{6,\text{age}_{12},\text{gender}_F}$ | -0.5333 | 0.0041 | -0.5411 | -0.5251 |
| | $\beta_{6,\text{age}_{13},\text{gender}_F}$ | -0.6034 | 0.0042 | -0.6118 | -0.5954 |
| | $\beta_{6,\text{age}_{14},\text{gender}_F}$ | -0.6172 | 0.0044 | -0.6261 | -0.6085 |
| | $\beta_{6,\text{age}_{15},\text{gender}_F}$ | -0.6163 | 0.0044 | -0.6248 | -0.6079 |
| Gender:Year | $\beta_{7,\text{gender}_F}$ | 0.0406 | 0.0013 | 0.0381 | 0.0432 |
| Age:Region | $\beta_{8,\text{age}_1,\text{region}_1}$ | -0.0623 | 0.0249 | -0.1118 | -0.0161 |
| | $\beta_{8,\text{age}_1,\text{region}_2}$ | -0.0289 | 0.0171 | -0.0617 | 0.0048 |
| | $\beta_{8,\text{age}_1,\text{region}_3}$ | 0.0002 | 0.0202 | -0.0405 | 0.0388 |
| | $\beta_{8,\text{age}_1,\text{region}_4}$ | -0.0302 | 0.0228 | -0.0756 | 0.0131 |
| | $\beta_{8,\text{age}_1,\text{region}_5}$ | -0.0184 | 0.0204 | -0.0600 | 0.0182 |
| | $\beta_{8,\text{age}_1,\text{region}_6}$ | 0.0145 | 0.0201 | -0.0272 | 0.0510 |
| | $\beta_{8,\text{age}_1,\text{region}_7}$ | 0.0466 | 0.0173 | 0.0137 | 0.0831 |
| | $\beta_{8,\text{age}_1,\text{region}_8}$ | 0.0289 | 0.0163 | -0.0042 | 0.0593 |
| | $\beta_{8,\text{age}_1,\text{region}_9}$ | 0.0495 | 0.0190 | 0.0133 | 0.0880 |
| | $\beta_{8,\text{age}_2,\text{region}_1}$ | -0.0803 | 0.0203 | -0.1226 | -0.0411 |
| | $\beta_{8,\text{age}_2,\text{region}_2}$ | -0.0073 | 0.0152 | -0.0382 | 0.0223 |
| | $\beta_{8,\text{age}_2,\text{region}_3}$ | -0.0541 | 0.0160 | -0.0846 | -0.0217 |
| | $\beta_{8,\text{age}_2,\text{region}_4}$ | -0.0181 | 0.0155 | -0.0472 | 0.0147 |
| | $\beta_{8,\text{age}_2,\text{region}_5}$ | -0.0158 | 0.0154 | -0.0461 | 0.0148 |
| | $\beta_{8,\text{age}_2,\text{region}_6}$ | 0.0170 | 0.0156 | -0.0126 | 0.0473 |
| | $\beta_{8,\text{age}_2,\text{region}_7}$ | -0.0031 | 0.0134 | -0.0306 | 0.0214 |
| | $\beta_{8,\text{age}_2,\text{region}_8}$ | 0.0917 | 0.0129 | 0.0660 | 0.1166 |
| | $\beta_{8,\text{age}_2,\text{region}_9}$ | 0.0700 | 0.0163 | 0.0393 | 0.1028 |
| | $\beta_{8,\text{age}_3,\text{region}_1}$ | -0.0259 | 0.0163 | -0.0597 | 0.0063 |
| | $\beta_{8,\text{age}_3,\text{region}_2}$ | -0.0157 | 0.0122 | -0.0396 | 0.0070 |
| | $\beta_{8,\text{age}_3,\text{region}_3}$ | 0.0138 | 0.0116 | -0.0089 | 0.0363 |
| | $\beta_{8,\text{age}_3,\text{region}_4}$ | 0.0039 | 0.0142 | -0.0222 | 0.0332 |
| | $\beta_{8,\text{age}_3,\text{region}_5}$ | 0.0005 | 0.0127 | -0.0249 | 0.0257 |
| | $\beta_{8,\text{age}_3,\text{region}_6}$ | 0.0021 | 0.0125 | -0.0224 | 0.0281 |
| | $\beta_{8,\text{age}_3,\text{region}_7}$ | -0.0954 | 0.0101 | -0.1156 | -0.0760 |
| | $\beta_{8,\text{age}_3,\text{region}_8}$ | 0.0331 | 0.0109 | 0.0117 | 0.0544 |
| | $\beta_{8,\text{age}_3,\text{region}_9}$ | 0.0835 | 0.0133 | 0.0569 | 0.1086 |
| | $\beta_{8,\text{age}_4,\text{region}_1}$ | -0.0420 | 0.0142 | -0.0706 | -0.0150 |
| | $\beta_{8,\text{age}_4,\text{region}_2}$ | -0.0038 | 0.0098 | -0.0227 | 0.0153 |
| | $\beta_{8,\text{age}_4,\text{region}_3}$ | 0.0218 | 0.0114 | -0.0006 | 0.0432 |
| | $\beta_{8,\text{age}_4,\text{region}_4}$ | 0.0102 | 0.0118 | -0.0122 | 0.0333 |
| | $\beta_{8,\text{age}_4,\text{region}_5}$ | -0.0026 | 0.0101 | -0.0223 | 0.0173 |
| | $\beta_{8,\text{age}_4,\text{region}_6}$ | -0.0030 | 0.0116 | -0.0258 | 0.0191 |
| | $\beta_{8,\text{age}_4,\text{region}_7}$ | -0.0700 | 0.0097 | -0.0888 | -0.0517 |
| | $\beta_{8,\text{age}_4,\text{region}_8}$ | 0.0248 | 0.0091 | 0.0074 | 0.0423 |
| | $\beta_{8,\text{age}_4,\text{region}_9}$ | 0.0646 | 0.0106 | 0.0432 | 0.0851 |
| | $\beta_{8,\text{age}_5,\text{region}_1}$ | -0.0045 | 0.0117 | -0.0268 | 0.0196 |
| | $\beta_{8,\text{age}_5,\text{region}_2}$ | -0.0120 | 0.0086 | -0.0292 | 0.0050 |
| | $\beta_{8,\text{age}_5,\text{region}_3}$ | 0.0107 | 0.0096 | -0.0084 | 0.0284 |
| | $\beta_{8,\text{age}_5,\text{region}_4}$ | -0.0058 | 0.0104 | -0.0272 | 0.0135 |
| | $\beta_{8,\text{age}_5,\text{region}_5}$ | 0.0023 | 0.0099 | -0.0173 | 0.0217 |
| | $\beta_{8,\text{age}_5,\text{region}_6}$ | -0.0200 | 0.0095 | -0.0379 | -0.0005 |
| | $\beta_{8,\text{age}_5,\text{region}_7}$ | -0.0305 | 0.0084 | -0.0466 | -0.0143 |
| | $\beta_{8,\text{age}_5,\text{region}_8}$ | 0.0223 | 0.0084 | 0.0056 | 0.0389 |
| | $\beta_{8,\text{age}_5,\text{region}_9}$ | 0.0374 | 0.0095 | 0.0198 | 0.0566 |
| | $\beta_{8,\text{age}_6,\text{region}_1}$ | -0.0143 | 0.0101 | -0.0340 | 0.0058 |
| | $\beta_{8,\text{age}_6,\text{region}_2}$ | 0.0060 | 0.0074 | -0.0080 | 0.0209 |
| | $\beta_{8,\text{age}_6,\text{region}_3}$ | 0.0044 | 0.0080 | -0.0110 | 0.0202 |

| Covariate | Parameter | Mean | SD | %2.5 | %97.5 | Covariate | Parameter | Mean | SD | %2.5 | %97.5 |
|---|---|---|---|---|---|---|---|---|---|---|---|
| Age:Region | $\beta_{8,\text{age}_6,\text{region}_4}$ | -0.0001 | 0.0089 | -0.0175 | 0.0175 | | $\beta_{8,\text{age}_{12},\text{region}_3}$ | 0.0060 | 0.0060 | -0.0055 | 0.0176 |
| | $\beta_{8,\text{age}_6,\text{region}_5}$ | 0.0034 | 0.0083 | -0.0122 | 0.0198 | | $\beta_{8,\text{age}_{12},\text{region}_4}$ | -0.0013 | 0.0066 | -0.0139 | 0.0117 |
| | $\beta_{8,\text{age}_6,\text{region}_6}$ | -0.0033 | 0.0082 | -0.0196 | 0.0122 | | $\beta_{8,\text{age}_{12},\text{region}_5}$ | 0.0080 | 0.0060 | -0.0041 | 0.0200 |
| | $\beta_{8,\text{age}_6,\text{region}_7}$ | -0.0012 | 0.0077 | -0.0165 | 0.0139 | | $\beta_{8,\text{age}_{12},\text{region}_6}$ | 0.0014 | 0.0060 | -0.0103 | 0.0134 |
| | $\beta_{8,\text{age}_6,\text{region}_8}$ | 0.0021 | 0.0071 | -0.0125 | 0.0158 | | $\beta_{8,\text{age}_{12},\text{region}_7}$ | 0.0182 | 0.0057 | 0.0072 | 0.0295 |
| | $\beta_{8,\text{age}_6,\text{region}_9}$ | 0.0029 | 0.0076 | -0.0122 | 0.0180 | | $\beta_{8,\text{age}_{12},\text{region}_8}$ | -0.0252 | 0.0057 | -0.0363 | -0.0138 |
| | $\beta_{8,\text{age}_7,\text{region}_1}$ | -0.0093 | 0.0090 | -0.0272 | 0.0084 | | $\beta_{8,\text{age}_{12},\text{region}_9}$ | -0.0550 | 0.0059 | -0.0667 | -0.0436 |
| | $\beta_{8,\text{age}_7,\text{region}_2}$ | 0.0112 | 0.0069 | -0.0023 | 0.0249 | | $\beta_{8,\text{age}_{13},\text{region}_1}$ | 0.0358 | 0.0066 | 0.0225 | 0.0487 |
| | $\beta_{8,\text{age}_7,\text{region}_3}$ | -0.0090 | 0.0073 | -0.0234 | 0.0055 | | $\beta_{8,\text{age}_{13},\text{region}_2}$ | -0.0005 | 0.0059 | -0.0122 | 0.0108 |
| | $\beta_{8,\text{age}_7,\text{region}_4}$ | 0.0056 | 0.0083 | -0.0109 | 0.0221 | | $\beta_{8,\text{age}_{13},\text{region}_3}$ | 0.0030 | 0.0063 | -0.0091 | 0.0149 |
| | $\beta_{8,\text{age}_7,\text{region}_5}$ | 0.0123 | 0.0074 | -0.0022 | 0.0268 | | $\beta_{8,\text{age}_{13},\text{region}_4}$ | 0.0103 | 0.0062 | -0.0024 | 0.0225 |
| | $\beta_{8,\text{age}_7,\text{region}_6}$ | -0.0152 | 0.0077 | -0.0300 | 0.0005 | | $\beta_{8,\text{age}_{13},\text{region}_5}$ | 0.0019 | 0.0061 | -0.0102 | 0.0138 |
| | $\beta_{8,\text{age}_7,\text{region}_7}$ | 0.0188 | 0.0068 | 0.0053 | 0.0321 | | $\beta_{8,\text{age}_{13},\text{region}_6}$ | 0.0134 | 0.0058 | 0.0022 | 0.0247 |
| | $\beta_{8,\text{age}_7,\text{region}_8}$ | -0.0099 | 0.0067 | -0.0235 | 0.0030 | | $\beta_{8,\text{age}_{13},\text{region}_7}$ | 0.0114 | 0.0058 | -0.0001 | 0.0227 |
| | $\beta_{8,\text{age}_7,\text{region}_9}$ | -0.0045 | 0.0075 | -0.0193 | 0.0100 | | $\beta_{8,\text{age}_{13},\text{region}_8}$ | -0.0323 | 0.0055 | -0.0430 | -0.0215 |
| | $\beta_{8,\text{age}_8,\text{region}_1}$ | 0.0174 | 0.0081 | 0.0020 | 0.0334 | | $\beta_{8,\text{age}_{13},\text{region}_9}$ | -0.0429 | 0.0060 | -0.0544 | -0.0311 |
| | $\beta_{8,\text{age}_8,\text{region}_2}$ | 0.0088 | 0.0061 | -0.0033 | 0.0208 | | $\beta_{8,\text{age}_{14},\text{region}_1}$ | 0.0260 | 0.0069 | 0.0124 | 0.0395 |
| | $\beta_{8,\text{age}_8,\text{region}_3}$ | 0.0048 | 0.0068 | -0.0090 | 0.0180 | | $\beta_{8,\text{age}_{14},\text{region}_2}$ | -0.0065 | 0.0060 | -0.0179 | 0.0054 |
| | $\beta_{8,\text{age}_8,\text{region}_4}$ | 0.0042 | 0.0071 | -0.0098 | 0.0178 | | $\beta_{8,\text{age}_{14},\text{region}_3}$ | -0.0028 | 0.0064 | -0.0151 | 0.0097 |
| | $\beta_{8,\text{age}_8,\text{region}_5}$ | 0.0116 | 0.0067 | -0.0017 | 0.0249 | | $\beta_{8,\text{age}_{14},\text{region}_4}$ | 0.0092 | 0.0067 | -0.0039 | 0.0224 |
| | $\beta_{8,\text{age}_8,\text{region}_6}$ | -0.0116 | 0.0068 | -0.0250 | 0.0019 | | $\beta_{8,\text{age}_{14},\text{region}_5}$ | -0.0125 | 0.0063 | -0.0249 | -0.0001 |
| | $\beta_{8,\text{age}_8,\text{region}_7}$ | 0.0109 | 0.0064 | -0.0018 | 0.0236 | | $\beta_{8,\text{age}_{14},\text{region}_6}$ | 0.0198 | 0.0063 | 0.0076 | 0.0323 |
| | $\beta_{8,\text{age}_8,\text{region}_8}$ | -0.0191 | 0.0063 | -0.0309 | -0.0063 | | $\beta_{8,\text{age}_{14},\text{region}_7}$ | 0.0160 | 0.0059 | 0.0050 | 0.0278 |
| | $\beta_{8,\text{age}_8,\text{region}_9}$ | -0.0269 | 0.0071 | -0.0408 | -0.0127 | | $\beta_{8,\text{age}_{14},\text{region}_8}$ | -0.0233 | 0.0059 | -0.0345 | -0.0118 |
| | $\beta_{8,\text{age}_9,\text{region}_1}$ | 0.0446 | 0.0069 | 0.0307 | 0.0579 | | $\beta_{8,\text{age}_{14},\text{region}_9}$ | -0.0259 | 0.0061 | -0.0381 | -0.0138 |
| | $\beta_{8,\text{age}_9,\text{region}_2}$ | 0.0190 | 0.0062 | 0.0071 | 0.0312 | | $\beta_{8,\text{age}_{15},\text{region}_1}$ | -0.0049 | 0.0076 | -0.0192 | 0.0102 |
| | $\beta_{8,\text{age}_9,\text{region}_3}$ | 0.0031 | 0.0065 | -0.0096 | 0.0158 | | $\beta_{8,\text{age}_{15},\text{region}_2}$ | -0.0215 | 0.0062 | -0.0341 | -0.0096 |
| | $\beta_{8,\text{age}_9,\text{region}_4}$ | -0.0056 | 0.0069 | -0.0193 | 0.0078 | | $\beta_{8,\text{age}_{15},\text{region}_3}$ | -0.0119 | 0.0063 | -0.0242 | 0.0006 |
| | $\beta_{8,\text{age}_9,\text{region}_5}$ | 0.0144 | 0.0068 | 0.0013 | 0.0279 | | $\beta_{8,\text{age}_{15},\text{region}_4}$ | 0.0214 | 0.0068 | 0.0081 | 0.0349 |
| | $\beta_{8,\text{age}_9,\text{region}_6}$ | -0.0200 | 0.0064 | -0.0322 | -0.0074 | | $\beta_{8,\text{age}_{15},\text{region}_5}$ | -0.0235 | 0.0066 | -0.0364 | -0.0104 |
| | $\beta_{8,\text{age}_9,\text{region}_7}$ | 0.0124 | 0.0062 | -0.0001 | 0.0241 | | $\beta_{8,\text{age}_{15},\text{region}_6}$ | 0.0159 | 0.0065 | 0.0033 | 0.0289 |
| | $\beta_{8,\text{age}_9,\text{region}_8}$ | -0.0257 | 0.0059 | -0.0374 | -0.0140 | | $\beta_{8,\text{age}_{15},\text{region}_7}$ | 0.0339 | 0.0061 | 0.0219 | 0.0458 |
| | $\beta_{8,\text{age}_9,\text{region}_9}$ | -0.0422 | 0.0065 | -0.0547 | -0.0291 | | $\beta_{8,\text{age}_{15},\text{region}_8}$ | -0.0066 | 0.0058 | -0.0178 | 0.0045 |
| | $\beta_{8,\text{age}_{10},\text{region}_1}$ | 0.0429 | 0.0068 | 0.0297 | 0.0565 | | $\beta_{8,\text{age}_{15},\text{region}_9}$ | -0.0029 | 0.0063 | -0.0150 | 0.0096 |
| | $\beta_{8,\text{age}_{10},\text{region}_2}$ | 0.0232 | 0.0060 | 0.0114 | 0.0352 | Region:Year | $\beta_{9,\text{region}_1}$ | 0.0080 | 0.0021 | 0.0037 | 0.0121 |
| | $\beta_{8,\text{age}_{10},\text{region}_3}$ | 0.0044 | 0.0061 | -0.0079 | 0.0159 | | $\beta_{9,\text{region}_2}$ | 0.0095 | 0.0018 | 0.0058 | 0.0130 |
| | $\beta_{8,\text{age}_{10},\text{region}_4}$ | -0.0050 | 0.0065 | -0.0179 | 0.0079 | | $\beta_{9,\text{region}_3}$ | -0.0004 | 0.0019 | -0.0042 | 0.0031 |
| | $\beta_{8,\text{age}_{10},\text{region}_5}$ | 0.0049 | 0.0060 | -0.0067 | 0.0168 | | $\beta_{9,\text{region}_4}$ | 0.0079 | 0.0021 | 0.0039 | 0.0121 |
| | $\beta_{8,\text{age}_{10},\text{region}_6}$ | -0.0065 | 0.0060 | -0.0186 | 0.0055 | | $\beta_{9,\text{region}_5}$ | -0.0140 | 0.0019 | -0.0177 | -0.0102 |
| | $\beta_{8,\text{age}_{10},\text{region}_7}$ | 0.0182 | 0.0060 | 0.0067 | 0.0297 | | $\beta_{9,\text{region}_6}$ | 0.0101 | 0.0019 | 0.0065 | 0.0139 |
| | $\beta_{8,\text{age}_{10},\text{region}_8}$ | -0.0301 | 0.0059 | -0.0417 | -0.0184 | | $\beta_{9,\text{region}_7}$ | -0.0182 | 0.0018 | -0.0218 | -0.0147 |
| | $\beta_{8,\text{age}_{10},\text{region}_9}$ | -0.0520 | 0.0062 | -0.0640 | -0.0401 | | $\beta_{9,\text{region}_8}$ | -0.0092 | 0.0018 | -0.0128 | -0.0057 |
| | $\beta_{8,\text{age}_{11},\text{region}_1}$ | 0.0408 | 0.0065 | 0.0280 | 0.0535 | | $\beta_{9,\text{region}_9}$ | 0.0064 | 0.0019 | 0.0027 | 0.0101 |
| | $\beta_{8,\text{age}_{11},\text{region}_2}$ | 0.0162 | 0.0060 | 0.0043 | 0.0280 | Gender:Region | $\beta_{10,\text{gender}_F,\text{region}_1}$ | -0.0316 | 0.0041 | -0.0398 | -0.0239 |
| | $\beta_{8,\text{age}_{11},\text{region}_3}$ | 0.0058 | 0.0063 | -0.0064 | 0.0185 | | $\beta_{10,\text{gender}_F,\text{region}_2}$ | -0.0165 | 0.0036 | -0.0234 | -0.0093 |
| | $\beta_{8,\text{age}_{11},\text{region}_4}$ | 0.0012 | 0.0063 | -0.0115 | 0.0134 | | $\beta_{10,\text{gender}_F,\text{region}_3}$ | -0.0022 | 0.0038 | -0.0097 | 0.0050 |
| | $\beta_{8,\text{age}_{11},\text{region}_5}$ | 0.0132 | 0.0060 | 0.0018 | 0.0254 | | $\beta_{10,\text{gender}_F,\text{region}_4}$ | 0.0240 | 0.0039 | 0.0161 | 0.0314 |
| | $\beta_{8,\text{age}_{11},\text{region}_6}$ | -0.0045 | 0.0060 | -0.0163 | 0.0074 | | $\beta_{10,\text{gender}_F,\text{region}_5}$ | -0.0043 | 0.0039 | -0.0120 | 0.0033 |
| | $\beta_{8,\text{age}_{11},\text{region}_7}$ | 0.0138 | 0.0058 | 0.0023 | 0.0256 | | $\beta_{10,\text{gender}_F,\text{region}_6}$ | 0.0230 | 0.0037 | 0.0154 | 0.0302 |
| | $\beta_{8,\text{age}_{11},\text{region}_8}$ | -0.0307 | 0.0057 | -0.0415 | -0.0197 | | $\beta_{10,\text{gender}_F,\text{region}_7}$ | -0.0342 | 0.0037 | -0.0415 | -0.0267 |
| | $\beta_{8,\text{age}_{11},\text{region}_9}$ | -0.0558 | 0.0060 | -0.0673 | -0.0439 | | $\beta_{10,\text{gender}_F,\text{region}_8}$ | 0.0178 | 0.0036 | 0.0107 | 0.0248 |
| | $\beta_{8,\text{age}_{12},\text{region}_1}$ | 0.0361 | 0.0066 | 0.0229 | 0.0492 | | $\beta_{10,\text{gender}_F,\text{region}_9}$ | 0.0242 | 0.0037 | 0.0170 | 0.0312 |
| | $\beta_{8,\text{age}_{12},\text{region}_2}$ | 0.0118 | 0.0061 | -0.0002 | 0.0240 | | $\sigma^2$ | 0.0022 | 0.0001 | 0.0021 | 0.0023 |

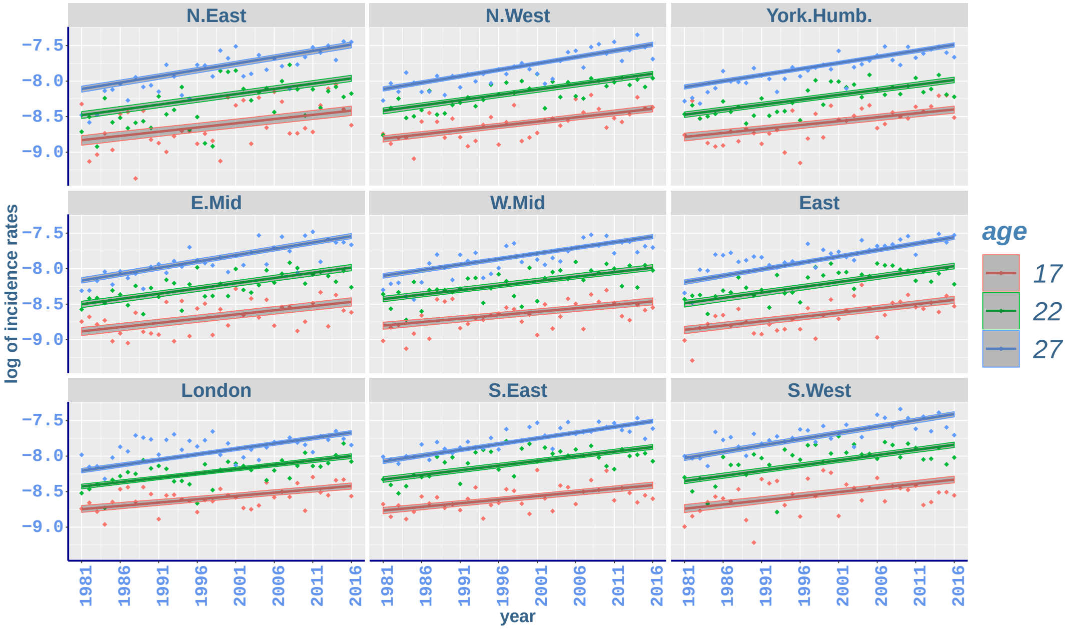


**S1 Fig. All-cancer incidence (excluding carcinoma in situ and skin cancer) for males, ages 17-27: observed rates (dots), fitted rates (lines) on log-scale with 95% credible intervals for the fitted rates.**

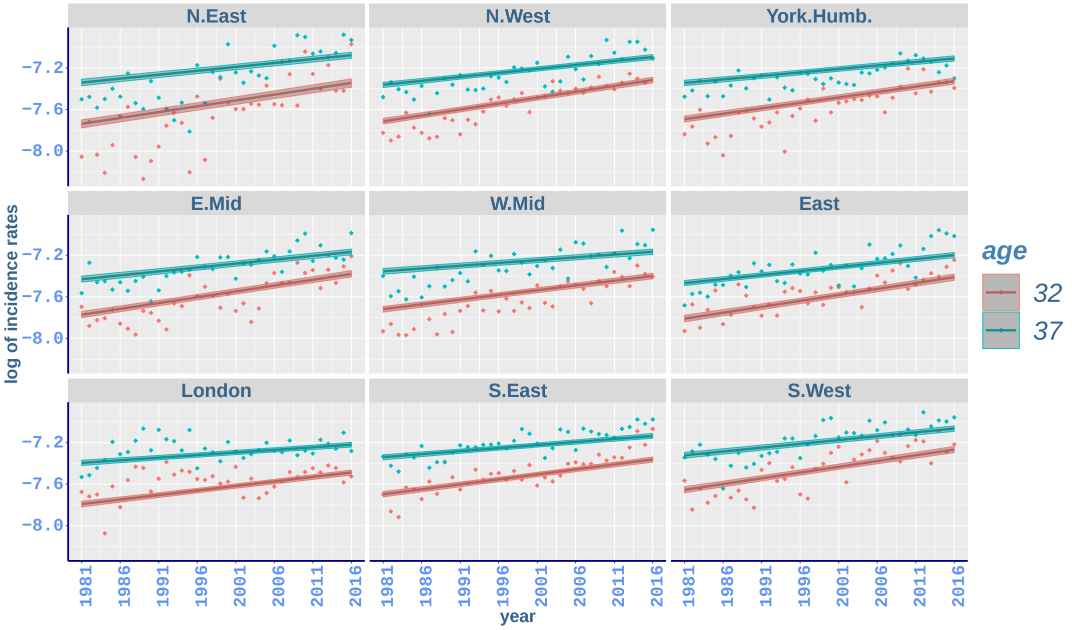


**S2 Fig. All-cancer incidence (excluding carcinoma in situ and skin cancer) for males, ages 32-37: observed rates (dots), fitted rates (lines) on log-scale with 95% credible intervals for the fitted rates.**

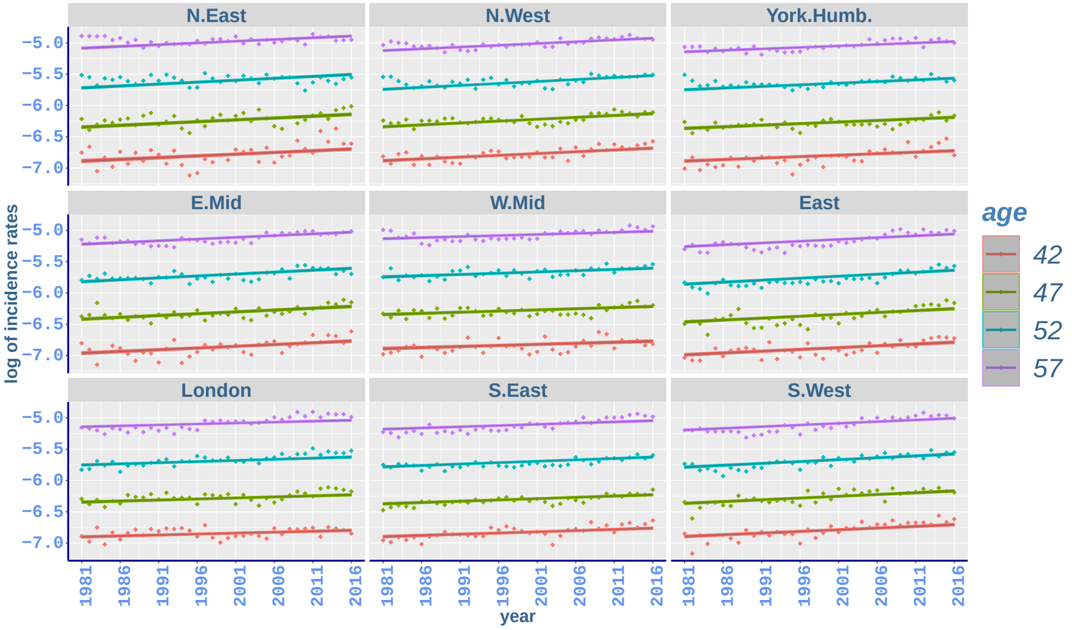


**S3 Fig. All-cancer incidence (excluding carcinoma in situ and skin cancer) for males, ages 42-57: observed rates (dots), fitted rates (lines) on log-scale with 95% credible intervals for the fitted rates.**

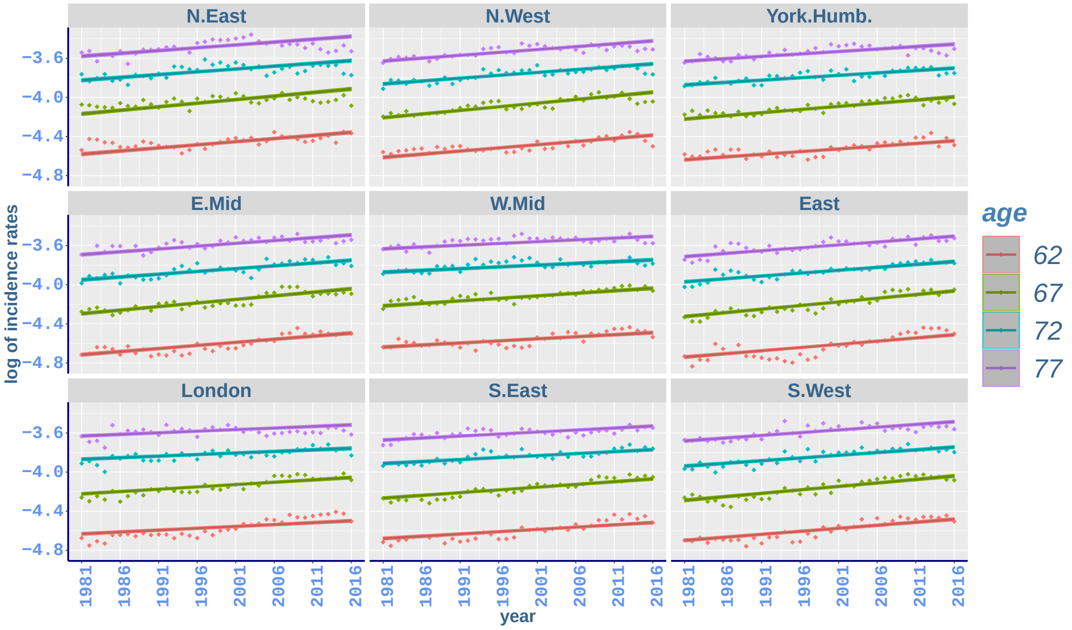


**S4 Fig. All-cancer incidence (excluding carcinoma in situ and skin cancer) for males, ages 62-77: observed rates (dots), fitted rates (lines) on log-scale with 95% credible intervals for the fitted rates.**

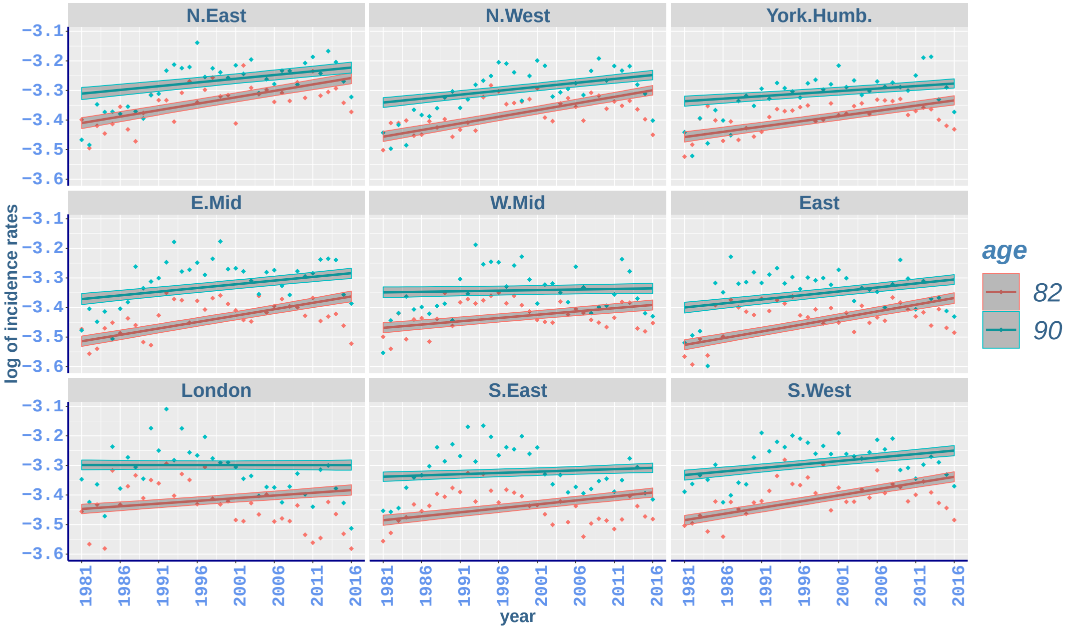


**S5 Fig. All-cancer incidence (excluding carcinoma in situ and skin cancer) for males, ages 82-90: observed rates (dots), fitted rates (lines) on log-scale with 95% credible intervals for the fitted rates.**

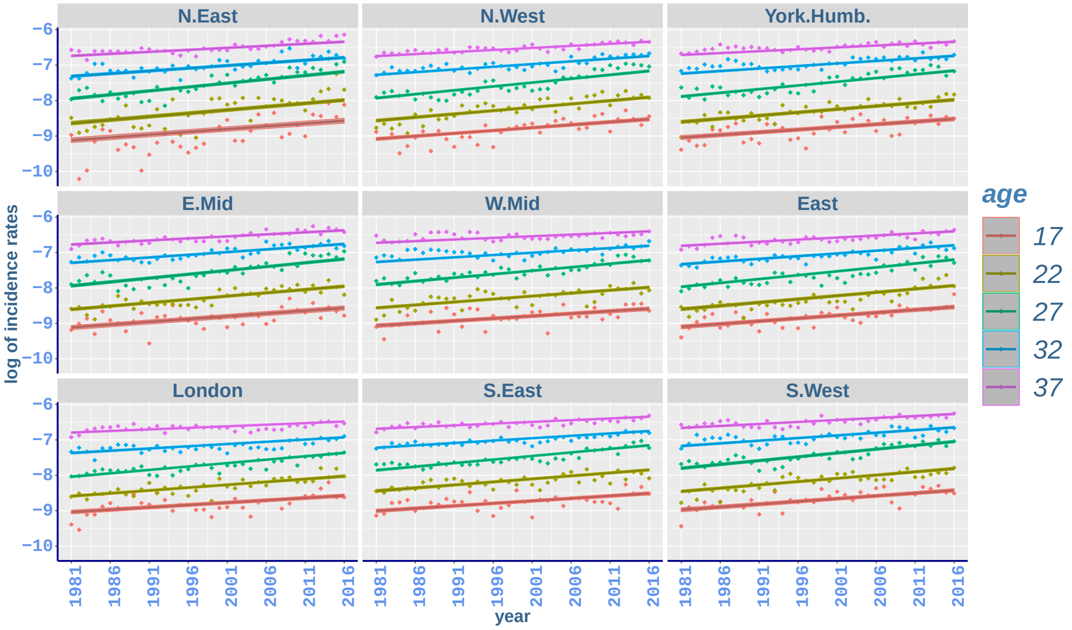


**S6 Fig. All-cancer incidence (excluding carcinoma in situ and skin cancer) for females, ages 17-37: observed rates (dots), fitted rates (lines) on log-scale with 95% credible intervals for the fitted rates.**

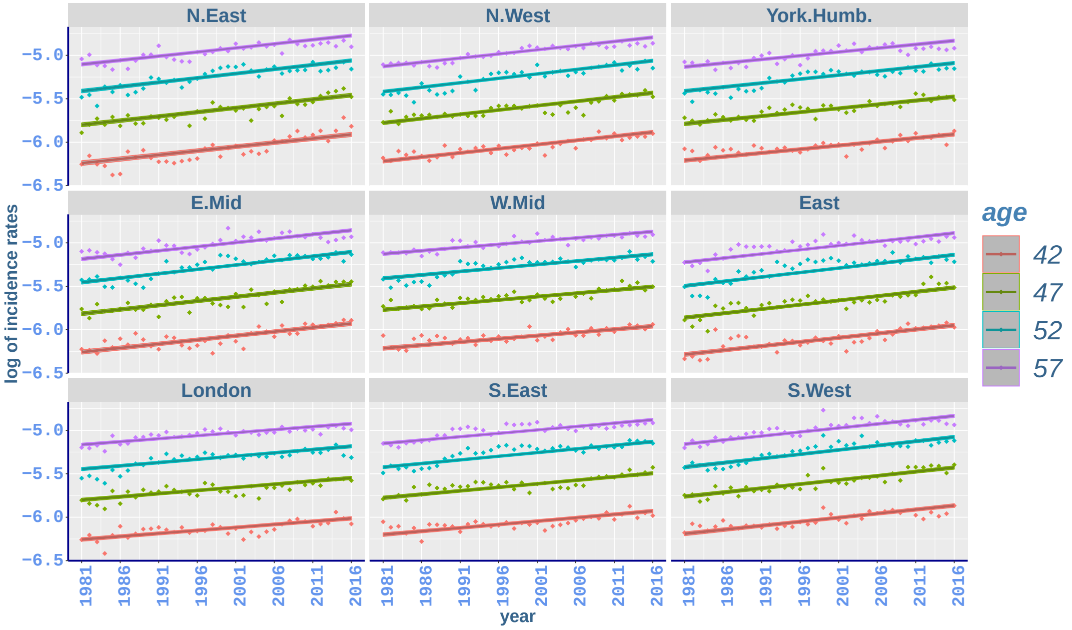


**S7 Fig. All-cancer incidence (excluding carcinoma in situ and skin cancer) for females, ages 42-57: observed rates (dots), fitted rates (lines) on log-scale with 95% credible intervals for the fitted rates.**

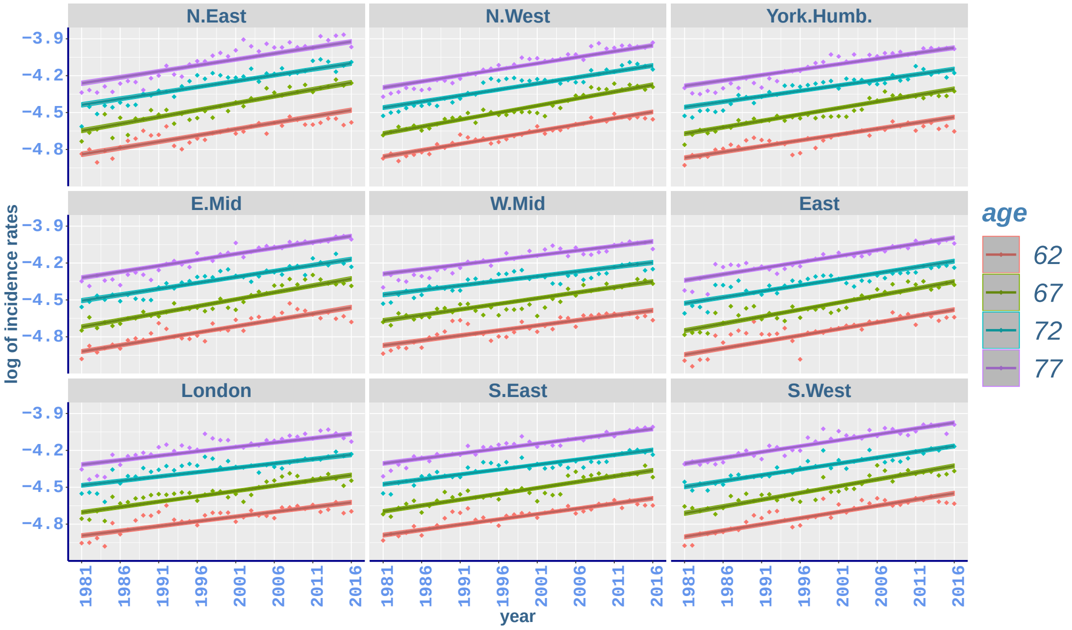


**S8 Fig. All-cancer incidence (excluding carcinoma in situ and skin cancer) for females, ages 62-77: observed rates (dots), fitted rates (lines) on log-scale with 95% credible intervals for the fitted rates.**

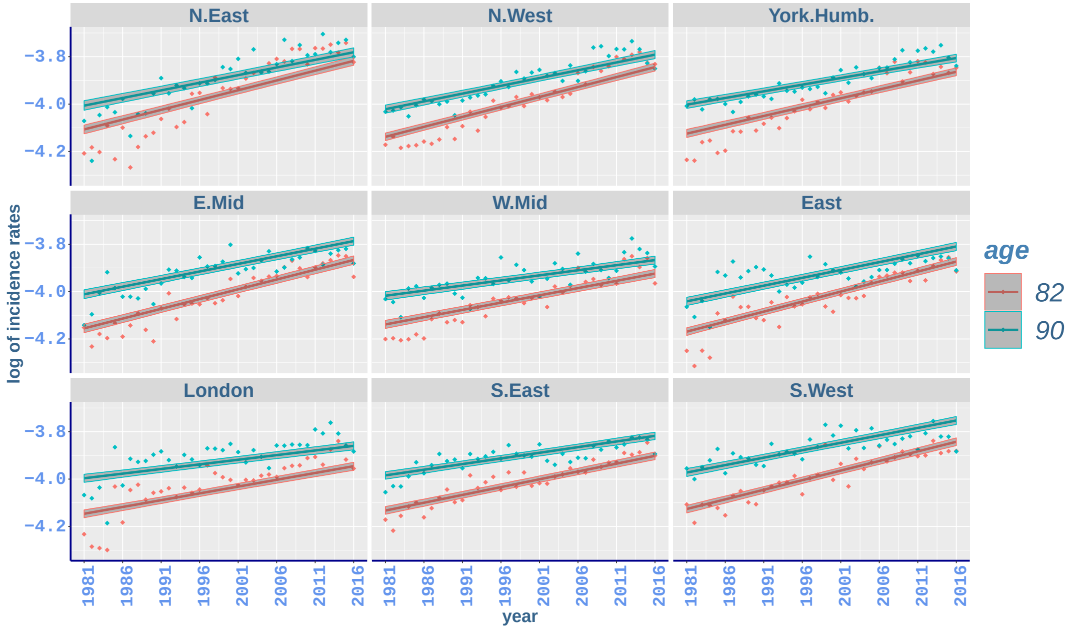


**S9 Fig. All-cancer incidence (excluding carcinoma in situ and skin cancer) for females, ages 82-90: observed rates (dots), fitted rates (lines) on log-scale with 95% credible intervals for the fitted rates.**

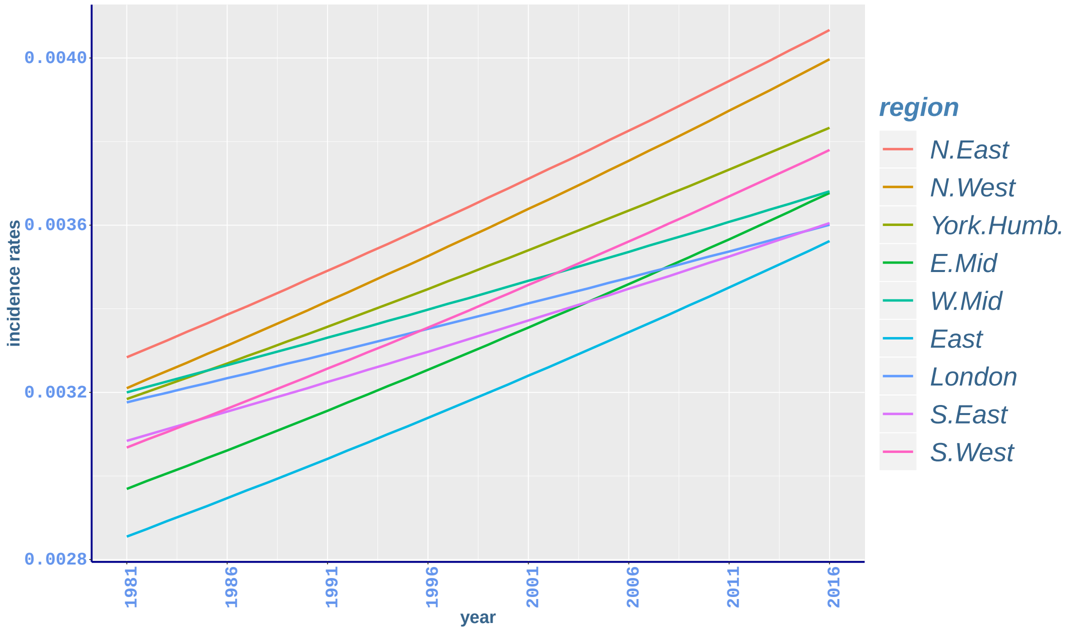


**S10 Fig. All-cancer incidence (excluding carcinoma in situ and skin cancer) for males, age 52: fitted rates.**

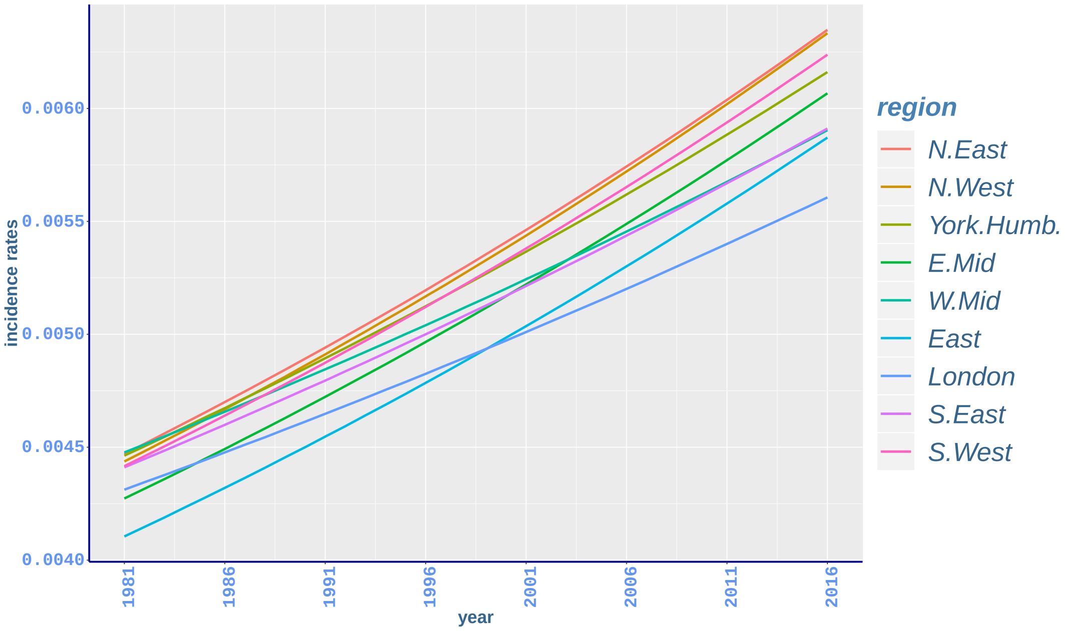


**S11 Fig. All-cancer incidence (excluding carcinoma in situ and skin cancer) for females, age 52: fitted rates.**

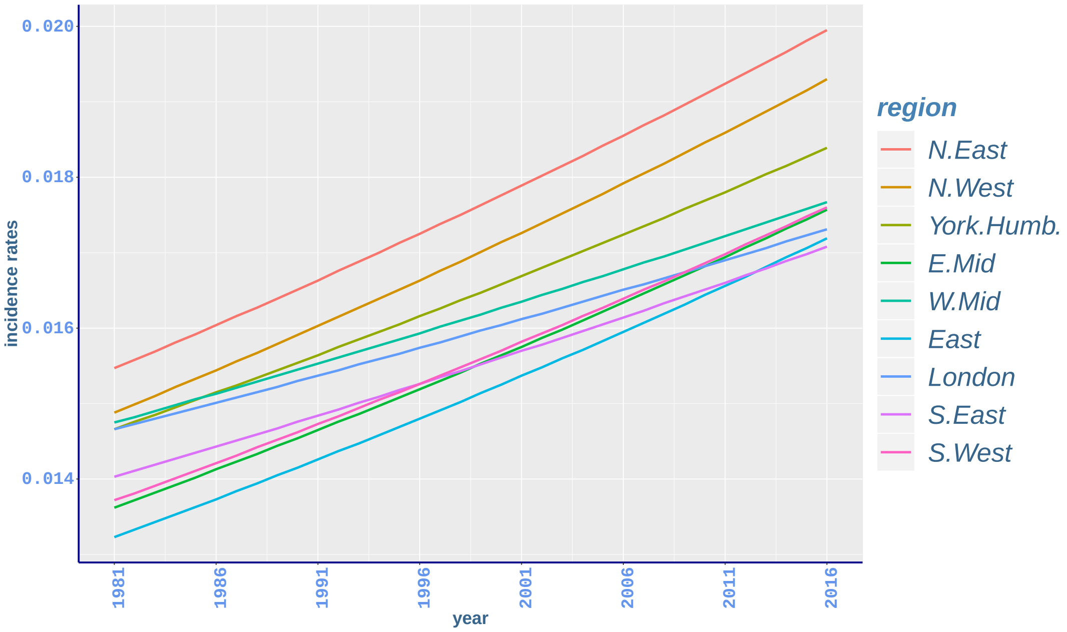


**S12 Fig. All-cancer incidence (excluding carcinoma in situ and skin cancer) for males, age 67: fitted rates.**

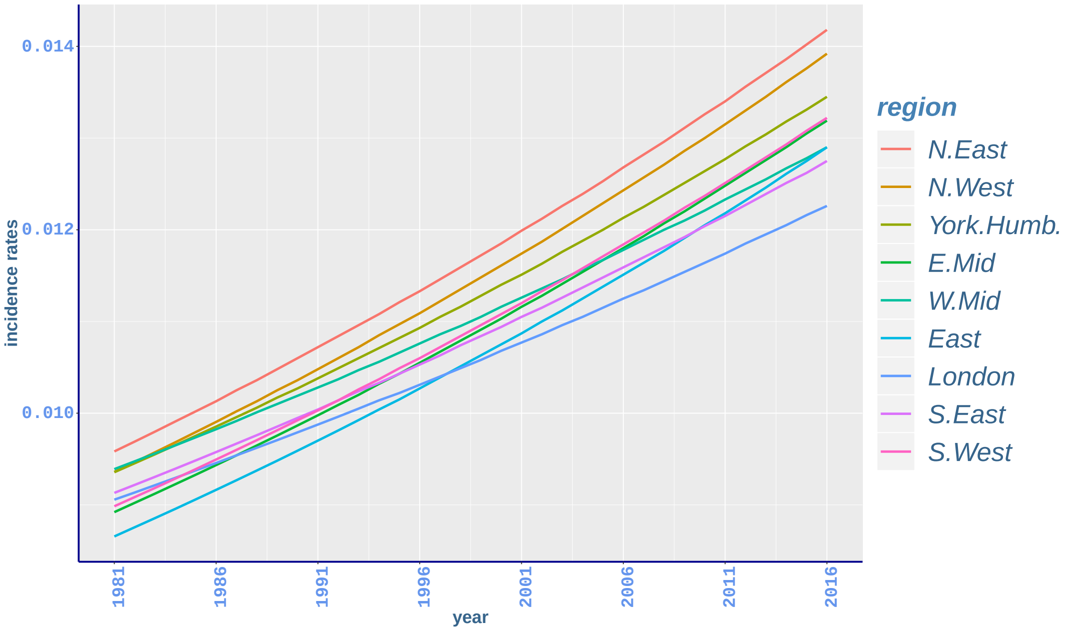


**S13 Fig. All-cancer incidence (excluding carcinoma in situ and skin cancer) for females, age 67: fitted rates.**

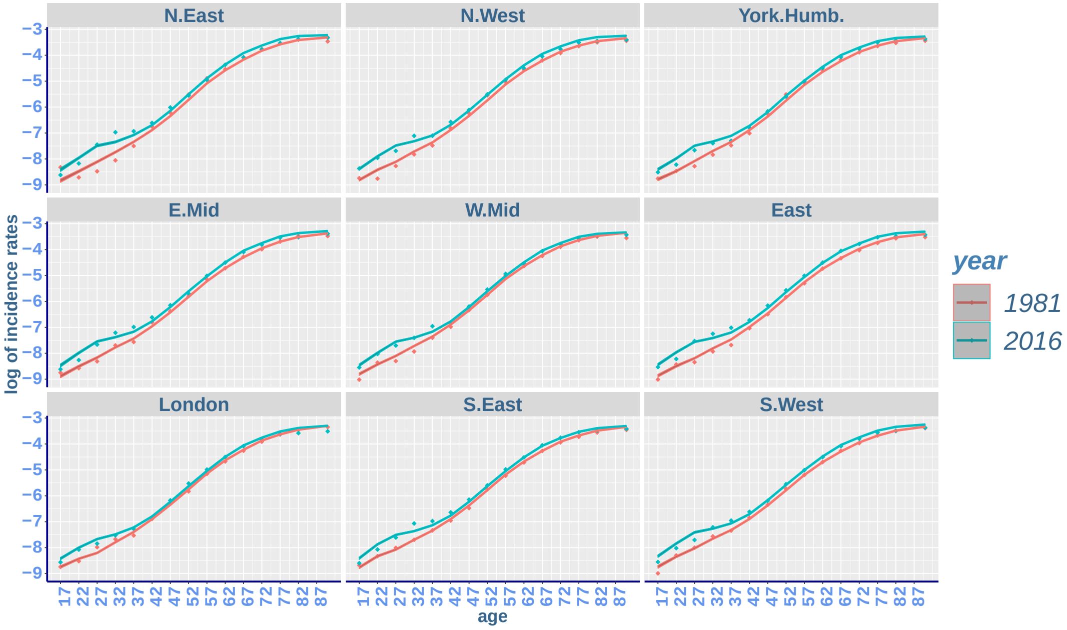


**S14 Fig. All-cancer incidence (excluding carcinoma in situ and skin cancer) for males, year 1981 and 2016: observed rates (dots), fitted rates (lines) on log-scale, with 95% credible intervals for the fitted rates.**

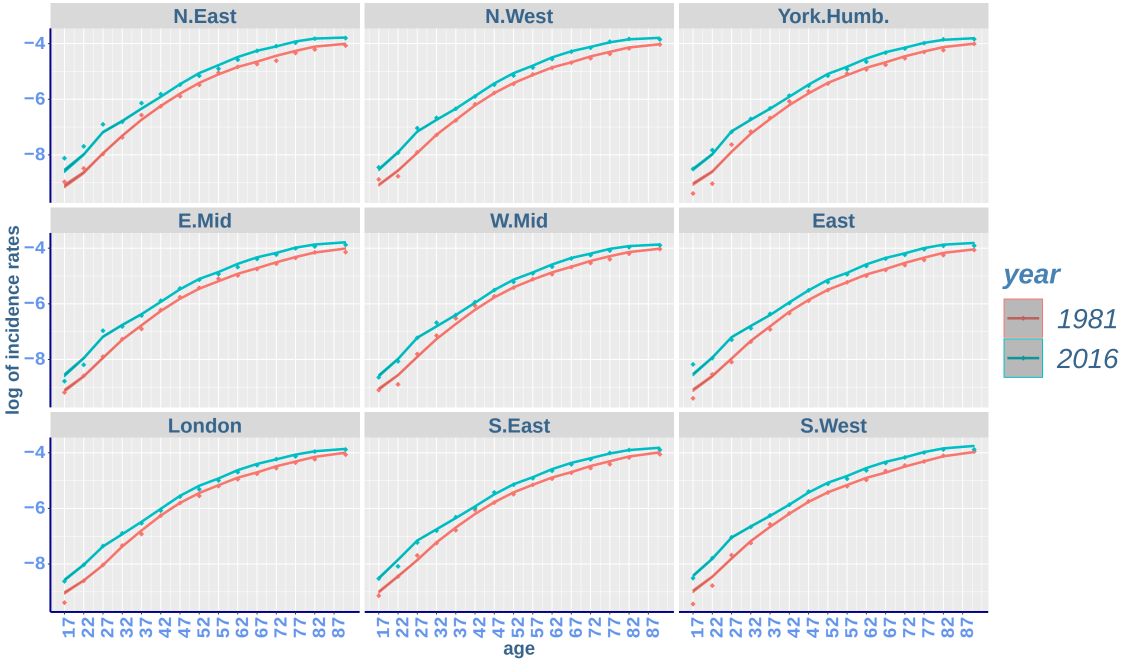


**S15 Fig. All-cancer incidence (excluding carcinoma in situ and skin cancer) for females, year 1981 and 2016: observed rates (dots), fitted rates (lines) on log-scale, with 95% credible intervals for the fitted rates.**

**S2 Table. Estimated coefficients for the best fitting model for tranchea, bronchus and lung cancer rates.**

| Covariate | Parameter | Mean | SD | %2.5 | %97.5 | Covariate | Parameter | Mean | SD | %2.5 | %97.5 |
|---|---|---|---|---|---|---|---|---|---|---|---|
| Intercept | $\beta_0$ | -5.7240 | 0.0032 | -5.7300 | -5.7170 | | $\beta_{14,\text{region}_4}$ | -0.0021 | 0.0054 | -0.0127 | 0.0081 |
| Age | $\beta_1$ | 1.1250 | 0.0032 | 1.1180 | 1.1310 | | $\beta_{14,\text{region}_5}$ | -0.0233 | 0.0050 | -0.0331 | -0.0138 |
| Age$^2$ | $\beta_2$ | -0.4380 | 0.0024 | -0.4428 | -0.4334 | | $\beta_{14,\text{region}_6}$ | 0.0010 | 0.0053 | -0.0091 | 0.0115 |
| Year | $\beta_3$ | -0.2615 | 0.0024 | -0.2662 | -0.2569 | | $\beta_{14,\text{region}_7}$ | 0.0318 | 0.0049 | 0.0221 | 0.0413 |
| Year$^2$ | $\beta_4$ | 0.0183 | 0.0017 | 0.0151 | 0.0216 | | $\beta_{14,\text{region}_8}$ | 0.0169 | 0.0045 | 0.0080 | 0.0261 |
| Gender | $\beta_5$ | -0.7296 | 0.0040 | -0.7374 | -0.7219 | | $\beta_{14,\text{region}_9}$ | 0.0375 | 0.0051 | 0.0277 | 0.0474 |
| Region | $\beta_{6,\text{region}_1}$ | 0.3449 | 0.0088 | 0.3272 | 0.3622 | Year:Region | $\beta_{15,\text{region}_1}$ | 0.0302 | 0.0045 | 0.0213 | 0.0391 |
| | $\beta_{6,\text{region}_2}$ | 0.1808 | 0.0076 | 0.1659 | 0.1959 | | $\beta_{15,\text{region}_2}$ | 0.0265 | 0.0038 | 0.0191 | 0.0340 |
| | $\beta_{6,\text{region}_3}$ | 0.1332 | 0.0081 | 0.1169 | 0.1485 | | $\beta_{15,\text{region}_3}$ | 0.0462 | 0.0039 | 0.0383 | 0.0539 |
| | $\beta_{6,\text{region}_4}$ | -0.0448 | 0.0084 | -0.0617 | -0.0281 | | $\beta_{15,\text{region}_4}$ | 0.0182 | 0.0044 | 0.0095 | 0.0269 |
| | $\beta_{6,\text{region}_5}$ | 0.0210 | 0.0080 | 0.0058 | 0.0367 | | $\beta_{15,\text{region}_5}$ | -0.0053 | 0.0042 | -0.0135 | 0.0029 |
| | $\beta_{6,\text{region}_6}$ | -0.1930 | 0.0087 | -0.2096 | -0.1755 | | $\beta_{15,\text{region}_6}$ | -0.0130 | 0.0042 | -0.0211 | -0.0046 |
| | $\beta_{6,\text{region}_7}$ | 0.0163 | 0.0078 | 0.0010 | 0.0316 | | $\beta_{15,\text{region}_7}$ | -0.0579 | 0.0041 | -0.0658 | -0.0497 |
| | $\beta_{6,\text{region}_8}$ | -0.2051 | 0.0083 | -0.2210 | -0.1882 | | $\beta_{15,\text{region}_8}$ | -0.0463 | 0.0040 | -0.0539 | -0.0387 |
| | $\beta_{6,\text{region}_9}$ | -0.2534 | 0.0082 | -0.2696 | -0.2377 | | $\beta_{15,\text{region}_9}$ | 0.0014 | 0.0041 | -0.0067 | 0.0092 |
| Age:Year | $\beta_7$ | 0.0952 | 0.0018 | 0.0917 | 0.0988 | | $\beta_{16,\text{region}_1}$ | -0.0358 | 0.0051 | -0.0459 | -0.0256 |
| Age$^2$ : Year | $\beta_8$ | 0.0218 | 0.0018 | 0.0182 | 0.0254 | Year$^2$ : Region | $\beta_{16,\text{region}_2}$ | -0.0039 | 0.0043 | -0.0124 | 0.0044 |
| Age : Year$^2$ | $\beta_9$ | -0.0130 | 0.0019 | -0.0167 | -0.0091 | | $\beta_{16,\text{region}_3}$ | -0.0146 | 0.0046 | -0.0236 | -0.0056 |
| Age:Gender | $\beta_{10,\text{gender}_F}$ | -0.2784 | 0.0038 | -0.2856 | -0.2708 | | $\beta_{16,\text{region}_4}$ | -0.0038 | 0.0049 | -0.0136 | 0.0058 |
| Age$^2$ : Gender | $\beta_{11,\text{gender}_F}$ | -0.0195 | 0.0037 | -0.0266 | -0.0117 | | $\beta_{16,\text{region}_5}$ | 0.0009 | 0.0046 | -0.0082 | 0.0094 |
| Year:Gender | $\beta_{12,\text{gender}_F}$ | 0.3622 | 0.0030 | 0.3563 | 0.3679 | | $\beta_{16,\text{region}_6}$ | 0.0215 | 0.0048 | 0.0121 | 0.0307 |
| Age:Region | $\beta_{13,\text{region}_1}$ | -0.0127 | 0.0058 | -0.0243 | -0.0013 | | $\beta_{16,\text{region}_7}$ | -0.0107 | 0.0044 | -0.0192 | -0.0019 |
| | $\beta_{13,\text{region}_2}$ | -0.0478 | 0.0046 | -0.0566 | -0.0386 | | $\beta_{16,\text{region}_8}$ | 0.0240 | 0.0044 | 0.0155 | 0.0326 |
| | $\beta_{13,\text{region}_3}$ | -0.0141 | 0.0051 | -0.0239 | -0.0044 | | $\beta_{16,\text{region}_9}$ | 0.0225 | 0.0048 | 0.0134 | 0.0320 |
| | $\beta_{13,\text{region}_4}$ | -0.0046 | 0.0055 | -0.0159 | 0.0061 | Gender:Region | $\beta_{17,\text{gender}_F,\text{region}_1}$ | 0.1078 | 0.0091 | 0.0908 | 0.1264 |
| | $\beta_{13,\text{region}_5}$ | -0.0227 | 0.0053 | -0.0333 | -0.0123 | | $\beta_{17,\text{gender}_F,\text{region}_2}$ | 0.0518 | 0.0078 | 0.0364 | 0.0669 |
| | $\beta_{13,\text{region}_6}$ | 0.0479 | 0.0053 | 0.0378 | 0.0587 | | $\beta_{17,\text{gender}_F,\text{region}_3}$ | 0.0307 | 0.0085 | 0.0140 | 0.0469 |
| | $\beta_{13,\text{region}_7}$ | 0.0209 | 0.0047 | 0.0117 | 0.0301 | | $\beta_{17,\text{gender}_F,\text{region}_4}$ | -0.0441 | 0.0089 | -0.0616 | -0.0266 |
| | $\beta_{13,\text{region}_8}$ | 0.0368 | 0.0045 | 0.0281 | 0.0455 | | $\beta_{17,\text{gender}_F,\text{region}_5}$ | -0.1258 | 0.0088 | -0.1434 | -0.1082 |
| | $\beta_{13,\text{region}_9}$ | -0.0037 | 0.0051 | -0.0137 | 0.0059 | | $\beta_{17,\text{gender}_F,\text{region}_6}$ | -0.0125 | 0.0089 | -0.0299 | 0.0046 |
| Age$^2$ : Region | $\beta_{14,\text{region}_1}$ | -0.0359 | 0.0058 | -0.0473 | -0.0250 | | $\beta_{17,\text{gender}_F,\text{region}_7}$ | 0.0154 | 0.0081 | -0.0004 | 0.0312 |
| | $\beta_{14,\text{region}_2}$ | -0.0082 | 0.0045 | -0.0171 | 0.0005 | | $\beta_{17,\text{gender}_F,\text{region}_8}$ | 0.0043 | 0.0079 | -0.0111 | 0.0197 |
| | $\beta_{14,\text{region}_3}$ | -0.0176 | 0.0050 | -0.0269 | -0.0075 | | $\beta_{17,\text{gender}_F,\text{region}_9}$ | -0.0276 | 0.0088 | -0.0451 | -0.0107 |
| | | | | | | | $\sigma^2$ | 0.0062 | 0.0002 | 0.0058 | 0.0067 |

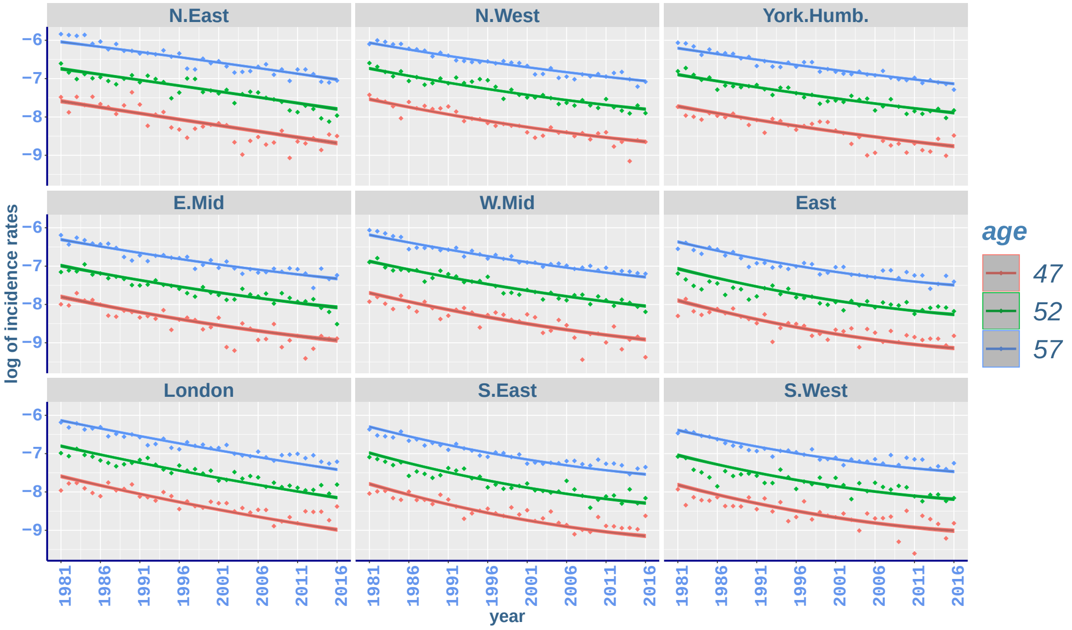


**S16 Fig. Tranchea, bronchus and lung cancer incidence for males, ages 47-57: observed rates (dots), fitted rates (lines) on log-scale, with 95% credible intervals for the fitted rates.**

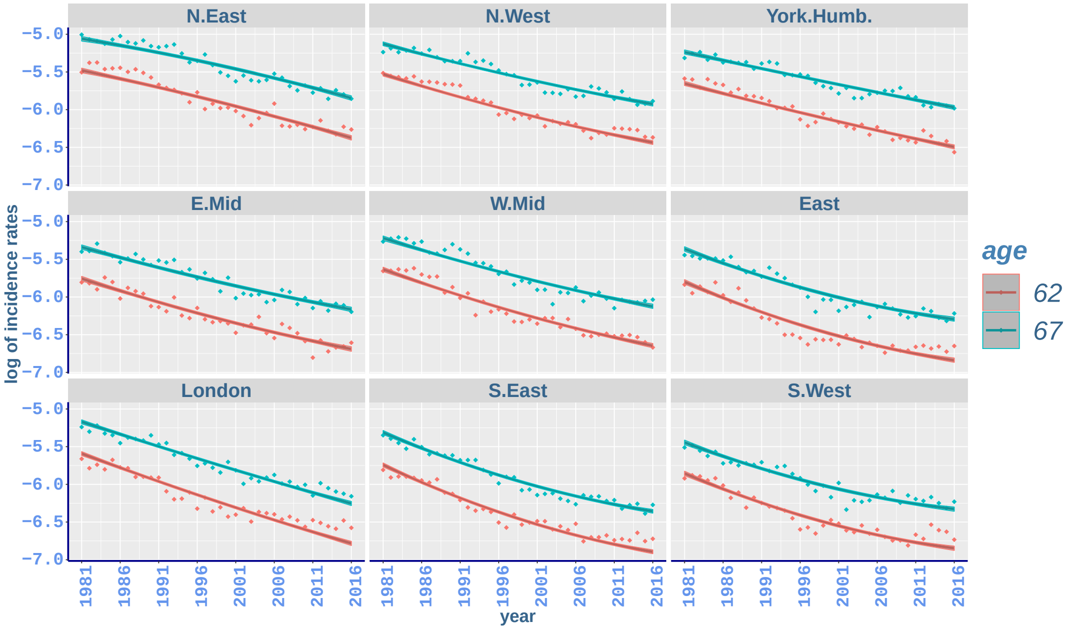


**S17 Fig. Tranchea, bronchus and lung cancer incidence for males, ages 62-67: observed rates (dots), fitted rates (lines) on log-scale, with 95% credible intervals for the fitted rates.**

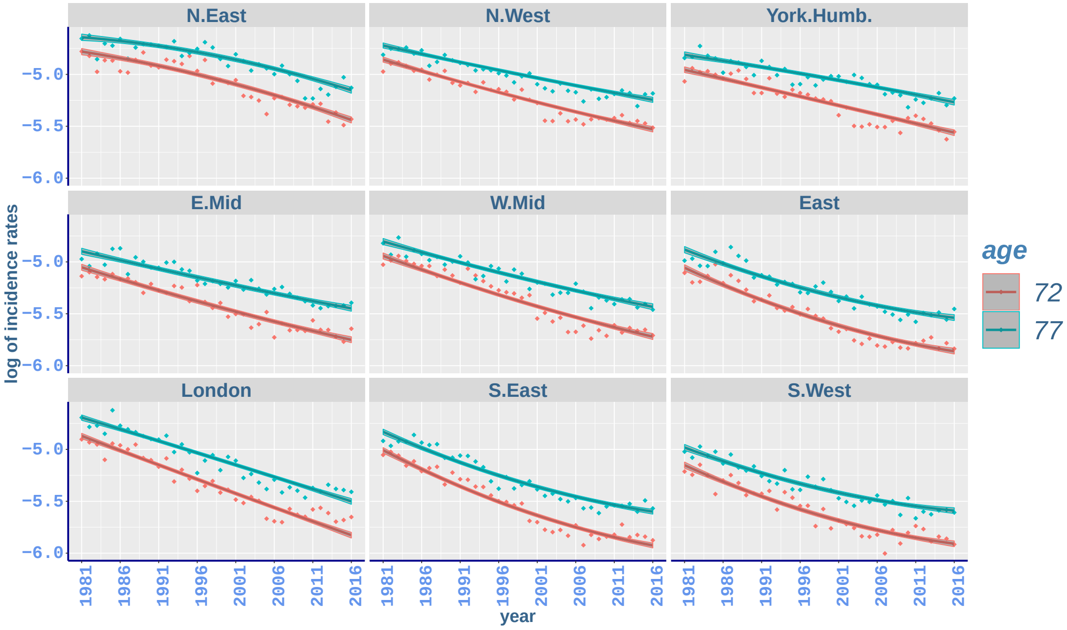


**S18 Fig. Tranchea, bronchus and lung cancer incidence for males, ages 72-77: observed rates (dots), fitted rates (lines) on log-scale, with 95% credible intervals for the fitted rates.**

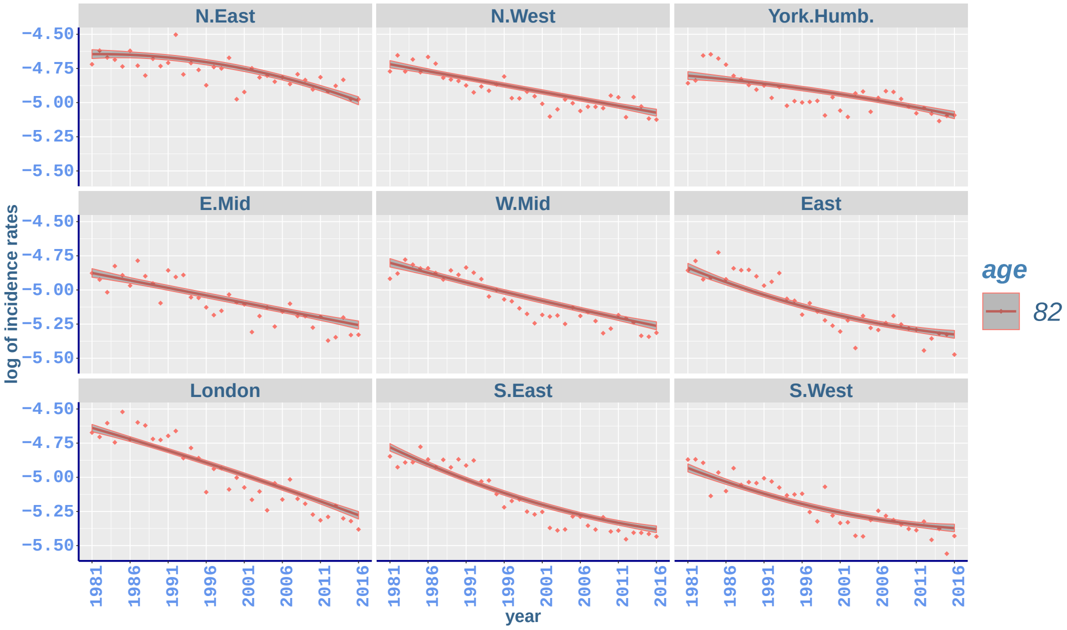


**S19 Fig. Tranchea, bronchus and lung cancer incidence for males, age 82: observed rates (dots), fitted rates (lines) on log-scale, with 95% credible intervals for the fitted rates.**

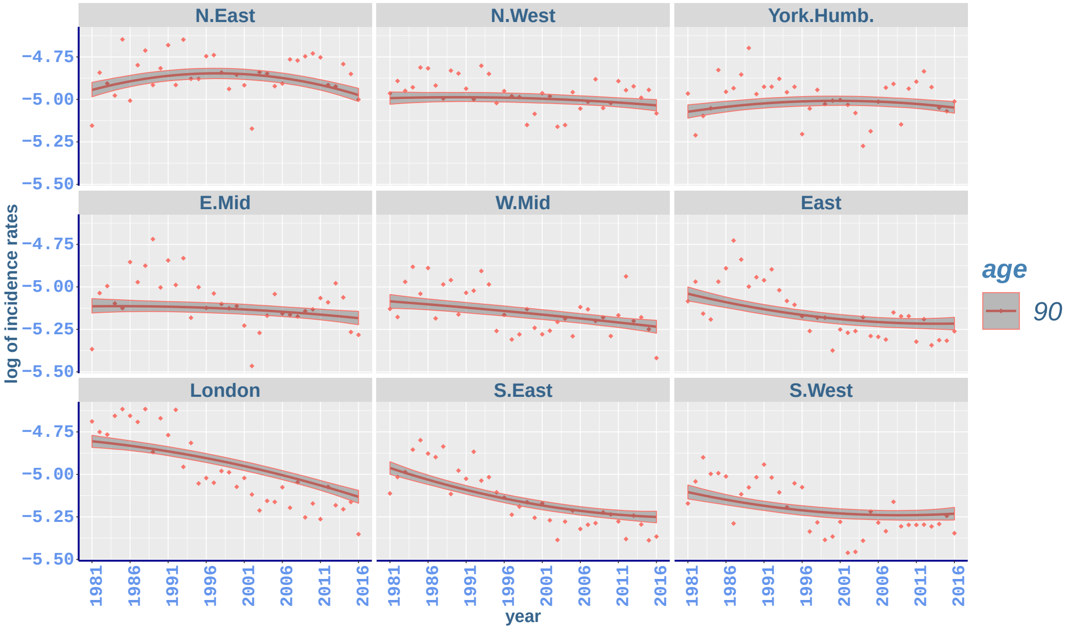


**S20 Fig. Tranchea, bronchus and lung cancer incidence for males, age 90: observed rates (dots), fitted rates (lines) on log-scale, with 95% credible intervals for the fitted rates.**

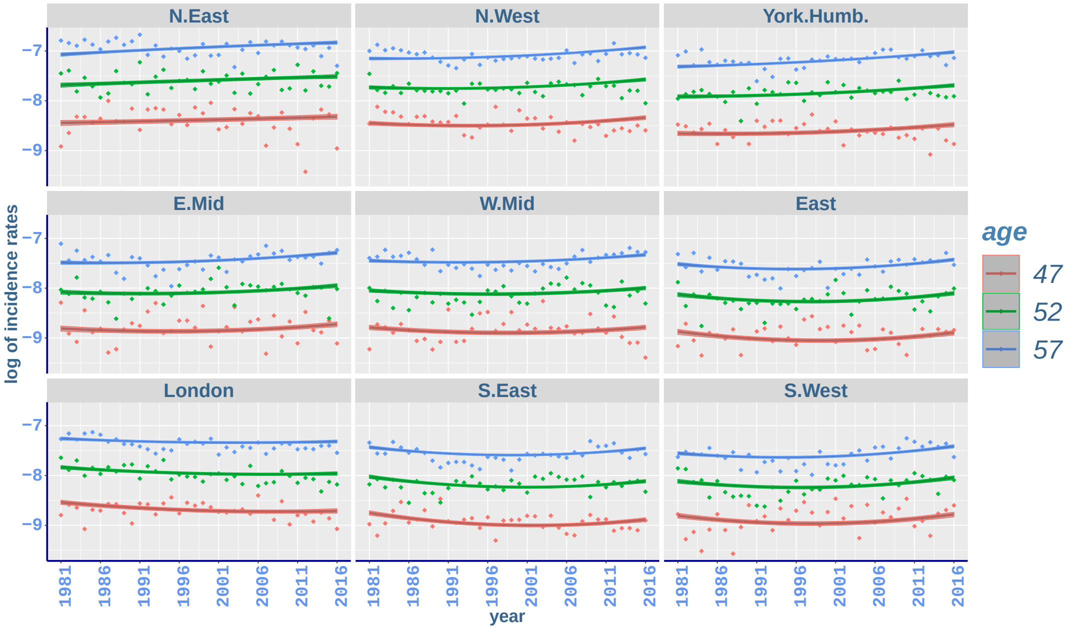


**S21 Fig. Tranchea, bronchus and lung cancer incidence for females, ages 47-57: observed rates (dots), fitted rates (lines) on log-scale, with 95% credible intervals for the fitted rates.**

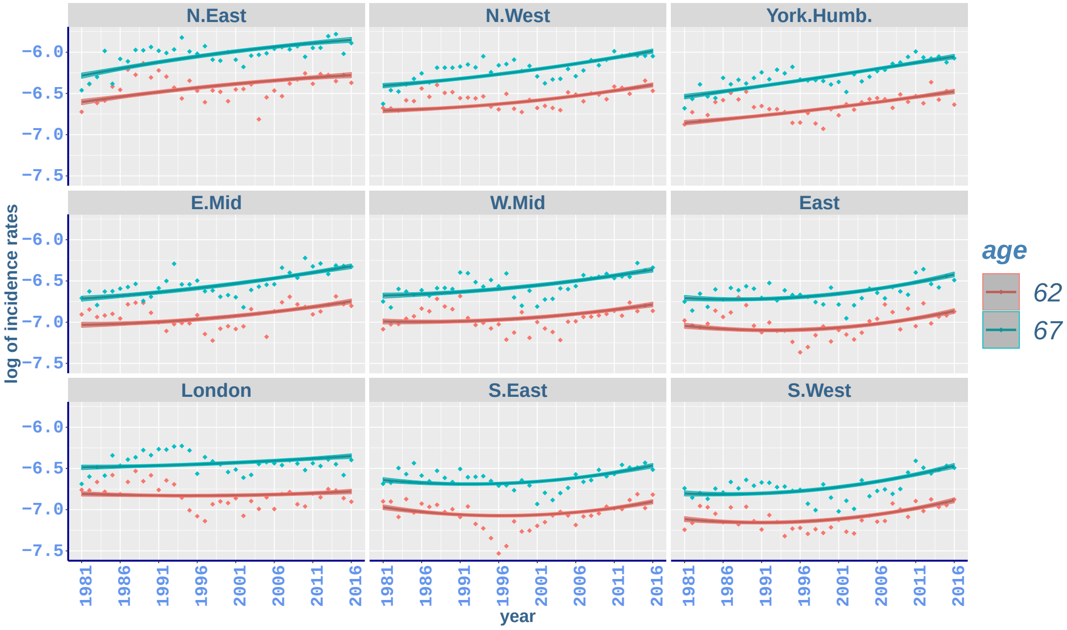


**S22 Fig. Tranchea, bronchus and lung cancer incidence for females, ages 62-67: observed rates (dots), fitted rates (lines) on log-scale, with 95% credible intervals for the fitted rates.**

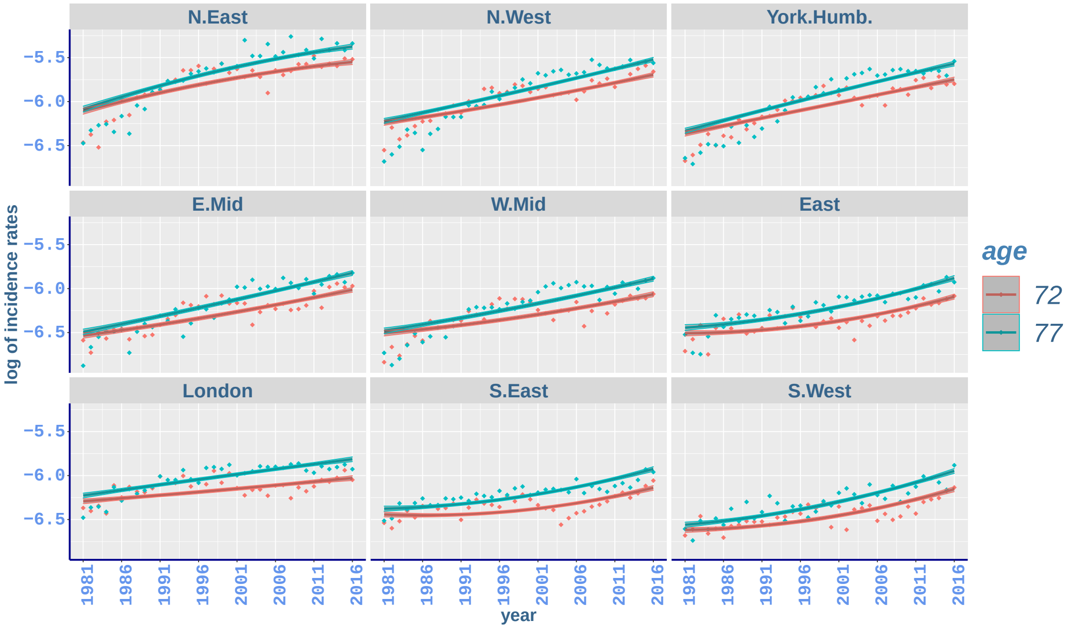


**S23 Fig. Tranchea, bronchus and lung cancer incidence for females, ages 72-77: observed rates (dots), fitted rates (lines) on log-scale, with 95% credible intervals for the fitted rates.**

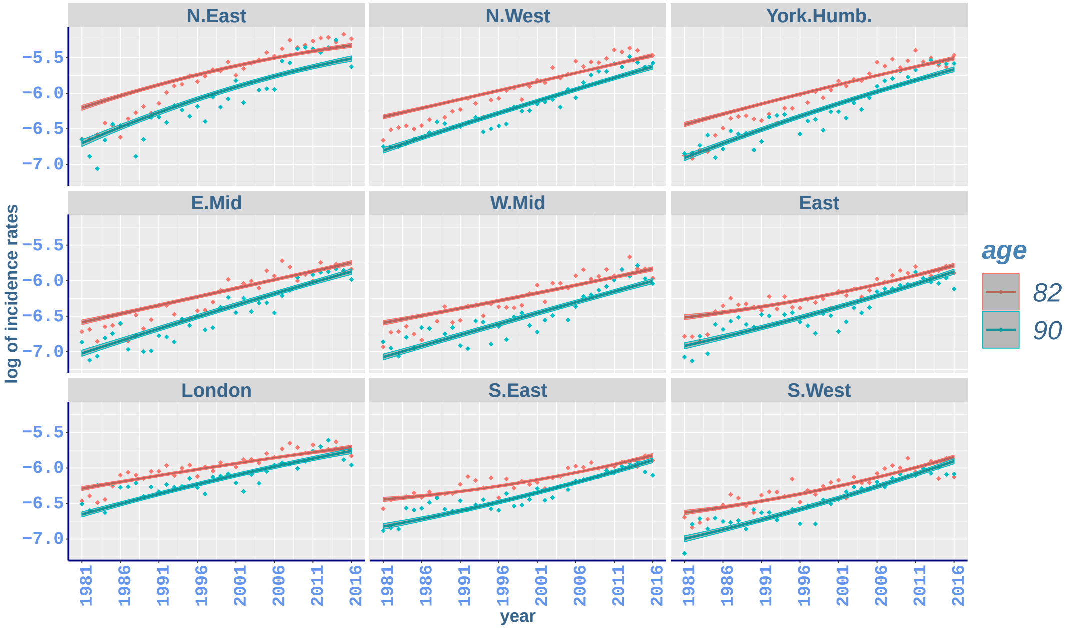


**S24 Fig. Tranchea, bronchus and lung cancer incidence for females, ages 82-90: observed rates (dots), fitted rates (lines) on log-scale, with 95% credible intervals for the fitted rates.**

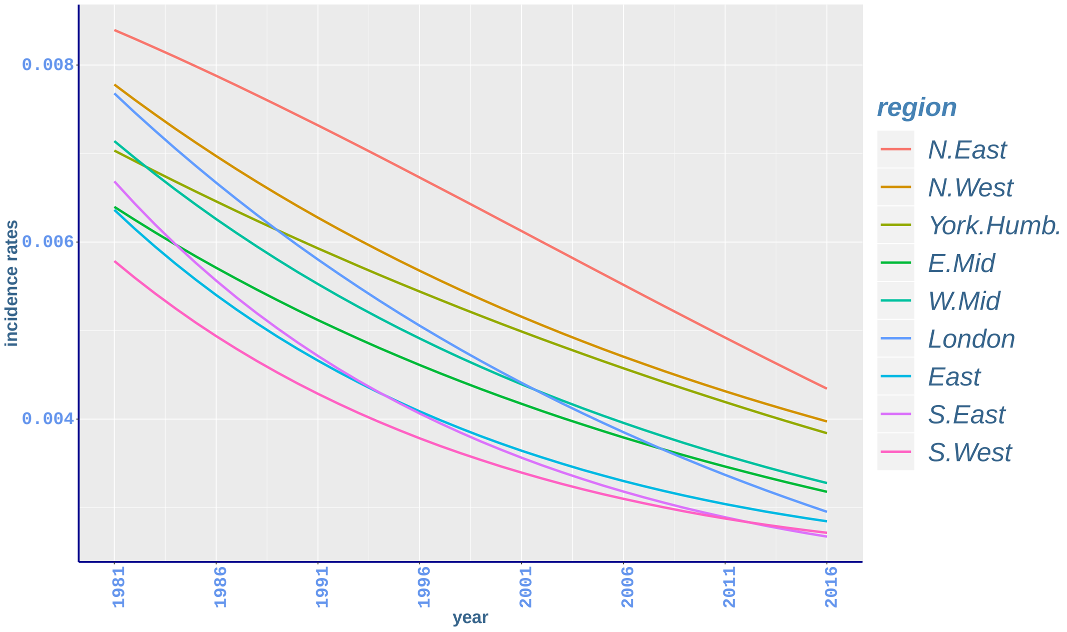


**S25 Fig. Tranchea, bronchus and lung cancer incidence for males, age 72: fitted rates.**

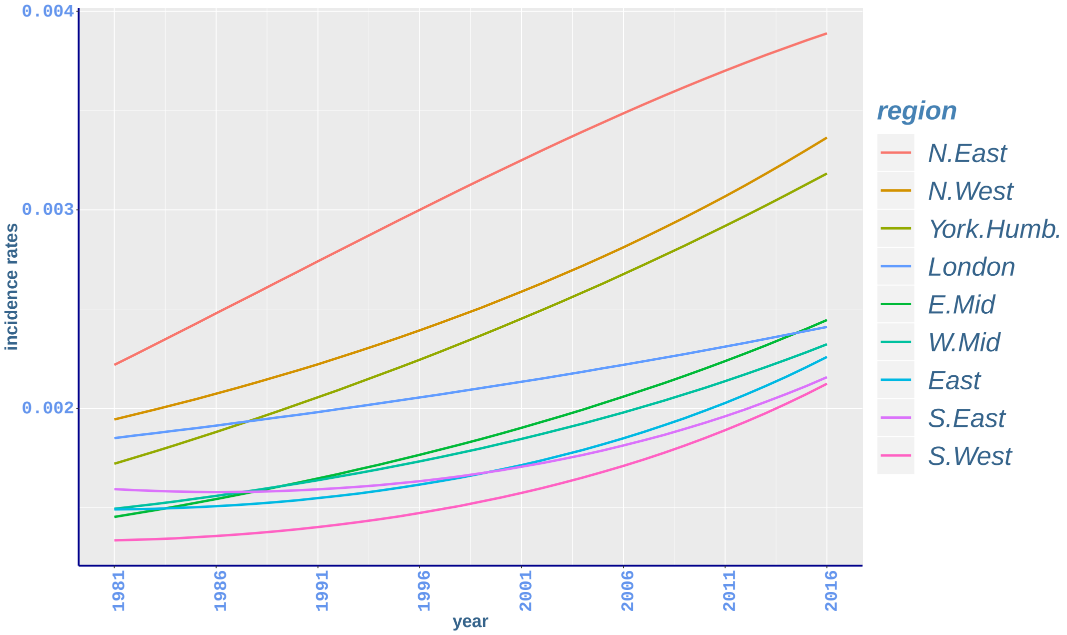


**S26 Fig. Tranchea, bronchus and lung cancer incidence for females, age 72: fitted rates.**

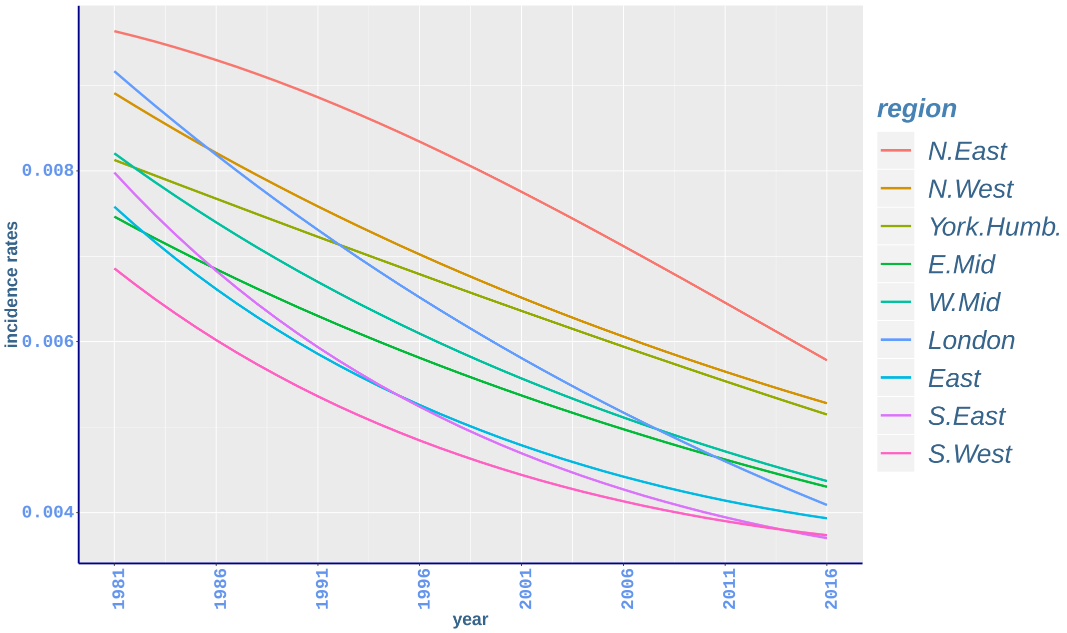


**S27 Fig. Tranchea, bronchus and lung cancer incidence for males, age 77: fitted rates.**

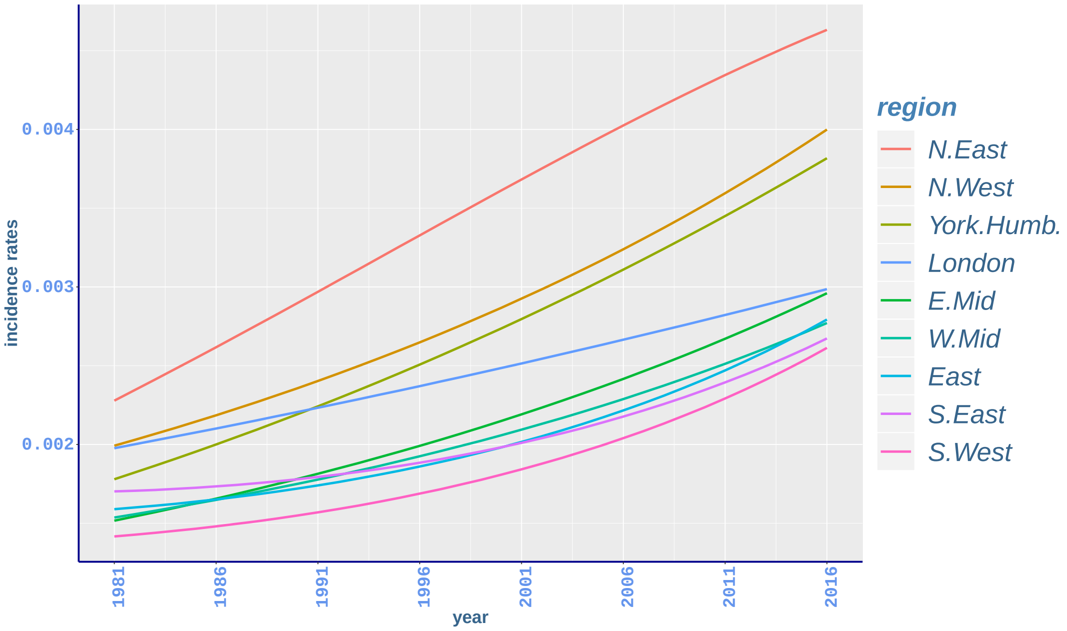


**S28 Fig. Tranchea, bronchus and lung cancer incidence for females, age 77: fitted rates.**

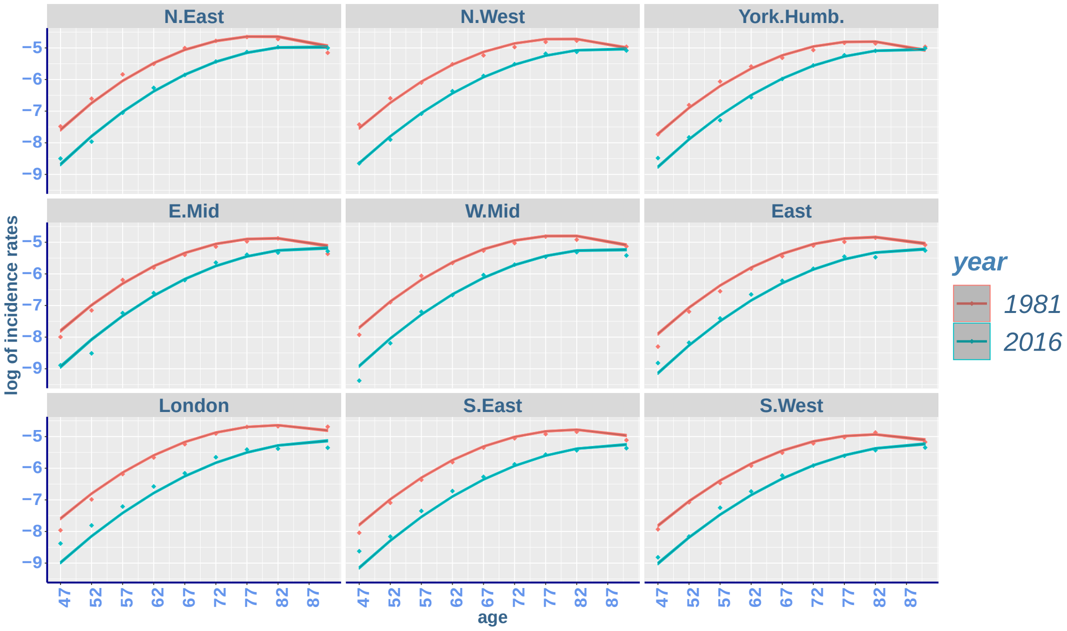


**S29 Fig. Tranchea, bronchus and lung cancer incidence for males, year 1981 and 2016: observed rates (dots), fitted rates (lines) on log-scale, with 95% credible intervals for the fitted rates.**

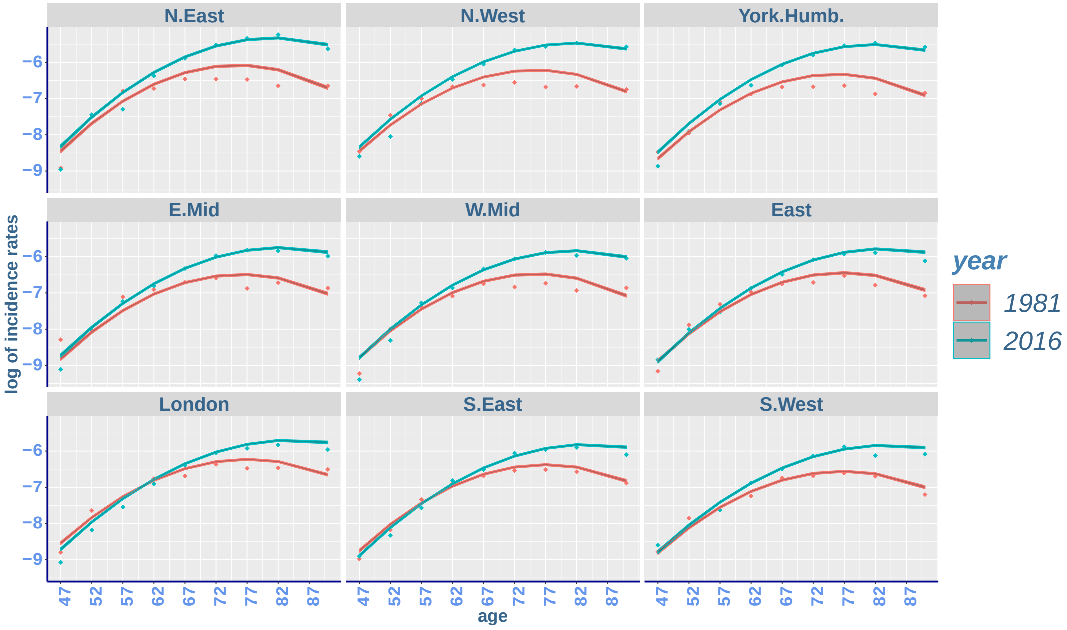


**S30 Fig. Tranchea, bronchus and lung cancer incidence for females, year 1981 and 2016: observed rates (dots), fitted rates (lines) on log-scale, with 95% credible intervals for the fitted rates.**

**S3 Table. Estimated coefficients for the best fitting model for bowel cancer rates.**

| Covariate | Parameter | Mean | SD | %2.5 | %97.5 | Covariate | Parameter | Mean | SD | %2.5 | %97.5 |
|---|---|---|---|---|---|---|---|---|---|---|---|
| Intercept | $\beta_0$ | -6.0730 | 0.0029 | -6.0790 | -6.0670 | Age:Region | $\beta_{15,\text{region}_4}$ | -0.0014 | 0.0044 | -0.0100 | 0.0068 |
| Age | $\beta_1$ | 1.0180 | 0.0027 | 1.0130 | 1.0240 | | $\beta_{15,\text{region}_5}$ | -0.0166 | 0.0039 | -0.0241 | -0.0090 |
| Age$^2$ | $beta_2$ | -0.3173 | 0.0027 | -0.3227 | -0.3121 | | $\beta_{15,\text{region}_6}$ | 0.0145 | 0.0039 | 0.0068 | 0.0223 |
| Year $\leq$ 2006 | $\beta_{3,1}$ | 0.0625 | 0.0055 | 0.0514 | 0.0727 | | $\beta_{15,\text{region}_7}$ | 0.0177 | 0.0039 | 0.0103 | 0.0254 |
| Year $>$ 2006 | $\beta_{3,2}$ | 0.2388 | 0.0142 | 0.2120 | 0.2670 | | $\beta_{15,\text{region}_8}$ | 0.0113 | 0.0033 | 0.0049 | 0.0179 |
| (Year $\leq$ 2006)$^2$ | $\beta_{4,1}$ | -0.0400 | 0.0045 | -0.0495 | -0.0316 | | $\beta_{15,\text{region}_9}$ | 0.0111 | 0.0039 | 0.0036 | 0.0188 |
| (Year $>$ 2006)$^2$ | $\beta_{4,2}$ | -0.1711 | 0.0100 | -0.1912 | -0.1519 | Gender:Region | $\beta_{16,\text{region}_1}$ | -0.1118 | 0.0088 | -0.1290 | -0.0944 |
| Gender | $\beta_5$ | -0.4903 | 0.0043 | -0.4987 | -0.4818 | | $\beta_{16,\text{region}_2}$ | -0.0423 | 0.0062 | -0.0549 | -0.0300 |
| Region | $\beta_{6,\text{region}_1}$ | 0.1450 | 0.0071 | 0.1300 | 0.1586 | | $\beta_{16,\text{region}_3}$ | -0.0440 | 0.0068 | -0.0574 | -0.0308 |
| | $\beta_{6,\text{region}_2}$ | 0.0501 | 0.0053 | 0.0399 | 0.0608 | | $\beta_{16,\text{region}_4}$ | -0.0102 | 0.0076 | -0.0245 | 0.0049 |
| | $\beta_{6,\text{region}_3}$ | 0.0349 | 0.0057 | 0.0237 | 0.0461 | | $\beta_{16,\text{region}_5}$ | -0.0445 | 0.0067 | -0.0580 | -0.0312 |
| | $\beta_{6,\text{region}_4}$ | -0.0165 | 0.0063 | -0.0290 | -0.0045 | | $\beta_{16,\text{region}_6}$ | 0.0709 | 0.0067 | 0.0579 | 0.0843 |
| | $\beta_{6,\text{region}_5}$ | 0.0630 | 0.0055 | 0.0521 | 0.0739 | | $\beta_{16,\text{region}_7}$ | 0.0564 | 0.0067 | 0.0431 | 0.0691 |
| | $\beta_{6,\text{region}_6}$ | -0.0643 | 0.0056 | -0.0751 | -0.0532 | | $\beta_{16,\text{region}_8}$ | 0.0661 | 0.0057 | 0.0548 | 0.0773 |
| | $\beta_{6,\text{region}_7}$ | -0.1459 | 0.0058 | -0.1572 | -0.1346 | | $\beta_{16,\text{region}_9}$ | 0.0595 | 0.0065 | 0.0476 | 0.0728 |
| | $\beta_{6,\text{region}_8}$ | -0.0574 | 0.0050 | -0.0676 | -0.0476 | (Year $\leq$ 2006) : Region | $\beta_{17,1,\text{region}_1}$ | 0.0420 | 0.0069 | 0.0286 | 0.0550 |
| | $\beta_{6,\text{region}_9}$ | -0.0089 | 0.0055 | -0.0199 | 0.0021 | | $\beta_{17,1,\text{region}_2}$ | -0.0058 | 0.0052 | -0.0160 | 0.0044 |
| Age : (Year $\leq$ 2006) | $\beta_{7,1}$ | 0.0113 | 0.0045 | 0.0023 | 0.0198 | | $\beta_{17,1,\text{region}_3}$ | -0.0193 | 0.0055 | -0.0304 | -0.0083 |
| Age : (Year $>$ 2006) | $\beta_{7,2}$ | 0.0541 | 0.0120 | 0.0302 | 0.0767 | | $\beta_{17,1,\text{region}_4}$ | -0.0051 | 0.0061 | -0.0176 | 0.0066 |
| Age : (Year $\leq$ 2006)$^2$ | $\beta_{8,1}$ | 0.0030 | 0.0040 | -0.0053 | 0.0108 | | $\beta_{17,1,\text{region}_5}$ | -0.0328 | 0.0056 | -0.0432 | -0.0219 |
| Age : (Year $>$ 2006)$^2$ | $\beta_{8,2}$ | -0.0539 | 0.0083 | -0.0695 | -0.0372 | | $\beta_{17,1,\text{region}_6}$ | 0.0138 | 0.0056 | 0.0030 | 0.0250 |
| Age$^2$ : (Year $\leq$ 2006) | $\beta_{9,1}$ | -0.0357 | 0.0042 | -0.0438 | -0.0272 | | $\beta_{17,1,\text{region}_7}$ | 0.0288 | 0.0056 | 0.0176 | 0.0399 |
| Age$^2$ : (Year $>$ 2006) | $\beta_{9,2}$ | -0.1170 | 0.0107 | -0.1369 | -0.0938 | | $\beta_{17,1,\text{region}_8}$ | -0.0226 | 0.0049 | -0.0321 | -0.0130 |
| Age$^2$ : (Year $\leq$ 2006)$^2$ | $\beta_{10,1}$ | 0.0097 | 0.0037 | 0.0024 | 0.0174 | | $\beta_{17,1,\text{region}_9}$ | 0.0009 | 0.0055 | -0.0099 | 0.0117 |
| Age$^2$ : (Year $>$ 2006)$^2$ | $\beta_{10,2}$ | 0.0891 | 0.0073 | 0.0730 | 0.1030 | (Year $>$ 2006) : Region | $\beta_{17,2,\text{region}_1}$ | -0.0243 | 0.0074 | -0.0388 | -0.0098 |
| Age:Gender | $\beta_{11}$ | -0.0784 | 0.0030 | -0.0842 | -0.0724 | | $\beta_{17,2,\text{region}_2}$ | 0.0108 | 0.0054 | 0.0003 | 0.0214 |
| (Age)$^2$ : Gender | $\beta_{12}$ | 0.0869 | 0.0028 | 0.0815 | 0.0924 | | $\beta_{17,2,\text{region}_3}$ | -0.0156 | 0.0060 | -0.0268 | -0.0038 |
| (Year $\leq$ 2006) : Gender | $\beta_{13,1}$ | -0.0449 | 0.0068 | -0.0582 | -0.0317 | | $\beta_{17,2,\text{region}_4}$ | 0.0093 | 0.0061 | -0.0022 | 0.0216 |
| (Year $>$ 2006) : Gender | $\beta_{13,2}$ | -0.0380 | 0.0187 | -0.0769 | -0.0042 | | $\beta_{17,2,\text{region}_5}$ | -0.0196 | 0.0057 | -0.0307 | -0.0082 |
| (Year $\leq$ 2006)$^2$ : Gender | $\beta_{14,1}$ | 0.0230 | 0.0058 | 0.0116 | 0.0341 | | $\beta_{17,2,\text{region}_6}$ | 0.0173 | 0.0057 | 0.0057 | 0.0286 |
| (Year $<$ 2006)$^2$ : Gender | $\beta_{14,2}$ | 0.0382 | 0.0129 | 0.0154 | 0.0644 | | $\beta_{17,2,\text{region}_7}$ | 0.0128 | 0.0059 | 0.0015 | 0.0245 |
| Age:Region | $\beta_{15,\text{region}_1}$ | -0.0129 | 0.0049 | -0.0230 | -0.0035 | | $\beta_{17,2,\text{region}_8}$ | 0.0083 | 0.0053 | -0.0016 | 0.0190 |
| | $\beta_{15,\text{region}_2}$ | -0.0178 | 0.0036 | -0.0249 | -0.0107 | | $\beta_{17,2,\text{region}_9}$ | 0.0010 | 0.0057 | -0.0100 | 0.0120 |
| | $\beta_{15,\text{region}_3}$ | -0.0059 | 0.0040 | -0.0136 | 0.0019 | | $\sigma^2$ | 0.0020 | 0.0001 | 0.0017 | 0.0023 |

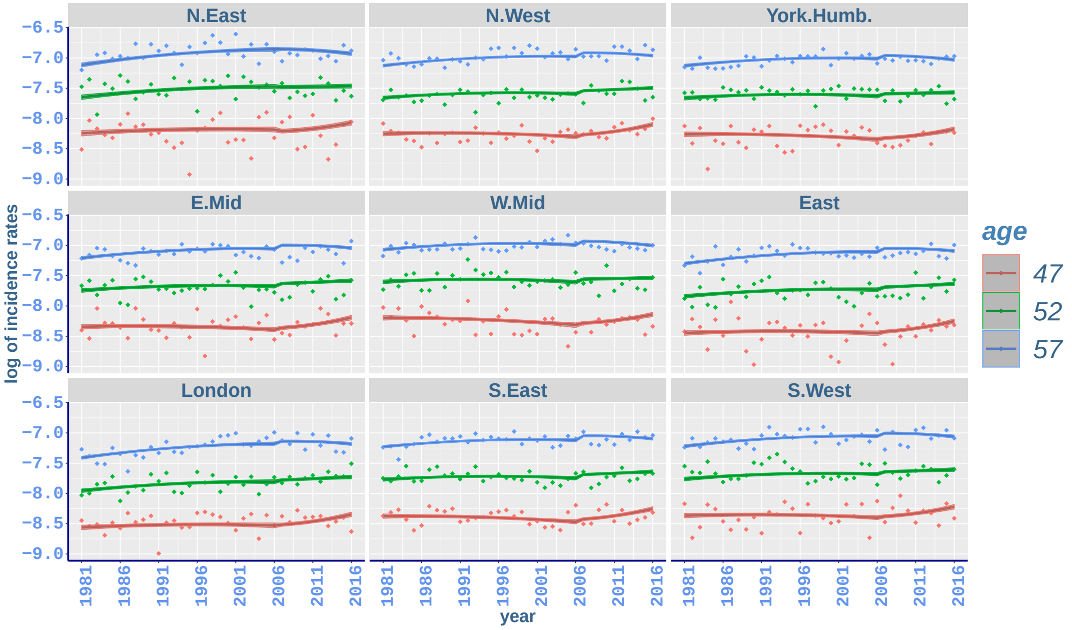


**S31 Fig. Bowel cancer incidence for males, ages 47-57: observed rates (dots), fitted rates (lines) on log-scale, with 95% credible intervals for the fitted rates.**

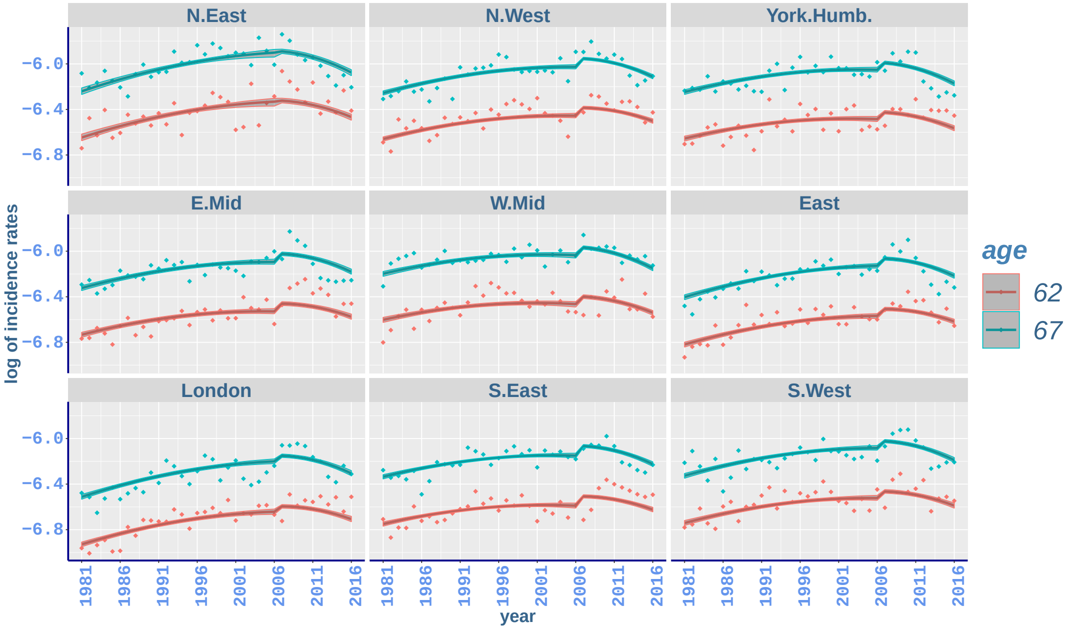


**S32 Fig. Bowel cancer incidence for males, ages 62-67: observed rates (dots), fitted rates (lines) on log-scale, with 95% credible intervals for the fitted rates.**

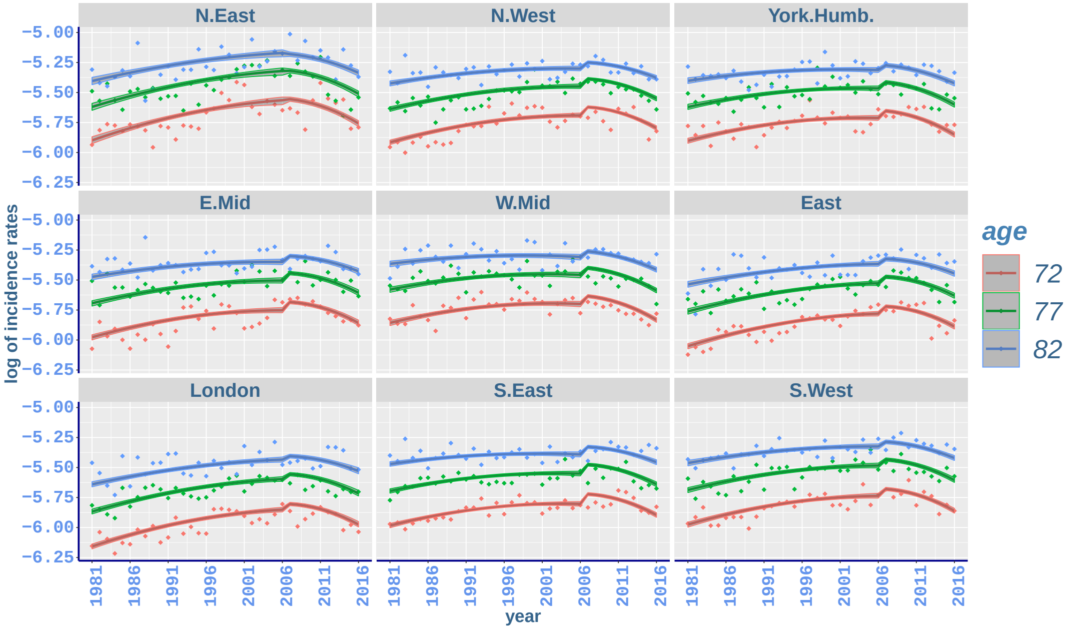


**S33 Fig. Bowel cancer incidence for males, ages 72-82: observed rates (dots), fitted rates (lines) on log-scale, with 95% credible intervals for the fitted rates.**

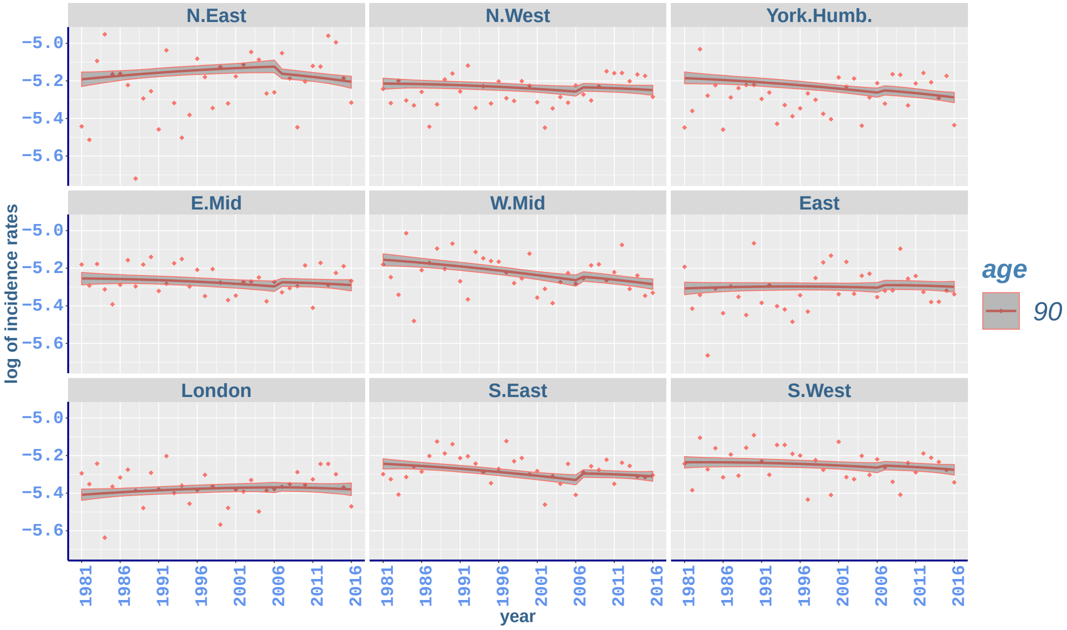


**S34 Fig. Bowel cancer incidence for males, age 90: observed rates (dots), fitted rates (lines) on log-scale, with 95% credible intervals for the fitted rates.**

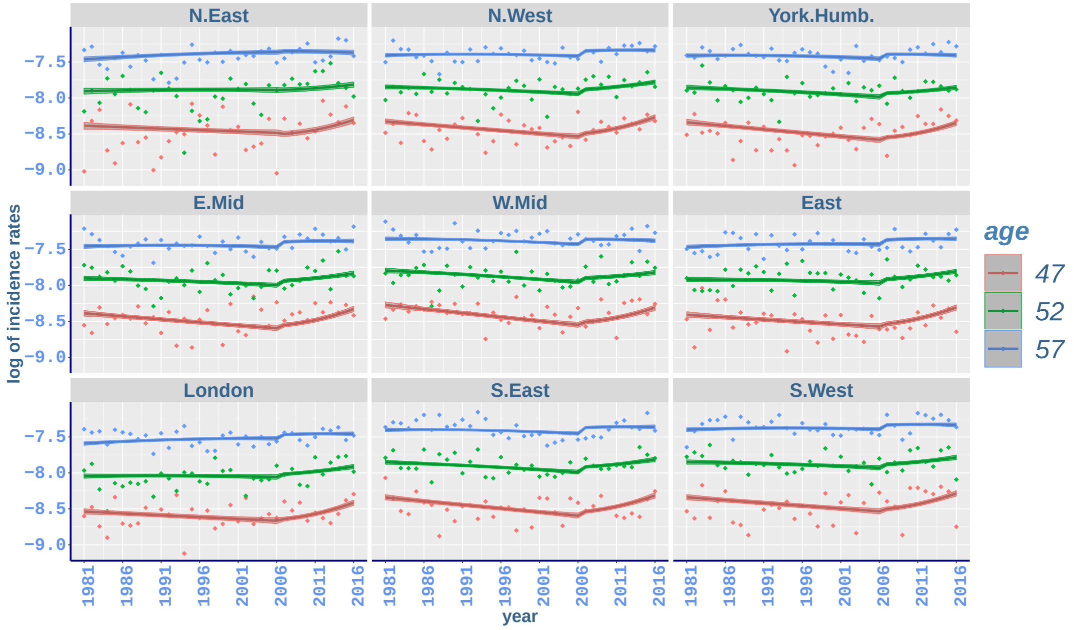


**S35 Fig. Bowel cancer incidence for females, ages 47-57: observed rates (dots), fitted rates (lines) on log-scale, with 95% credible intervals for the fitted rates.**

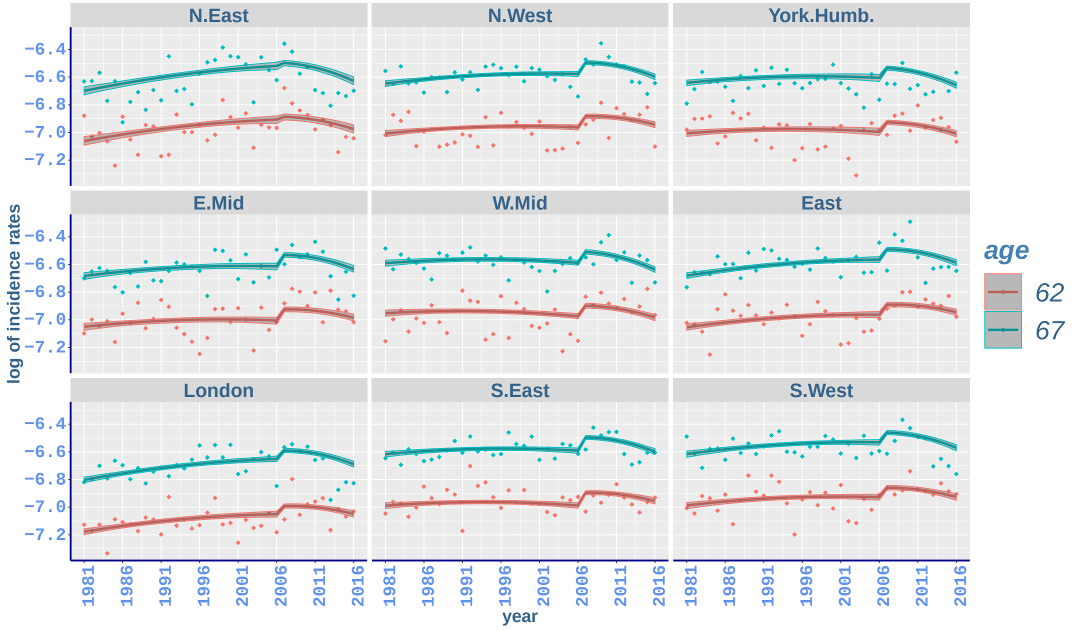


**S36 Fig. Bowel cancer incidence for females, ages 62-67: observed rates (dots), fitted rates (lines) on log-scale, with 95% credible intervals for the fitted rates.**

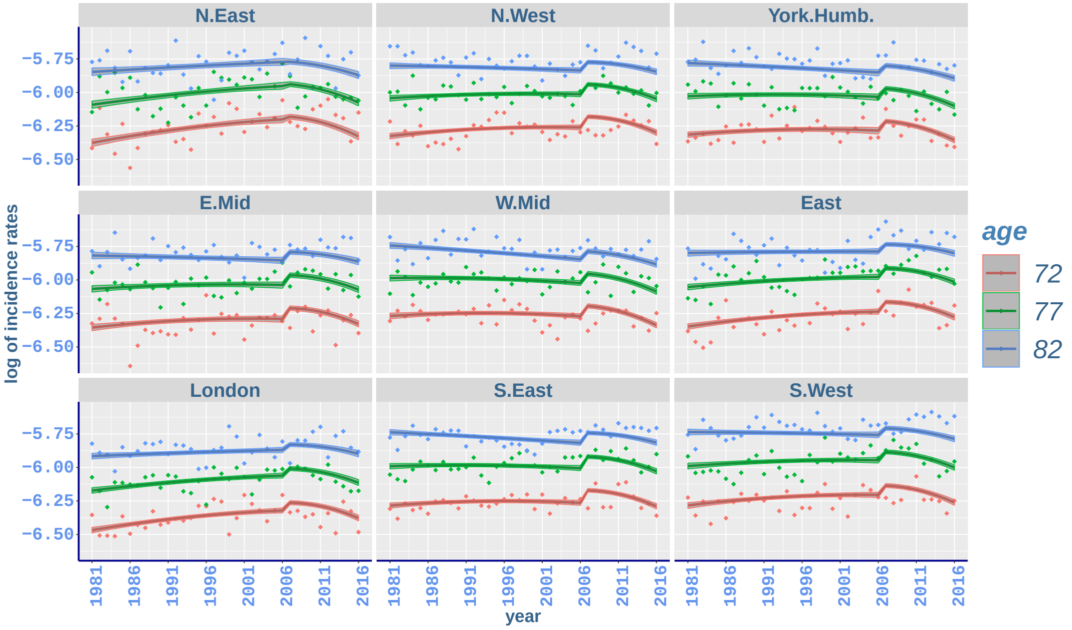


**S37 Fig. Bowel cancer incidence for females, ages 72-82: observed rates (dots), fitted rates (lines) on log-scale, with 95% credible intervals for the fitted rates.**

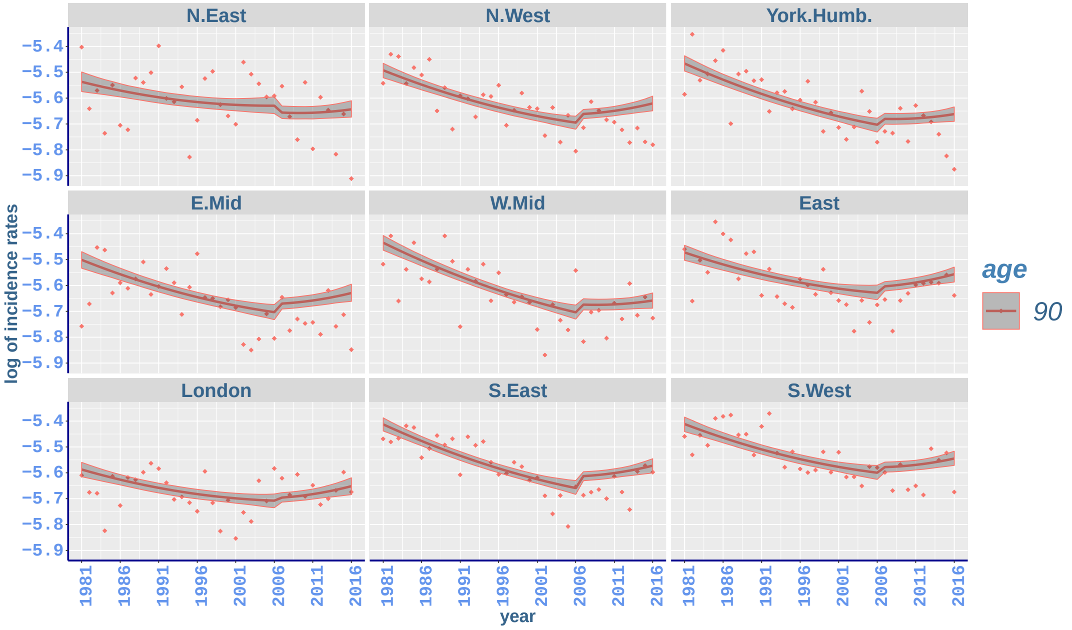


**S38 Fig. Bowel cancer incidence for females, age 90: observed rates (dots), fitted rates (lines) on log-scale, with 95% credible intervals for the fitted rates.**

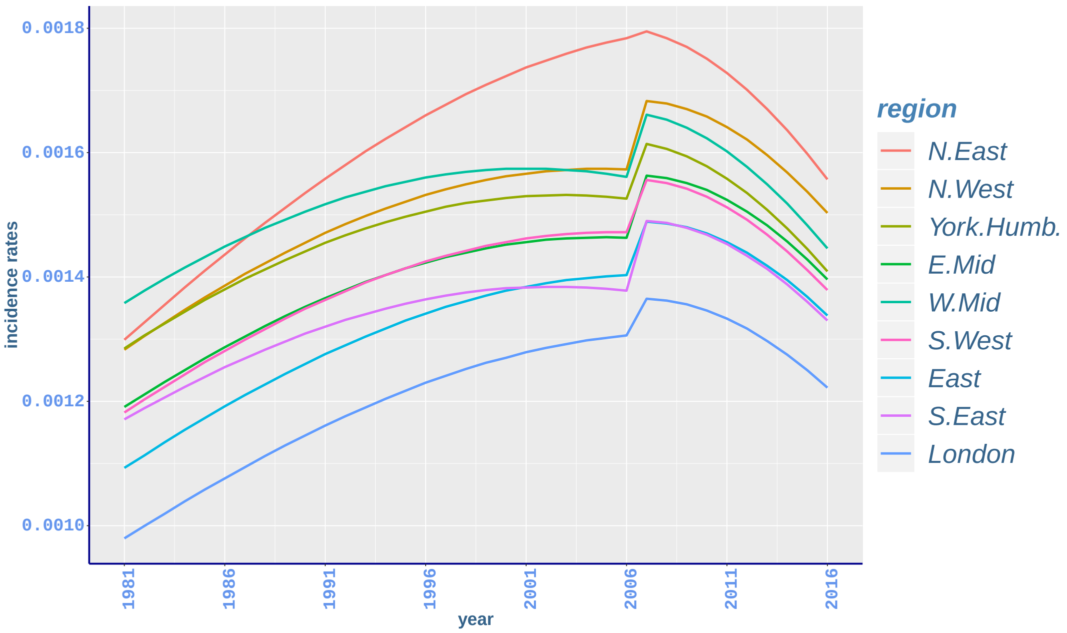


**S39 Fig. Bowel cancer incidence for males, age 62: fitted rates.**

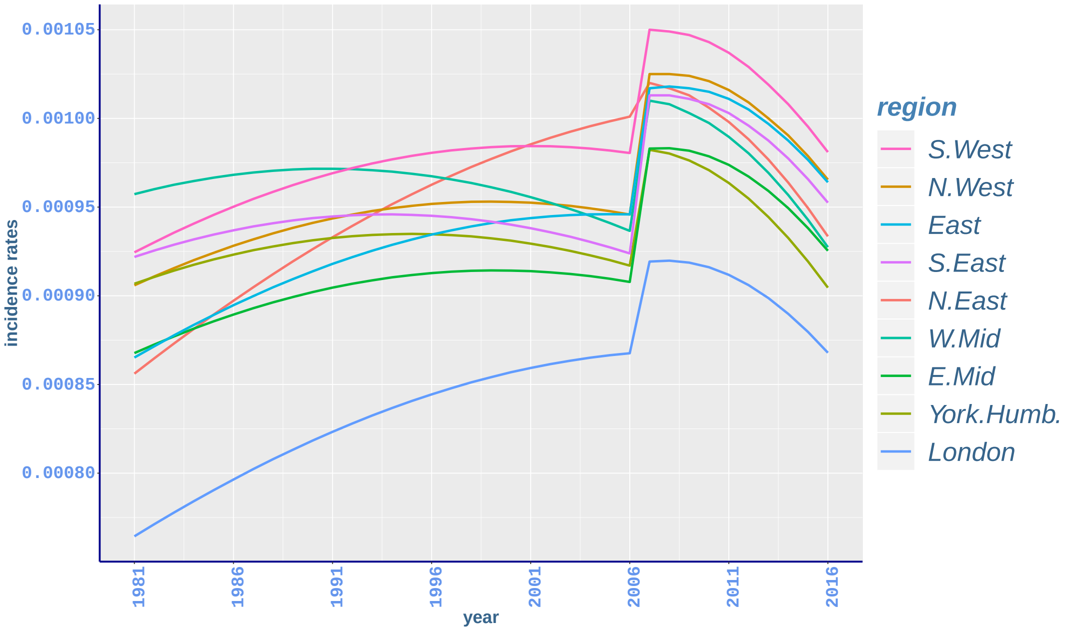


**S40 Fig. Bowel cancer incidence for females, age 62: fitted rates.**

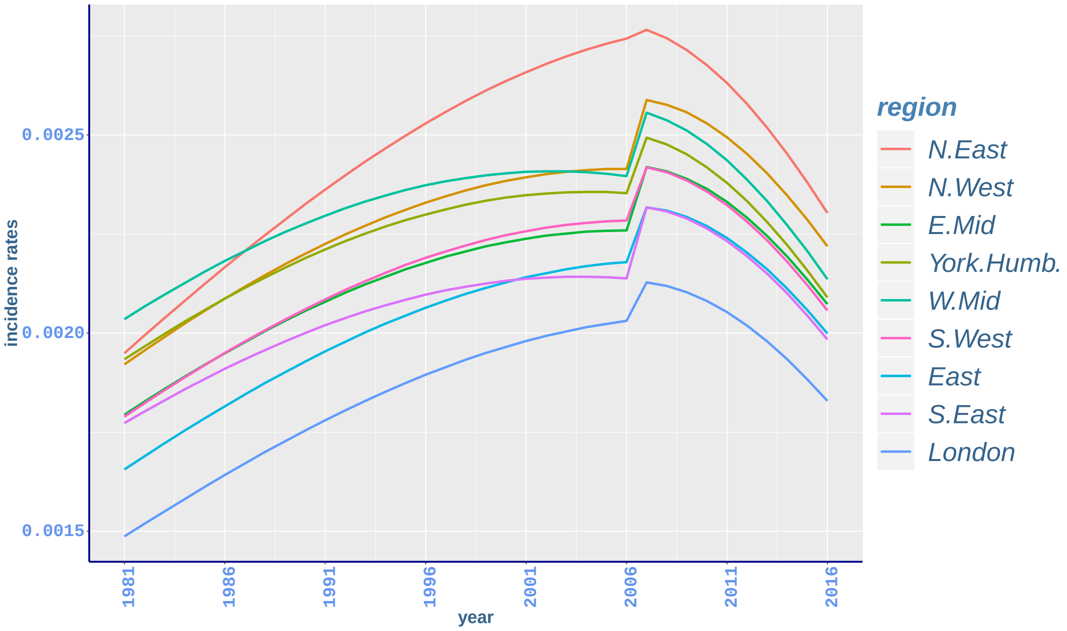


**S41 Fig. Bowel cancer incidence for males, age 67: fitted rates.**

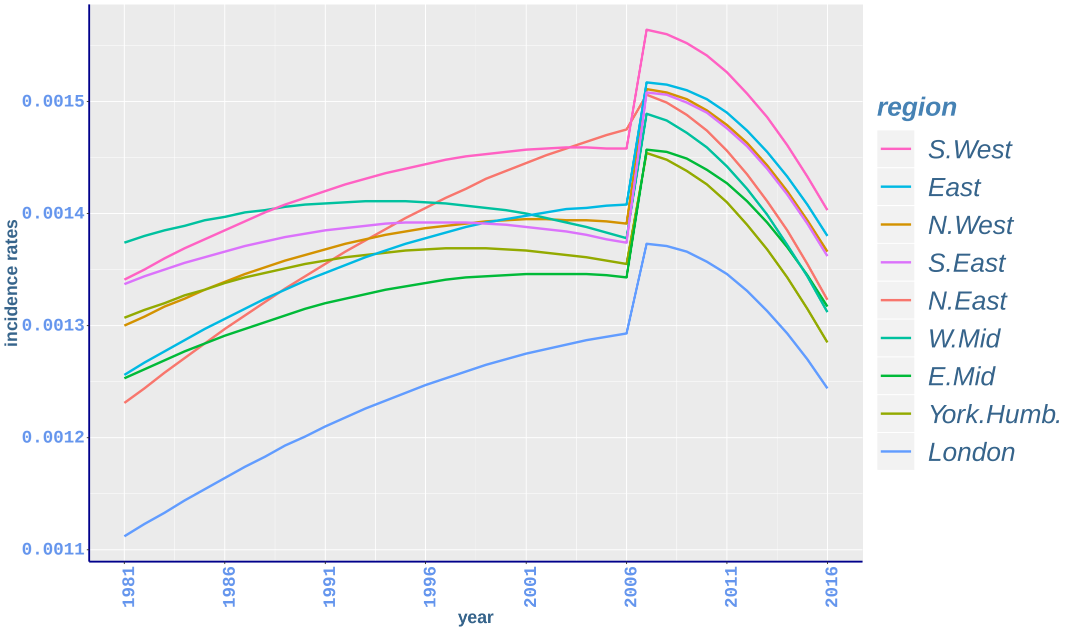


**S42 Fig. Bowel cancer incidence for females, age 67: fitted rates.**

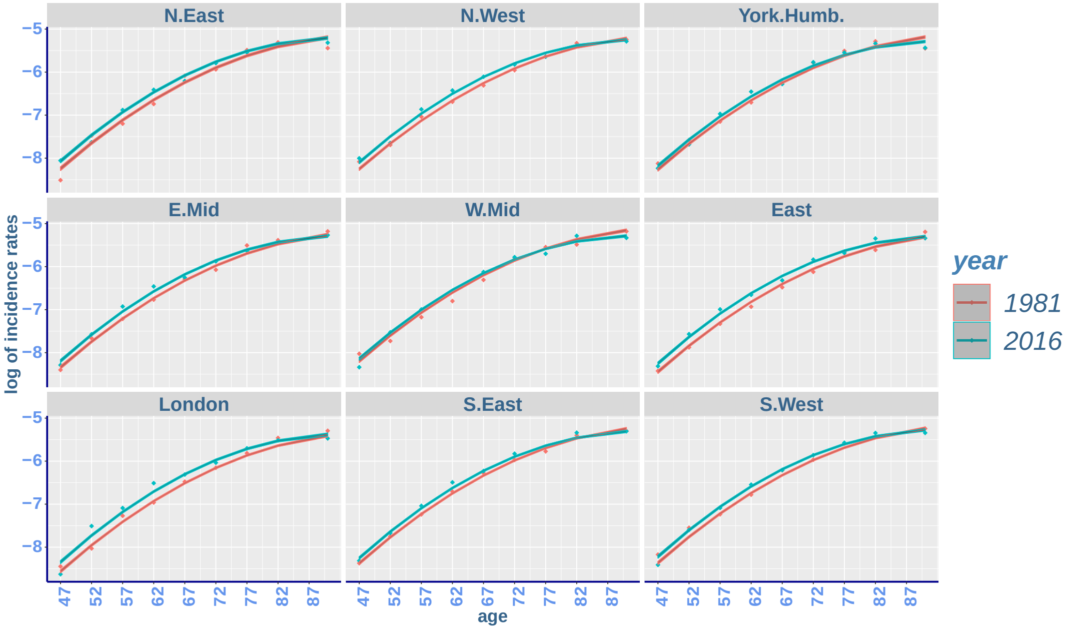


**S43 Fig. Bowel cancer incidence for males, year 1981 and 2016: observed rates (dots), fitted rates (lines) on log-scale, with 95% credible intervals for the fitted rates.**

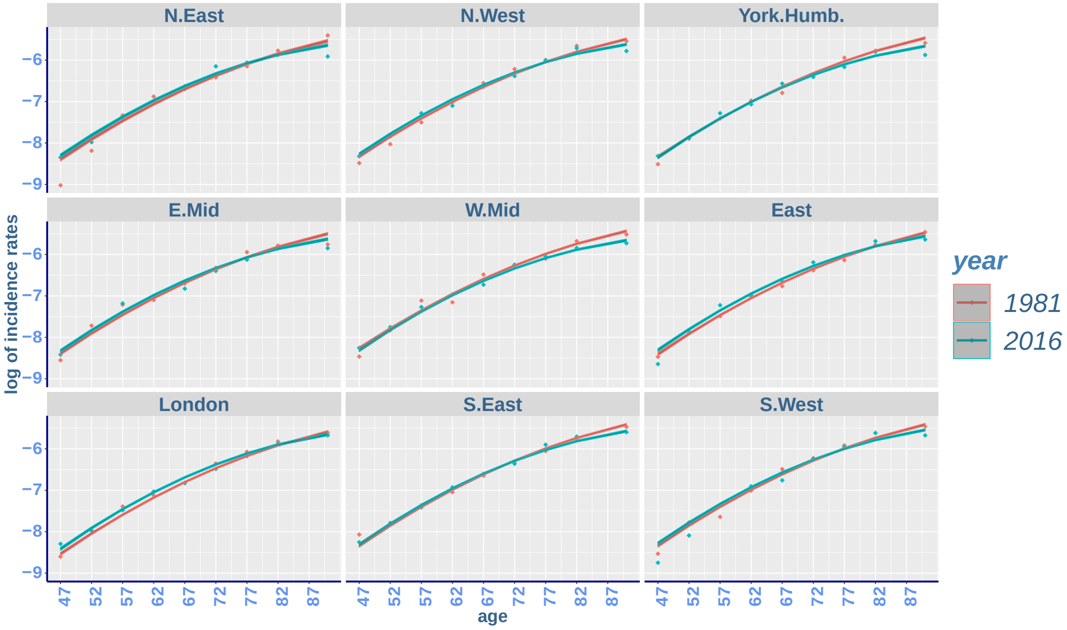


**S44 Fig. Bowel cancer incidence for females, year 1981 and 2016: observed rates (dots), fitted rates (lines) on log-scale, with 95% credible intervals for the fitted rates.**

**S4 Table. Estimated coefficients for the best fitting model for prostate cancer rates.**

| | | | | | | | | | | | |
|---|---|---|---|---|---|---|---|---|---|---|---|
| Intercept | $\beta_0$ | -5.6640 | 0.0042 | -5.6720 | -5.6560 | | $\beta_{7,\text{region}_9}$ | 0.0728 | 0.0065 | 0.0600 | 0.0853 |
| Age | $\beta_1$ | 1.4130 | 0.0061 | 1.4010 | 1.4250 | Age:Year | $\beta_8$ | -0.5106 | 0.0082 | -0.5267 | -0.4946 |
| $\text{Age}^2$ | $\beta_2$ | -0.6896 | 0.0043 | -0.6975 | -0.6807 | Age : $\text{Year}^2$ | $\beta_9$ | -0.0479 | 0.0031 | -0.0536 | -0.0417 |
| $\text{Age}^3$ | $\beta_3$ | 0.1329 | 0.0036 | 0.1256 | 0.1400 | Age : $\text{Year}^3$ | $\beta_{10}$ | 0.0776 | 0.0035 | 0.0702 | 0.0842 |
| Year | $\beta_4$ | 0.6719 | 0.0060 | 0.6605 | 0.6838 | $\text{Age}^2$ : Year | $\beta_{11}$ | -0.0168 | 0.0039 | -0.0247 | -0.0098 |
| $\text{Year}^2$ | $\beta_5$ | -0.0781 | 0.0027 | -0.0835 | -0.0727 | $\text{Age}^3$ : Year | $\beta_{12}$ | 0.0460 | 0.0034 | 0.0397 | 0.0528 |
| $\text{Year}^3$ | $\beta_6$ | -0.0973 | 0.0030 | -0.1030 | -0.0915 | Age:Region | $\beta_{13,\text{region}_1}$ | 0.0066 | 0.0089 | -0.0107 | 0.0244 |
| Region | $\beta_{7,\text{region}_1}$ | -0.1265 | 0.0077 | -0.1413 | -0.1116 | | $\beta_{13,\text{region}_2}$ | 0.0163 | 0.0071 | 0.0017 | 0.0299 |
| | $\beta_{7,\text{region}_2}$ | -0.0521 | 0.0063 | -0.0644 | -0.0396 | | $\beta_{13,\text{region}_3}$ | -0.0067 | 0.0074 | -0.0211 | 0.0084 |
| | $\beta_{7,\text{region}_3}$ | -0.0374 | 0.0066 | -0.0506 | -0.0243 | | $\beta_{13,\text{region}_4}$ | 0.0456 | 0.0077 | 0.0303 | 0.0604 |
| | $\beta_{7,\text{region}_4}$ | -0.0593 | 0.0068 | -0.0727 | -0.0458 | | $\beta_{13,\text{region}_5}$ | 0.0011 | 0.0075 | -0.0139 | 0.0154 |
| | $\beta_{7,\text{region}_5}$ | 0.0108 | 0.0064 | -0.0019 | 0.0231 | | $\beta_{13,\text{region}_6}$ | 0.0104 | 0.0073 | -0.0039 | 0.0247 |
| | $\beta_{7,\text{region}_6}$ | 0.0507 | 0.0064 | 0.0379 | 0.0628 | | $\beta_{13,\text{region}_7}$ | -0.0966 | 0.0068 | -0.1098 | -0.0831 |
| | $\beta_{7,\text{region}_7}$ | 0.0687 | 0.0063 | 0.0568 | 0.0814 | | $\beta_{13,\text{region}_8}$ | -0.0233 | 0.0065 | -0.0361 | -0.0104 |
| | $\beta_{7,\text{region}_8}$ | 0.0725 | 0.0059 | 0.0611 | 0.0838 | | $\beta_{13,\text{region}_9}$ | 0.0465 | 0.0071 | 0.0323 | 0.0605 |
| | | | | | | | $\sigma^2$ | 0.0071 | 0.0003 | 0.0065 | 0.0077 |

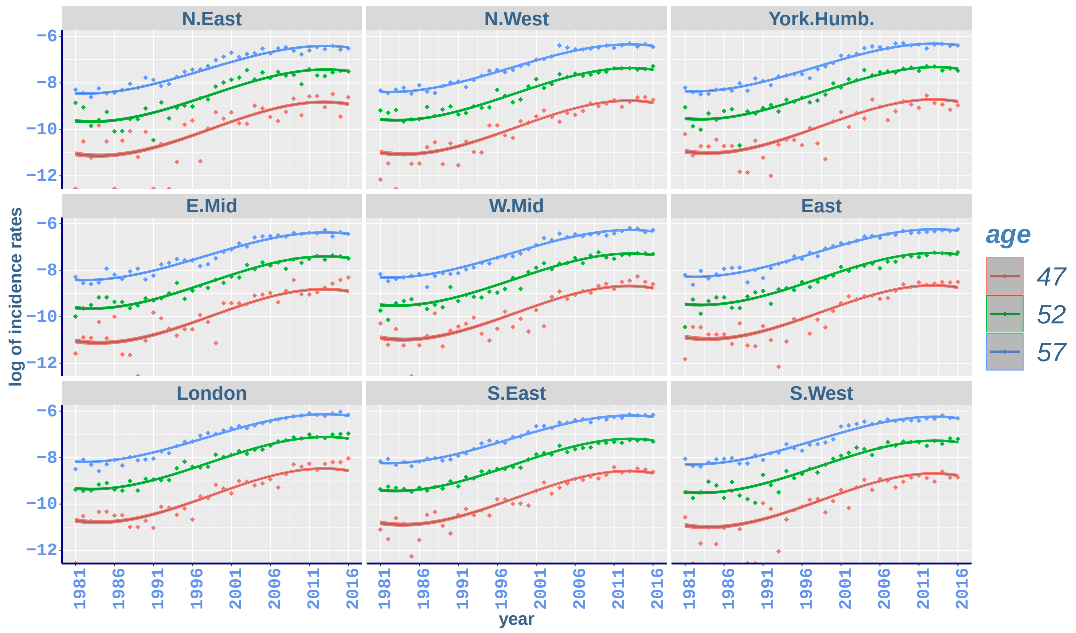


S45 Fig. Prostate cancer incidence, ages 47-57: observed rates (dots), fitted rates (lines) on log-scale, with 95% credible intervals for the fitted rates.

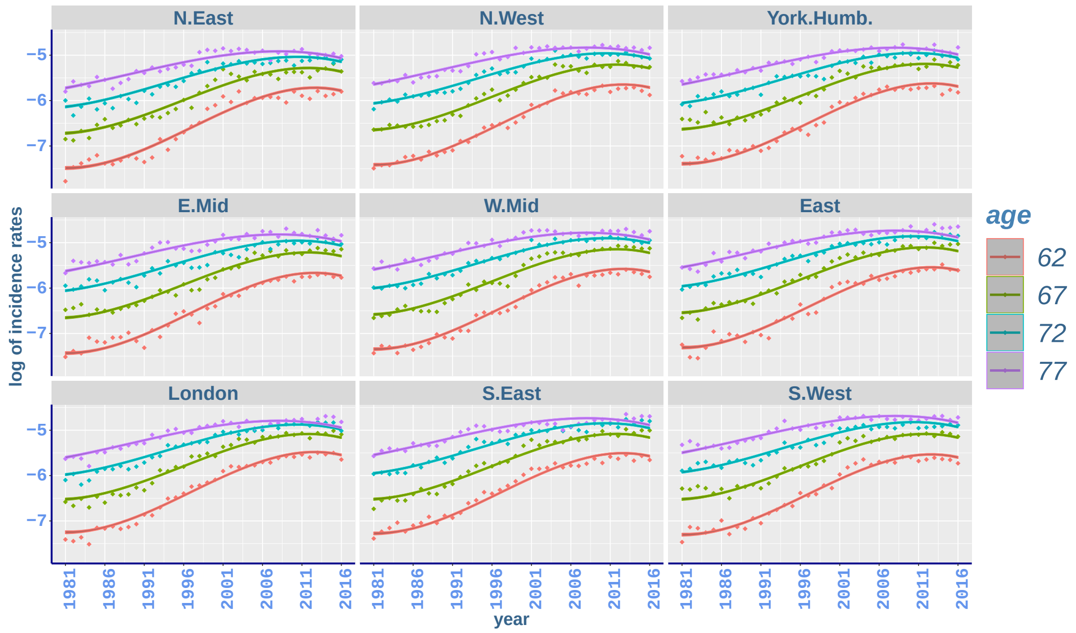


S46 Fig. Prostate cancer incidence, ages 62-77: observed rates (dots), fitted rates (lines) on log-scale, with 95% credible intervals for the fitted rates.

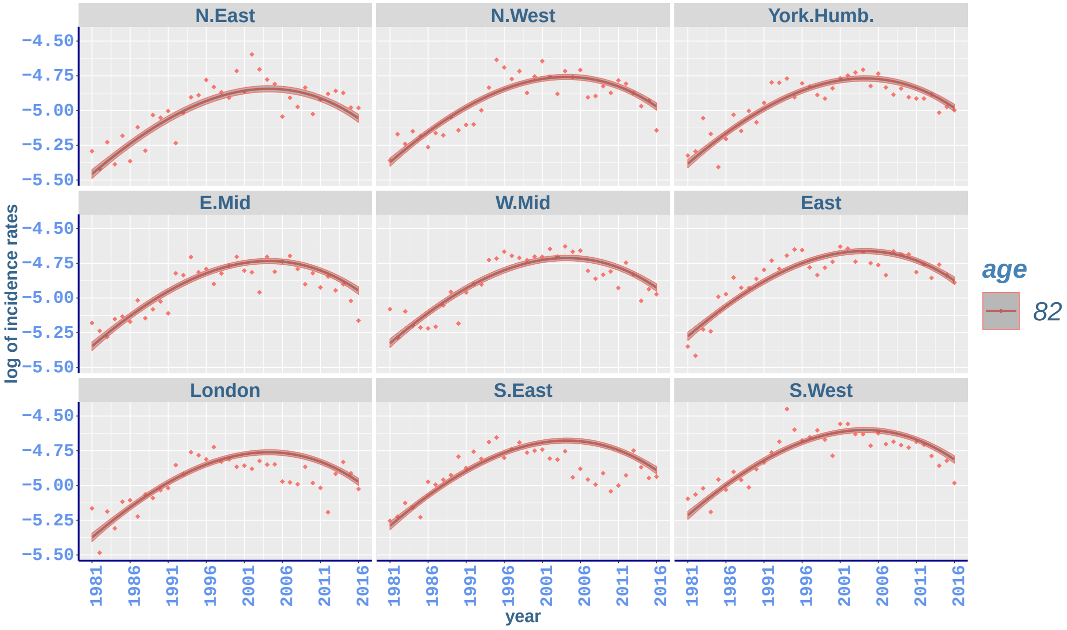


**S47 Fig. Prostate cancer incidence, age 82: observed rates (dots), fitted rates (lines) on log-scale, with 95% credible intervals for the fitted rates.**

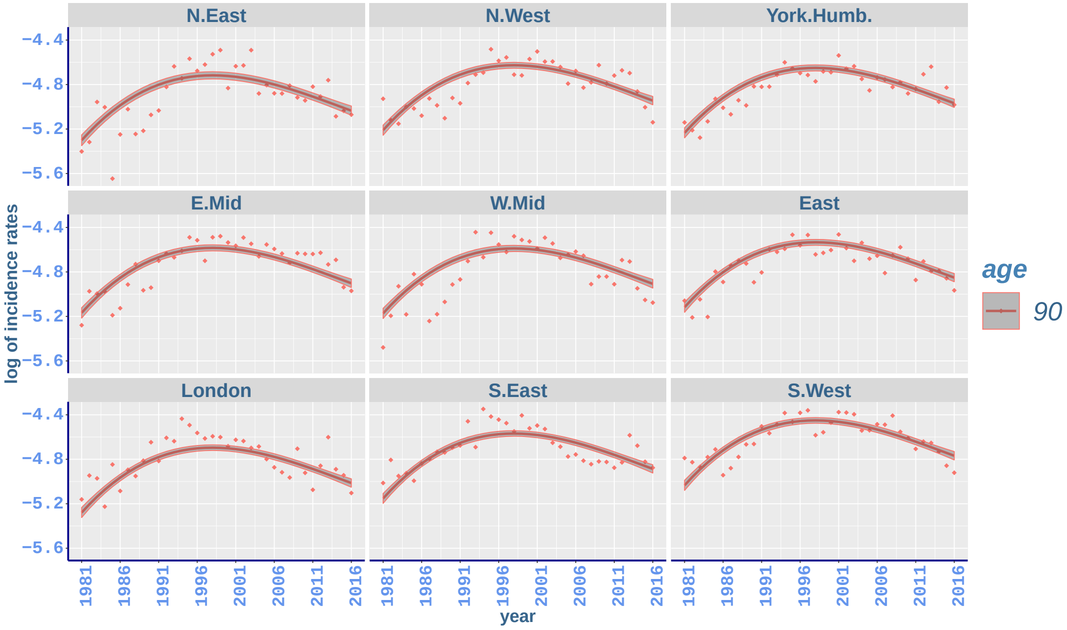


**S48 Fig. Prostate cancer incidence, age 90: observed rates (dots), fitted rates (lines) on log-scale, with 95% credible intervals for the fitted rates.**

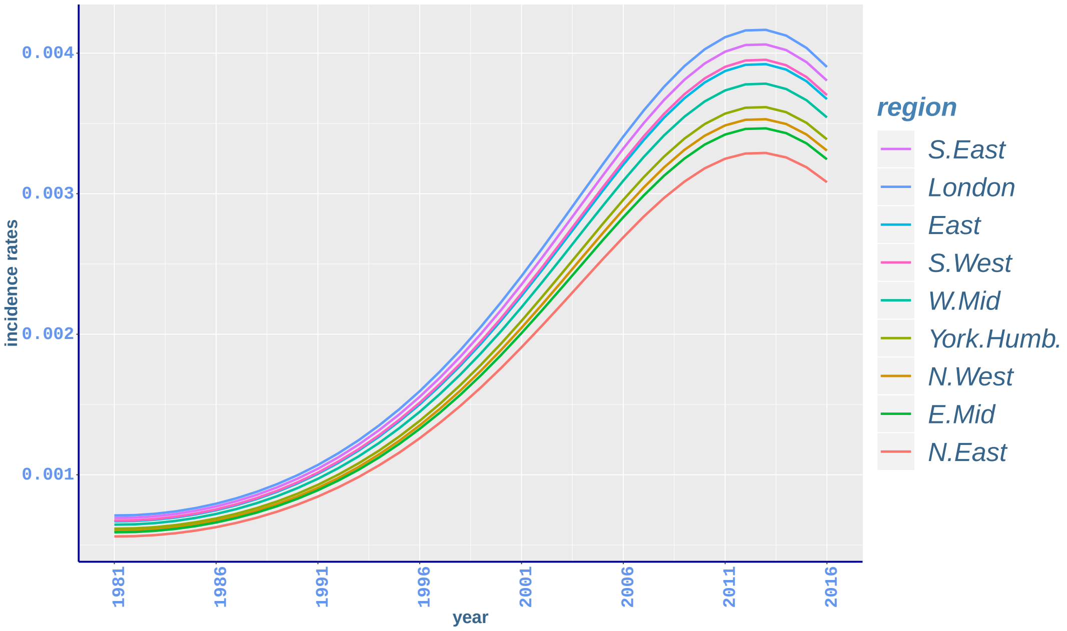


**S49 Fig. Prostate cancer incidence, age 62: fitted rates.**

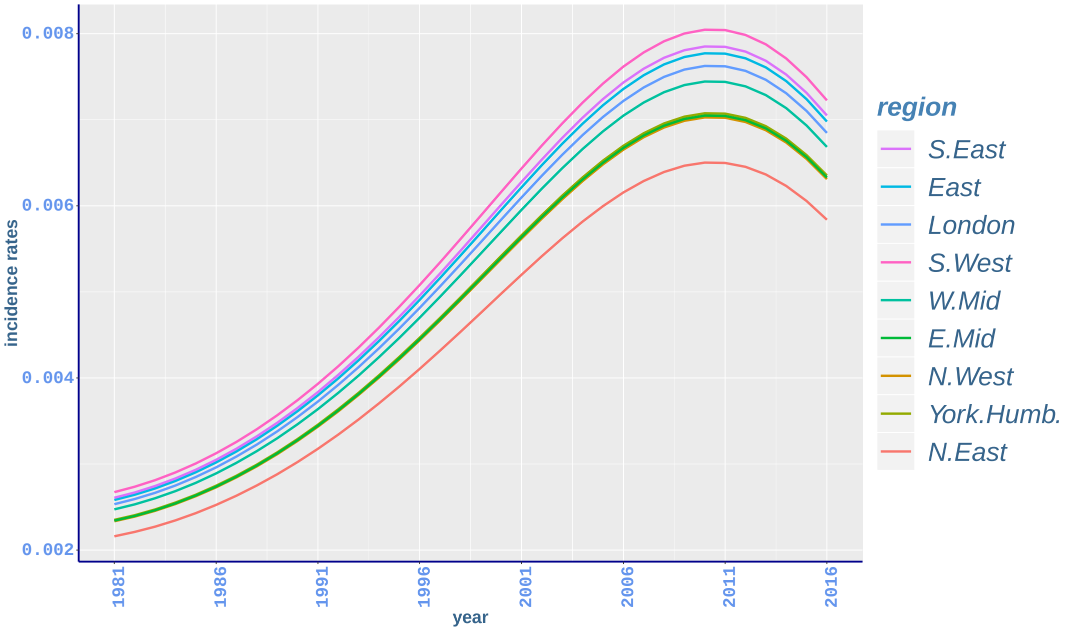


**S50 Fig. Prostate cancer incidence, age 72: fitted rates.**

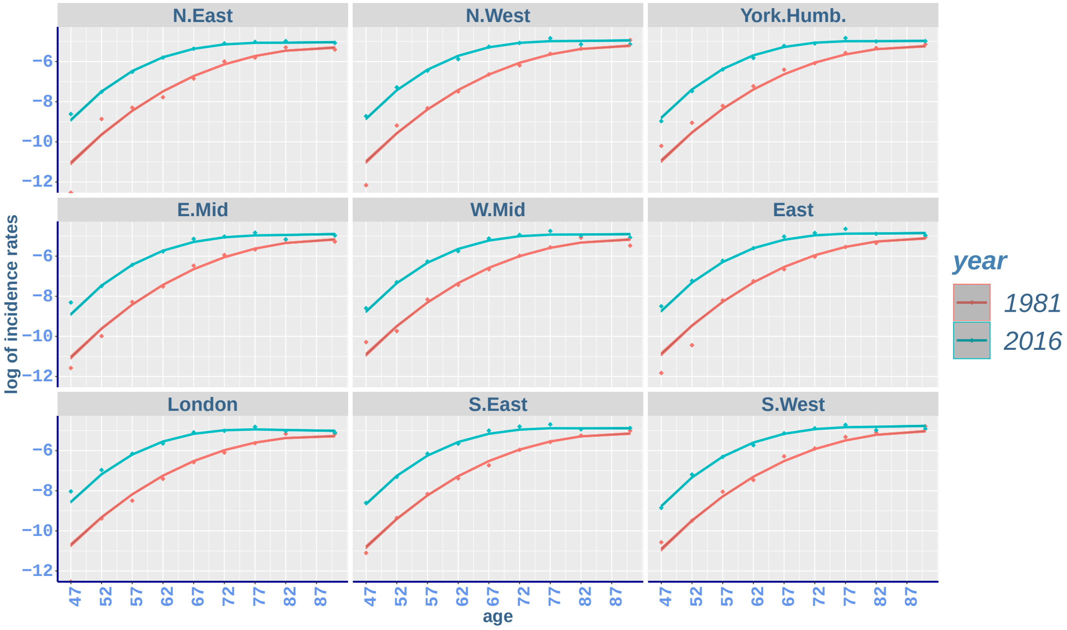


**S51 Fig. Prostate cancer incidence, year 1981 and 2016: observed rates (dots), fitted rates (lines) on log-scale, with 95% credible intervals for the fitted rates.**

## S5 Table. Estimated coefficients for the best fitting model for breast cancer rates.

| Covariate | Parameter | Mean | SD | %2.5 | %97.5 | Covariate | Parameter | Mean | SD | %2.5 | %97.5 |
|---|---|---|---|---|---|---|---|---|---|---|---|
| Intercept | $\beta_0$ | -5.9500 | 0.0047 | -5.9590 | -5.9410 | Year $<$ 1990 : Region | $\beta_{10,1,\text{region}_3}$ | -0.0027 | 0.0082 | -0.0190 | 0.0133 |
| Age $<$ 50 | $\beta_{1,1}$ | 1.1290 | 0.0303 | 1.0780 | 1.1870 | | $\beta_{10,1,\text{region}_4}$ | 0.0078 | 0.0086 | -0.0094 | 0.0239 |
| Age $\geq$ 50 | $\beta_{1,2}$ | 0.4595 | 0.0237 | 0.4233 | 0.5053 | | $\beta_{10,1,\text{region}_5}$ | -0.0256 | 0.0086 | -0.0428 | -0.0086 |
| Year $<$ 1990 | $\beta_{2,1}$ | 0.3152 | 0.0184 | 0.2812 | 0.3532 | | $\beta_{10,1,\text{region}_6}$ | 0.0025 | 0.0083 | -0.0139 | 0.0187 |
| Year $\geq$ 1990 | $\beta_{2,2}$ | 0.0848 | 0.0062 | 0.0725 | 0.0972 | | $\beta_{10,1,\text{region}_7}$ | -0.0033 | 0.0079 | -0.0189 | 0.0121 |
| Region | $\beta_{3,\text{region}_1}$ | -0.0540 | 0.0100 | -0.0735 | -0.0342 | | $\beta_{10,1,\text{region}_8}$ | -0.0080 | 0.0079 | -0.0232 | 0.0077 |
| | $\beta_{3,\text{region}_2}$ | -0.0261 | 0.0086 | -0.0424 | -0.0090 | | $\beta_{10,1,\text{region}_9}$ | 0.0031 | 0.0085 | -0.0133 | 0.0199 |
| | $\beta_{3,\text{region}_3}$ | -0.0430 | 0.0087 | -0.0598 | -0.0251 | Year $\geq$ 1990 : Region | $\beta_{10,2,\text{region}_1}$ | 0.0400 | 0.0078 | 0.0245 | 0.0551 |
| | $\beta_{3,\text{region}_4}$ | -0.0204 | 0.0085 | -0.0374 | -0.0035 | | $\beta_{10,2,\text{region}_2}$ | 0.0276 | 0.0063 | 0.0153 | 0.0402 |
| | $\beta_{3,\text{region}_5}$ | 0.0062 | 0.0093 | -0.0112 | 0.0247 | | $\beta_{10,2,\text{region}_3}$ | 0.0095 | 0.0068 | -0.0038 | 0.0229 |
| | $\beta_{3,\text{region}_6}$ | 0.0399 | 0.0084 | 0.0239 | 0.0565 | | $\beta_{10,2,\text{region}_4}$ | 0.0128 | 0.0068 | -0.0002 | 0.0266 |
| | $\beta_{3,\text{region}_7}$ | -0.0207 | 0.0089 | -0.0388 | -0.0034 | | $\beta_{10,2,\text{region}_5}$ | -0.0134 | 0.0067 | -0.0263 | -0.0003 |
| | $\beta_{3,\text{region}_8}$ | 0.0762 | 0.0091 | 0.0593 | 0.0953 | | $\beta_{10,2,\text{region}_6}$ | -0.0207 | 0.0066 | -0.0340 | -0.0079 |
| | $\beta_{3,\text{region}_9}$ | 0.0419 | 0.0092 | 0.0238 | 0.0596 | | $\beta_{10,2,\text{region}_7}$ | -0.0329 | 0.0066 | -0.0462 | -0.0199 |
| (Age $<$ 50)$^2$ | $\beta_{4,1}$ | -0.8318 | 0.0885 | -0.9685 | -0.6840 | | $\beta_{10,2,\text{region}_8}$ | -0.0245 | 0.0063 | -0.0366 | -0.0118 |
| (Age $\geq$ 50)$^2$ | $\beta_{4,2}$ | -0.4620 | 0.0355 | -0.5244 | -0.4105 | | $\beta_{10,2,\text{region}_9}$ | 0.0016 | 0.0067 | -0.0118 | 0.0146 |
| (Age $<$ 50)$^3$ | $\beta_{5,1}$ | 0.7355 | 0.0582 | 0.6440 | 0.8223 | Age $<$ 50 : Region | $\beta_{11,1,\text{region}_1}$ | -0.0169 | 0.0246 | -0.0678 | 0.0295 |
| (Age $\geq$ 50)$^3$ | $\beta_{5,2}$ | 0.2077 | 0.0141 | 0.1871 | 0.2315 | | $\beta_{11,1,\text{region}_2}$ | 0.0006 | 0.0170 | -0.0330 | 0.0343 |
| (Year $<$ 1990)$^2$ | $\beta_{6,1}$ | -0.0205 | 0.0123 | -0.0416 | 0.0055 | | $\beta_{11,1,\text{region}_3}$ | -0.0756 | 0.0194 | -0.1097 | -0.0342 |
| (Year $\geq$ 1990)$^2$ | $\beta_{6,2}$ | -0.0323 | 0.0037 | -0.0398 | -0.0254 | | $\beta_{11,1,\text{region}_4}$ | -0.0192 | 0.0192 | -0.0551 | 0.0187 |
| Age $<$ 50 : Year $<$ 1990 | $\beta_{7,1}$ | 0.5585 | 0.0826 | 0.3935 | 0.6796 | | $\beta_{11,1,\text{region}_5}$ | 0.0102 | 0.0193 | -0.0267 | 0.0492 |
| Age $<$ 50 : Year $\geq$ 1990 | $\beta_{7,2}$ | -0.0571 | 0.0676 | -0.1968 | 0.0343 | | $\beta_{11,1,\text{region}_6}$ | 0.0517 | 0.0190 | 0.0132 | 0.0872 |
| Age $\geq$ 50 : Year $<$ 1990 | $\beta_{7,3}$ | -0.1486 | 0.0353 | -0.2132 | -0.0827 | | $\beta_{11,1,\text{region}_7}$ | 0.0122 | 0.0178 | -0.0224 | 0.0454 |
| Age $\geq$ 50 : Year $\geq$ 1990 | $\beta_{7,4}$ | 0.1545 | 0.0257 | 0.0984 | 0.1992 | | $\beta_{11,1,\text{region}_8}$ | 0.0361 | 0.0169 | 0.0054 | 0.0693 |
| (Age $<$ 50)$^2$ : Year $<$ 1990 | $\beta_{8,1}$ | 0.1970 | 0.2176 | -0.1573 | 0.5059 | | $\beta_{11,1,\text{region}_9}$ | 0.0009 | 0.0193 | -0.0352 | 0.0401 |
| (Age $<$ 50)$^2$ : Year $\geq$ 1990 | $\beta_{8,2}$ | -0.0732 | 0.1798 | -0.4121 | 0.1634 | Age $\geq$ 50 : Region | $\beta_{11,2,\text{region}_1}$ | -0.0543 | 0.0102 | -0.0747 | -0.0344 |
| (Age $\geq$ 50)$^2$ : Year $<$ 1990 | $\beta_{8,3}$ | -0.0226 | 0.0569 | -0.1284 | 0.0754 | | $\beta_{11,2,\text{region}_2}$ | -0.0269 | 0.0088 | -0.0444 | -0.0094 |
| (Age $\geq$ 50)$^2$ : Year $\geq$ 1990 | $\beta_{8,4}$ | 0.0027 | 0.0391 | -0.0766 | 0.0807 | | $\beta_{11,2,\text{region}_3}$ | -0.0127 | 0.0085 | -0.0298 | 0.0039 |
| (Age $<$ 50)$^3$ : Year $<$ 1990 | $\beta_{9,1}$ | -0.1213 | 0.1323 | -0.3227 | 0.0763 | | $\beta_{11,2,\text{region}_4}$ | 0.0296 | 0.0089 | 0.0119 | 0.0475 |
| (Age $<$ 50)$^3$ : Year $\geq$ 1990 | $\beta_{9,2}$ | -0.0310 | 0.1087 | -0.2238 | 0.1187 | | $\beta_{11,2,\text{region}_5}$ | 0.0109 | 0.0091 | -0.0071 | 0.0285 |
| (Age $\geq$ 50)$^3$ : Year $<$ 1990 | $\beta_{9,3}$ | 0.0254 | 0.0232 | -0.0142 | 0.0690 | | $\beta_{11,2,\text{region}_6}$ | 0.0066 | 0.0088 | -0.0107 | 0.0240 |
| (Age $\geq$ 50)$^3$ : Year $\geq$ 1990 | $\beta_{9,4}$ | -0.0497 | 0.0157 | -0.0793 | -0.0164 | | $\beta_{11,2,\text{region}_7}$ | 0.0212 | 0.0088 | 0.0045 | 0.0386 |
| Year $<$ 1990 : Region | $\beta_{10,1,\text{region}_1}$ | 0.0203 | 0.0100 | 0.0009 | 0.0399 | | $\beta_{11,2,\text{region}_8}$ | -0.0111 | 0.0089 | -0.0289 | 0.0053 |
| | $\beta_{10,1,\text{region}_2}$ | 0.0059 | 0.0080 | -0.0104 | 0.0216 | | $\beta_{11,2,\text{region}_9}$ | 0.0367 | 0.0090 | 0.0190 | 0.0550 |
| | | | | | | | $\sigma^2$ | 0.0058 | 0.0002 | 0.0053 | 0.0062 |

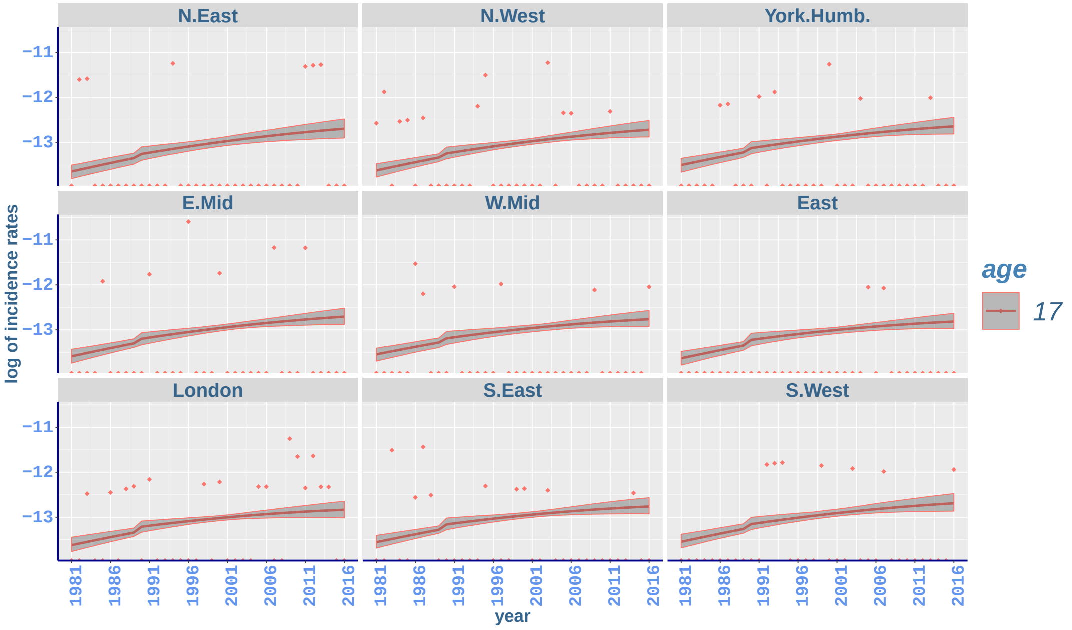


**S52 Fig. Breast cancer incidence, age 17: observed rates (dots), fitted rates (lines) on log-scale, with 95% credible intervals for the fitted rates.**

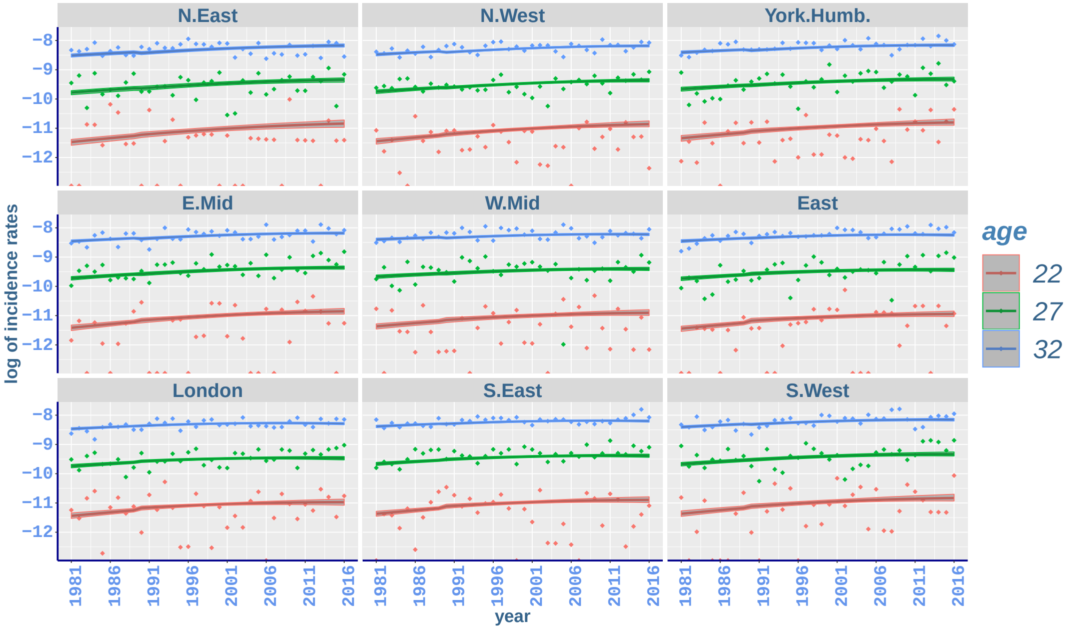


**S53 Fig. Breast cancer incidence, ages 22-32: observed rates (dots), fitted rates (lines) on log-scale, with 95% credible intervals for the fitted rates.**

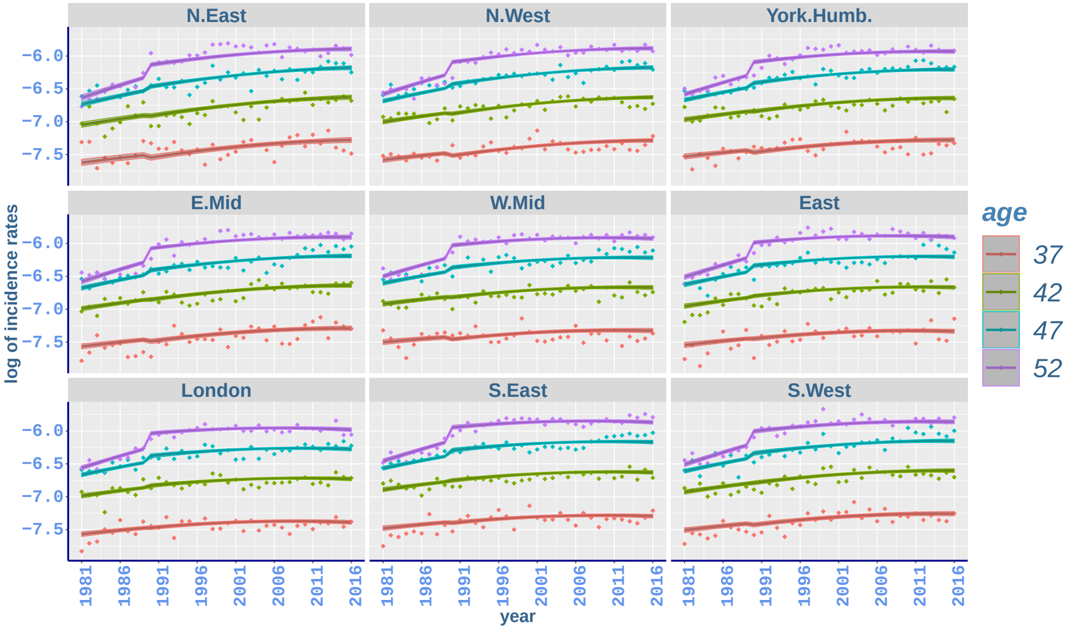


S54 Fig. Breast cancer incidence, ages 37-52: observed rates (dots), fitted rates (lines) on log-scale, with 95% credible intervals for the fitted rates.

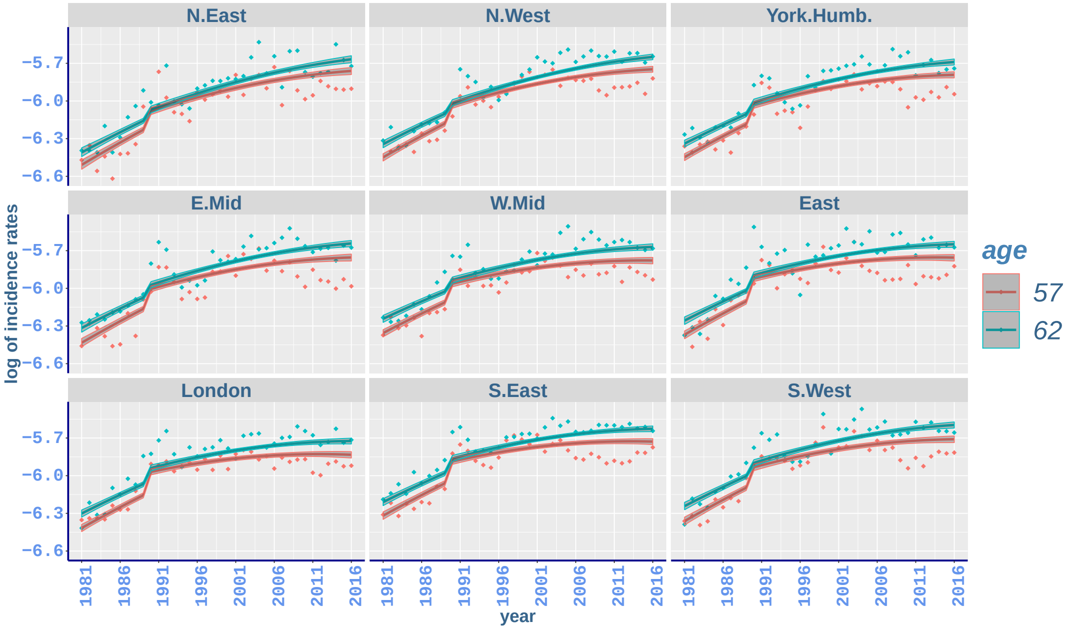


S55 Fig. Breast cancer incidence, ages 57-62: observed rates (dots), fitted rates (lines) on log-scale, with 95% credible intervals for the fitted rates.

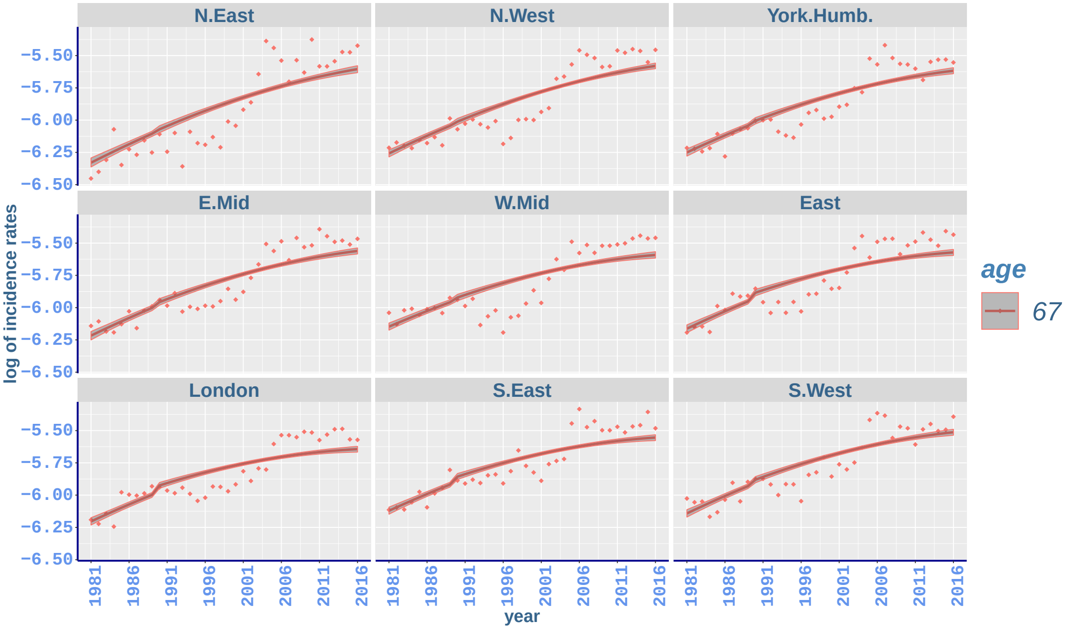


**S56 Fig. Breast cancer incidence, age 67: observed rates (dots), fitted rates (lines) on log-scale, with 95% credible intervals for the fitted rates.**

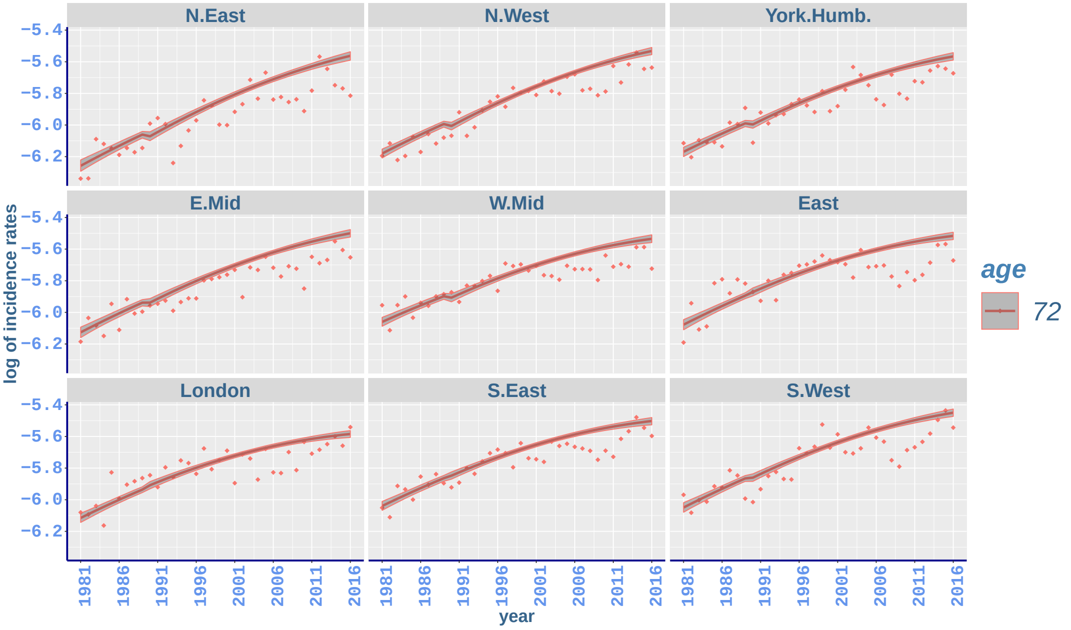


**S57 Fig. Breast cancer incidence, age 72: observed rates (dots), fitted rates (lines) on log-scale, with 95% credible intervals for the fitted rates.**

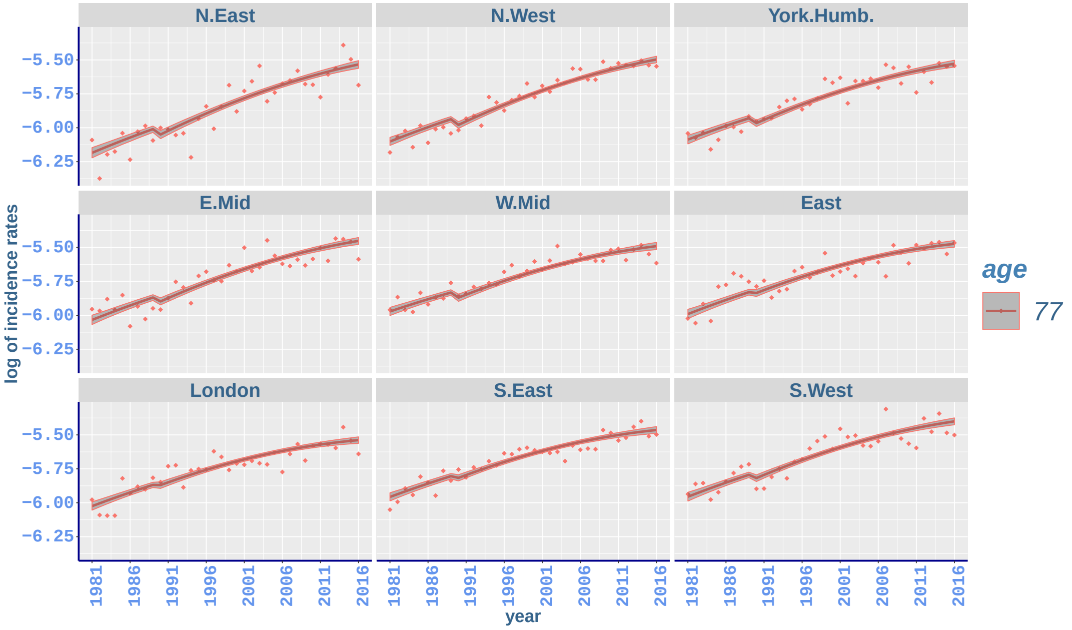


**S58 Fig. Breast cancer incidence, age 77: observed rates (dots), fitted rates (lines) on log-scale, with 95% credible intervals for the fitted rates.**

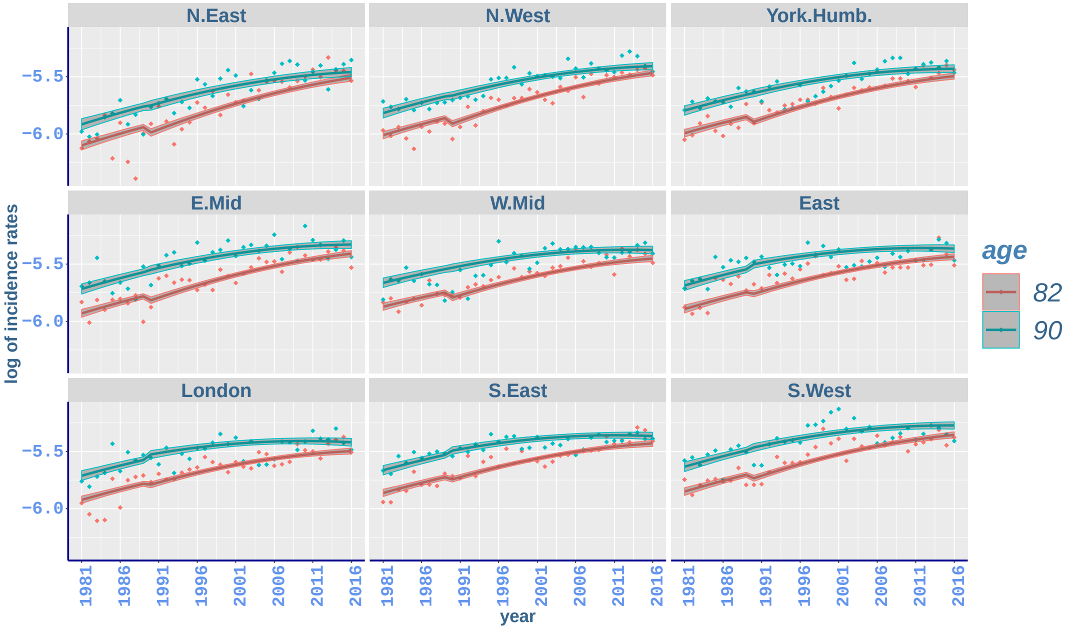


**S59 Fig. Breast cancer incidence, ages 82-90: observed rates (dots), fitted rates (lines) on log-scale, with 95% credible intervals for the fitted rates.**

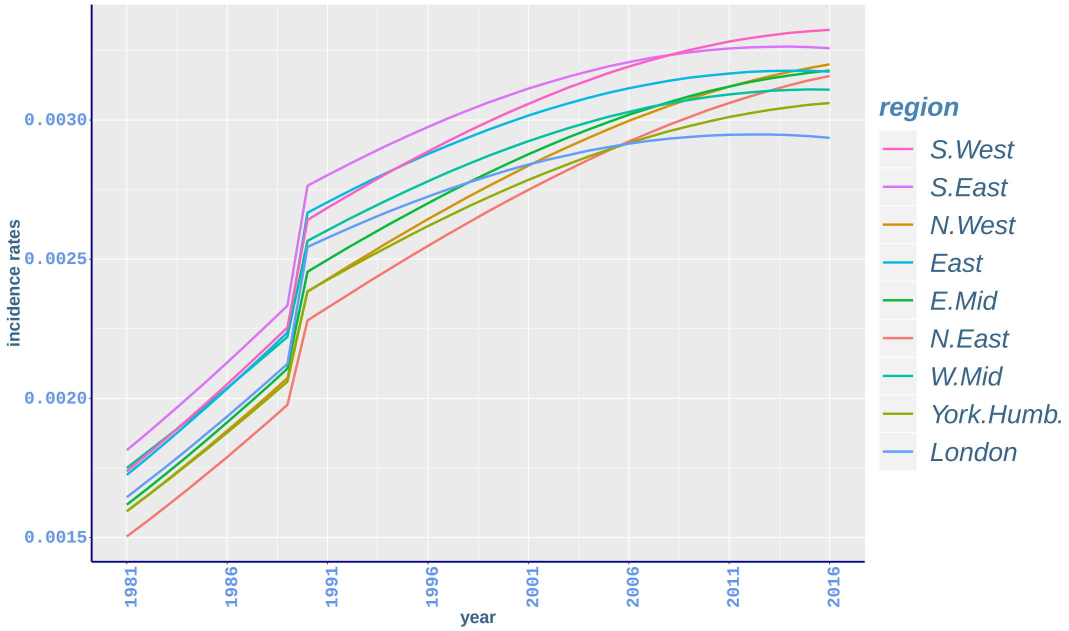


**S60 Fig. Breast cancer incidence, age 57: fitted rates.**

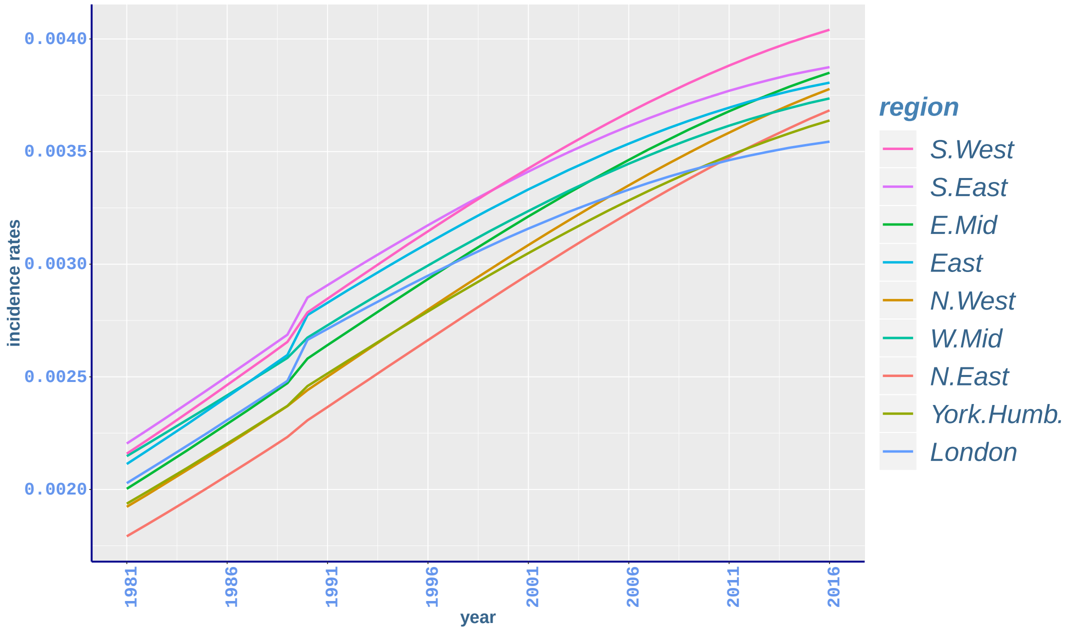


**S61 Fig. Breast cancer incidence, age 67: fitted rates.**

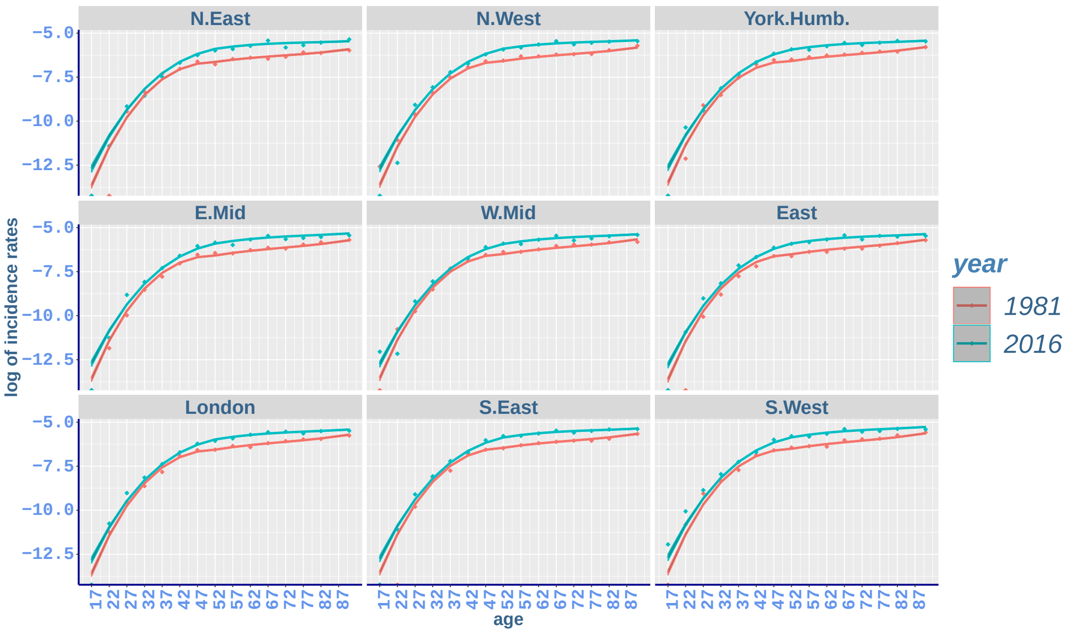


**S62 Fig. Breast cancer incidence, year 1981 and 2016: observed rates (dots), fitted rates (lines) on log-scale, with 95% credible intervals for the fitted rates.**
